# Synthesis, Antifungal Activity, and 3D-QSAR Study of Novel Nopol-Derived 1,3,4-Thiadiazole-Thiourea Compounds

**DOI:** 10.3390/molecules26061708

**Published:** 2021-03-18

**Authors:** Ming Chen, Wen-Gui Duan, Gui-Shan Lin, Zhong-Tian Fan, Xiu Wang

**Affiliations:** School of Chemistry and Chemical Engineering, Guangxi University, Nanning 530004, China; chenminggxu@163.com (M.C.); fztt187@163.com (Z.-T.F.); wangxiumt2015@163.com (X.W.)

**Keywords:** *β*-pinene, nopol, 1,3,4-thiadiazole, thiourea, antifungal activity, 3D-QSAR

## Abstract

A series of novel nopol derivatives bearing the 1,3,4-thiadiazole-thiourea moiety were designed and synthesized by multi-step reactions in search of potent natural product-based antifungal agents. Their structures were confirmed by FT-IR, NMR, ESI-MS, and elemental analysis. Antifungal activity of the target compounds was preliminarily evaluated by in vitro methods against *Fusarium oxysporum* f. sp. *cucumerinum*, *Cercospora arachidicola*, *Physalospora piricola*, *Alternaria solani*, *Gibberella zeae*, *Rhizoeotnia solani*, *Bipolaris maydis*, and *Colleterichum orbicalare* at 50 µg/mL. All the target compounds exhibited better antifungal activity against *P. piricola*, *C. arachidicola*, and *A. solani*. Compound **6j** (R = *m*, *p*-Cl Ph) showed the best broad-spectrum antifungal activity against all the tested fungi. Compounds **6c** (R = *m*-Me Ph), **6q** (R = *i*-Pr), and **6i** (R = *p*-Cl Ph) had inhibition rates of 86.1%, 86.1%, and 80.2%, respectively, against *P. piricola*, much better than that of the positive control chlorothalonil. Moreover, compounds **6h** (R = *m*-Cl Ph) and **6n** (R = *o*-CF_3_ Ph) held inhibition rates of 80.6% and 79.0% against *C. arachidicola* and *G. zeae*, respectively, much better than that of the commercial fungicide chlorothalonil. In order to design more effective antifungal compounds against *A. solani*, analysis of the three-dimensional quantitative structure–activity relationship (3D-QSAR) was carried out using the CoMFA method, and a reasonable and effective 3D-QSAR model (*r*^2^ = 0.992, *q*^2^ = 0.753) has been established. Furthermore, some intriguing structure–activity relationships were found and are discussed by theoretical calculation.

## 1. Introduction

Turpentine oil, which was obtained by the steam distillation of the oleoresin exudate from living pine trees, is an extremely abundant natural biomass resource in nature, and is widely used in the synthesis of fine chemicals such as food additives, pharmaceuticals, agrochemicals, and flavors [1]. As one of the major components, *β*-pinene content can be as high as about 30% in raw turpentine oils [2]. Therefore, it is an important research topic to convert *β*-pinene into high value-added fine chemicals.

**Figure 1:**
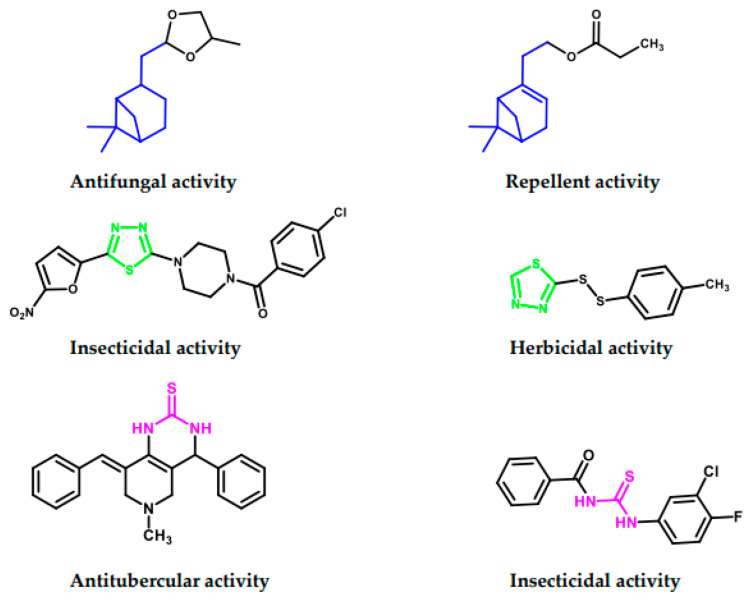
Bioactive molecules containing nopol, 1,3,4-thiadiazole and thiourea moieties.

Nopol, with the chemical name of 2-(6,6-dimethyl-2-bicyclo [3.1.1] hept-2-enyl) ethanol, is an optically active bicyclic primary alcohol, and contains three reactive functional groups, including a hydroxyl group, a carbon–carbon double bond, and a four-membered ring. It is usually prepared by a Prins condensation reaction of *β*-pinene with paraformaldehyde and is generally used in the flavor and fragrance industries, in the agrochemical industry to produce pesticides, as well as in the manufacture of soaps, detergents, polishes, and other household products [3]. Nopol and its derivatives were found to exhibit a broad spectrum of bioactivities, such as insecticidal, antifungal [4], antitumor [5], and repellent [6] characteristics, as well as for treatment of irritable bowel syndrome [7] (see examples in Figure 1).

On the other hand, 1,3,4-thiadiazole derivatives exhibit various biological properties, such as insecticidal [8], herbicidal [9], antibacterial [10,11,12], antiviral [13], antifungal [14], antitumor [15,16] and plant growth regulation [17] activities (see examples in Figure 1). In addition, thiourea derivatives have considerable applications in agriculture, gold leaching processes, analytical chemistry, and medicine [18,19,20]. Compounds with thiourea scaffolds hold diverse pharmacological properties such as insecticidal [21], antifungal [22], herbicidal [23], antibacterial [24], antitubercular [25], antithyroid [26], and antimicrobial [27] activities (see examples in Figure 1).

In continuation of our long-term focus on natural product-based bioactive compounds [28,29,30,31,32], a series of novel nopol-derived 1,3,4-thiadiazole-thiourea compounds were synthesized by integrating bioactive 1,3,4-thiadiazole and thiourea moieties into the molecular skeleton of nopol. Structural characterization, antifungal evaluation, and structure–activity relationships of the title compounds were determined as well.

## 2. Results and Discussion

### 2.1. Synthesis and Characterization

**Scheme 1:**
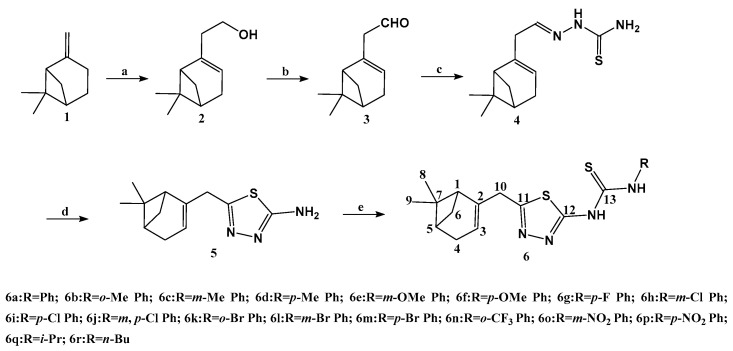
Synthesis of nopol-derived 1,3,4-thiadiazole-thiourea compounds **6a**–**6r**. Reagents and conditions: (**a**) (CH_2_O)_n_, ZnCl_2_, 75 °C, 1 h; 110 °C, 12 h; (**b**) Dess–Martin periodinane, NaHCO_3_, 0 °C, 4 h; (**c**) thiosemicarbazide, H_2_O, EtOH, 65 °C, 15 min; (**d**) I_2_, K_2_CO_3_, 1,4-dioxane, 80 °C, 6 h; (**e**) RNCS, NaOH, 1,4-dioxane, 90 °C, 8–12 h.

As illustrated in Scheme 1, nopol (**2**) was prepared by the Prins reaction of *β*-pinene (**1**) with paraformaldehyde, and further oxidized to obtain nopol aldehyde (**3**). Then, nopol-based thiosemicarbazone (**4**) was prepared by a condensation reaction of compound **3** with thiosemicarbazide, followed by a ring-closing reaction to produce 5-nopyl-2-amino-1,3,4-thiadiazole (**5**). Lastly, a series of target compounds, **6a**–**6r**, were synthesized by the nucleophilic addition reaction of compound **5** with different isothiocyanates.

The structures of the target compounds were characterized by means of FT-IR, ^1^H-NMR, ^13^C-NMR, ESI-MS, and elemental analysis. In the FT-IR spectra, the target compounds exhibited moderate absorption bands at 3294–3330 cm^−1^, which were assigned to the N–H stretching vibration. The weak absorption bands at 1632–1671 cm^−1^ were attributed to the stretching vibrations of the unsaturated C=C in the nopol moiety. The strong absorption bands at 1524–1586 cm^−1^ and the moderate absorption bands at 646–660 cm^−1^ were assigned to the vibrations of C=N and C–S–C in 1,3,4-thiadiazole moiety, respectively. The three strong absorption bands around 1361–1440 cm^−1^, 1326–1390 cm^−1^, and 1077–1091 cm^−1^ confirmed the presence of thiourea groups. In the ^1^H-NMR spectra, the olefinic protons of nopol scaffolds showed signals at about 5.48–5.54 ppm, and the other protons bonded to the saturated carbons of the nopol moiety displayed signals in the range of 0.75–3.74 ppm. Signals of the N–H protons were located at δ13.27–14.49 ppm and 7.54–10.93 ppm, respectively. The ^13^C-NMR spectra of the target compounds showed peaks for the olefinic carbons of the nopol moiety at 120.4–121.2 ppm, and the other saturated carbons displayed signals in the region of 21.1–45.3 ppm. For the 1,3,4-thiadiazole moiety, the signals at 157.0–172.0 ppm and 143.7–161.9 ppm were assigned to the carbons of C=N. The carbons of C=S in the thiourea moiety displayed the signals at 177.2–188.0 ppm. Their molecular weights and the C, H, and N element ratios were confirmed by ESI-MS and elemental analysis, respectively (see Supplementary Materials).

### 2.2. Antifungal Activity

The antifungal activity of the target compounds **6a**–**6r** was evaluated by in vitro method against fusarium wilt on cucumber (*Fusarium oxysporum* f. sp. *cucumerinum*), speckle on peanut (*Cercospora arachidicola*), apple ring rot (*Physalospora piricola*), tomato early blight (*Alternaria solani*), wheat scab (*Gibberella zeae*), rice sheath blight (*Rhizoeotnia solani*), corn southern leaf blight (*Bipolaris maydis*), and watermelon anthracnose (*Colleterichum orbicalare*) at concentrations of 50 μg/mL, using the commercial antifungal agent chlorothalonil as positive control. The results are listed in Table 1.

It was found that, at 50 µg/mL, the target compounds showed certain antifungal activity against the eight tested fungi. On the whole, all the target compounds exhibited better antifungal activity against *P. piricola*, *C. arachidicola*, and *A. solani*, with average inhibition rates of 65.7%, 64.3%, and 64.2%, respectively. Compound **6j** (R = *m*, *p*-Cl Ph) showed the best and broad-spectrum antifungal activity against all the tested fungi, with an average inhibition activity of 51.9%. Compounds **6c** (R = *m*-Me Ph), **6q** (R = *i*-Pr), and **6i** (R = *p*-Cl Ph) had inhibition rates of 86.1%, 86.1%, and 80.2%, respectively, against *P. piricola*, much better than the of the positive control chlorothalonil. Besides, compounds **6h** (R = *m*-Cl Ph) and **6n** (R = *o*-CF_3_ Ph) held inhibition rates of 80.6% and 79.0% against *C. arachidicola* and *G. zeae*, respectively, much better than that of the positive control chlorothalonil. In order to investigate the structure–activity relationship, 3D-QSAR studies and theoretical calculations were subsequently performed.

### 2.3. CoMFA Analysis

**Figure 2:**
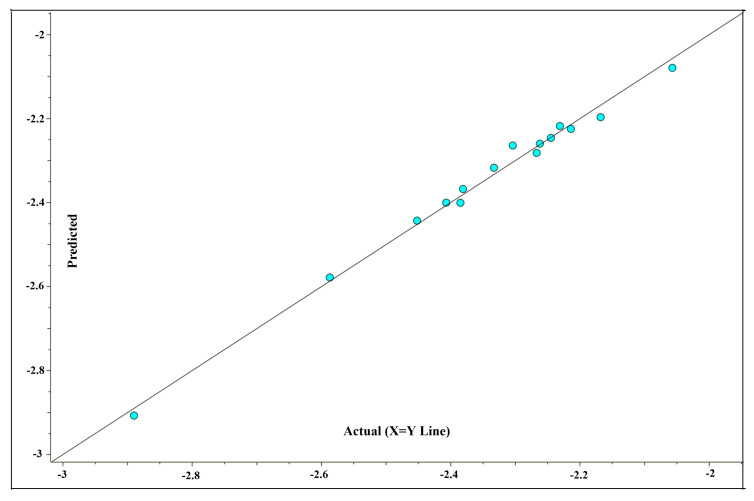
Predicted ED values of CoMFA model vs experimental ED value.

The 3D-QSAR analysis of the antifungal activity against *A. solani* of the target compounds was carried out by the CoMFA method. In this work, fifteen target compounds in the training set and one compound in the test set are presented in Table 2. A predictive 3D-QSAR model with the value of non-validated correlational coefficient (*r*^2^ = 0.992), the Fischer ratio (*F* = 294.200), standard error of estimate (*S* = 0.021) and the cross-validated correlational coefficient (*q*^2^ = 0.753) was established (Table 3). As shown in Figure 2, the Scatter plot of predicted ED values vs experimental ED values is presented, and all data were concentrated near the X = Y line, indicating that the 3D-QSAR model was reasonable and effective.

**Figure 3:**
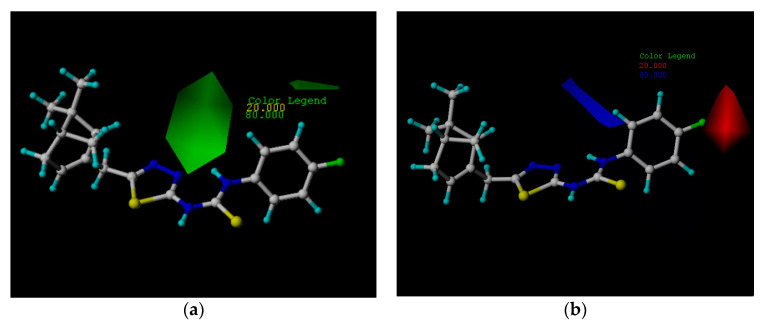
Contours of CoMFA analysis: (**a**) contours of steric contribution are represented in yellow and green; (**b**) contours of electrostatic contribution are represented in red and blue.

The electrostatic and steric contribution maps of CoMFA are shown in Figure 3. The contribution rate of the electrostatic field was 47.1% while the steric field was 52.9%, which revealed that the steric field was the major contributor to the increase in the antifungal activity against *A. Solani*. In Figure 3a, there were some green regions located around the 2-position and 3-position of the benzene ring; the green area indicated that the introduction of large groups was beneficial to increasing the antifungal activity. For example, compounds **6c** (R = *m*-Me Ph), **6h** (R = *m*-Cl Ph), **6l** (R = *m*-Br Ph), **6n** (R = *o*-CF_3_ Ph) and **6o** (R = *m*-NO_2_ Ph) displayed higher antifungal activity than compound **6a** (R = Ph). In Figure 3b, the electrostatic field contours were displayed in two distinguishing colors. The blue area indicated that the introduction of electron-donating groups was beneficial to improving activity, and the red area indicated that the introduction of electron-withdrawing groups was beneficial to improving activity. Therefore, the introduction of electron-donating groups at the 2-position and electron-withdrawing groups at the 4-position of benzene rings were both favorable for antifungal activity. For instance, compound **6b** (R = *o*-Me Ph) exhibited better antifungal activity than that of **6k** (R = *o*-Br Ph), compounds **6g** (R = *p*-F Ph), and **6i** (R = *p*-Cl Ph), and **6m** (R = *p*-Br Ph) possessed a higher inhibitory rate than **6d** (R = *p*-Me Ph) and **6f** (R = *p*-OMe Ph).

**Figure 4:**
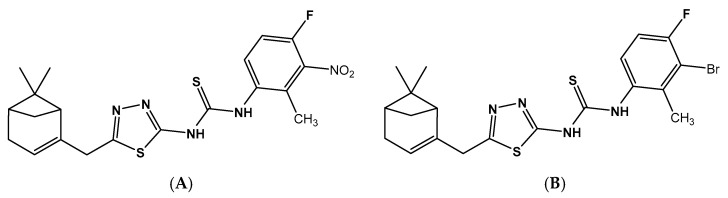
The proposed new molecules based on the established CoMFA model: 1-(5-((6,6-dimethylbicyclo[3.1.1]hept-2-en-2-yl)methyl)-1,3,4-thiadiazol-2-yl)-3-(4-fluoro-2-methyl-3-nitrophenyl) thiourea (**A**); 1-(3-bromo-4-fluoro-2-methylphenyl)-3-(5-((6,6-dimethylbicyclo[3.1.1]hept-2-en-2-yl)methyl)-1,3,4-thiadiazol-2-yl) thiourea (**B**).

The established CoMFA model could be used to predict the activity of new candidate compounds prior to their synthesis. Herein, based on 3D-QSAR analysis above, two novel unsynthesized compounds (Figure 4) were designed and the predicted ED values were calculated by the established CoMFA model. As a result, the designed compounds **A** (ED^[a]^ = −1.790) and **B** (ED^[a]^ = −1.806) showed potential excellent antifungal activities with inhibition rates of 87.9% and 88.2%, respectively, which deserved further study.

### 2.4. Theoretical Calculation and Analysis

**Figure 5:**
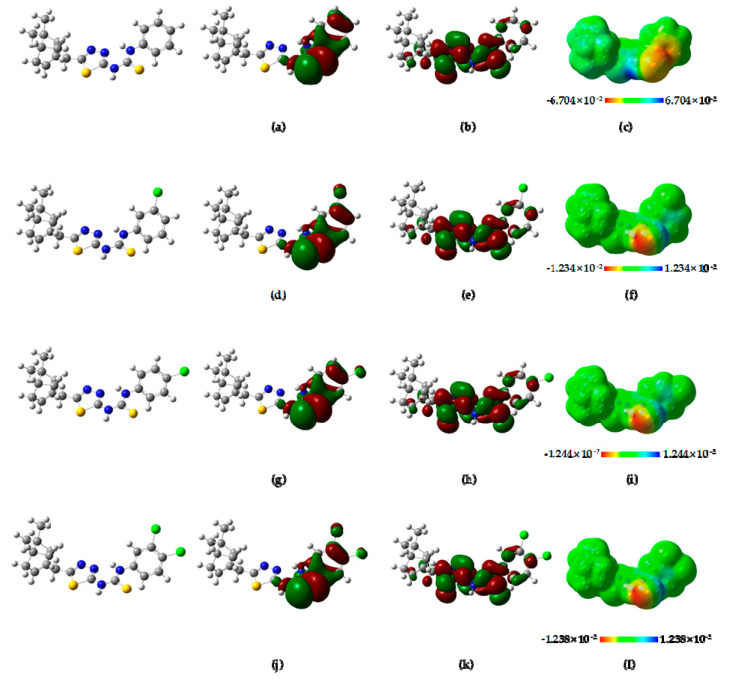
Frontier molecular orbitals and electrostatic potentials (ESPs) of compounds **6a** (R = Ph): HOMO (**a**), LUMO (**b**), and ESP (**c**); **6h** (R = *m*-Cl Ph): HOMO (**d**), LUMO (**e**), and ESP (**f**); **6i** (R = *p*-Cl Ph): HOMO (**g**), LUMO (**h**), and ESP (**i**); **6j** (R = *m, p*-Cl Ph): HOMO (**j**), LUMO (**k**), and ESP (**l**).

Among the title compounds, disubstituted compound **6j** (R = *m*, *p*-Cl Ph) showed the best broad-spectrum antifungal activity with an average inhibition activity of 51.9% against all the tested fungi. Its corresponding unsubstituted compound **6a** (R = Ph), and monosubstituted compounds **6h** (R = *m*-Cl Ph) and **6i** (R = *p*-Cl Ph) showed lower activity, with average inhibition rates of 46.4%, 47.8%, 48.5%, respectively. According to the frontier molecular orbital theory, HOMO can provide electrons, while LUMO readily accepts electrons; thus, these two frontier orbitals can affect the bioactivity of compounds [33]. Meanwhile, the properties of molecule such as electrostatic potentials (ESPs) and dipole moments can also affect the bioactivity of compounds. Therefore, the frontier molecular orbitals, ESPs, and dipole moments for compounds **6a** (R = Ph), **6h** (R = *m*-Cl Ph), **6i** (R = *p*-Cl Ph), and **6j** (R = *m, p*-Cl Ph) were calculated by means of DFT/B3LYP/6-31G (d, p) in the Gaussian 09 package on the Supercomputing Platform at Guangxi University, and the result was viewed using the GaussView 5 software [34]. The result is showed in Figure 5. 

As shown in Figure 5a,d,g,j, a large portion of the HOMO was located on the thiourea group and the substituted benzene ring, with similar distribution and phase for the four compounds. The LUMO for these compounds was located on the 1,3,4-thiadiazole ring, the thiourea group and the substituted benzene ring, and shared the same case (Figure 5b,e,h,k). The dipole moments for the compounds **6a** (R = Ph), **6h** (R = *m*-Cl Ph), **6i** (R = *p*-Cl Ph) and **6j** (R = *m, p*-Cl Ph) were 5.7380 D, 6.5806 D, 7.6854 D and 8.2789 D, respectively, which showed corresponding correlation with the rank of their antifungal activity. The dipole moment had a correlation with the ESP of molecules. The ESPs for these compounds are shown in Figure 5c,f,i,l. The chlorine atoms of these molecules displayed a negative electrostatic potential. The chlorine atoms at meta and para positions of the benzene ring could increase the dipole moment value, resulting in the improvement of antifungal activity. These results would be useful for further investigating in these compounds.

## 3. Experimental Section

### 3.1. General Information

HPLC analysis was conducted on Waters 1525 HPLC instrument (Waters Co., Ltd., Milford, MA, USA) equipped with Waters 2998 PDA detector and column C18 5 μm (4.6 mm × 150 mm). GC analysis was conducted on Agilent 6890 GC (Agilent Technologies Inc., Santa Clara, CA, USA) equipped with column HP-1 (30 m, 0.530 mm, 0.88 μm) and FID. Melting points were recorded using an MP420 automatic melting point apparatus (Hanon Instruments Co., Ltd., Jinan, China) and were not corrected. IR spectra were recorded on a Nicolet iS50 FT-IR spectrometer (Thermo Scientific Co., Ltd., Madison, WI, USA) by the KBr pellet method. NMR spectra were determined in a CDCl_3_ or DMSO-*d*_6_ solvent on a Bruker Avance III HD 500 MHz/600 MHz spectrometer (Switzerland Bruker Co., Ltd., Zurich, Switzerland), and chemical shifts are expressed in (δ) ppm downfield relative to TMS as an internal standard. MS spectra were obtained by means of the electrospray ionization (ESI) method on the TSQ Quantum Access MAX HPLC-MS instrument (Thermo Scientific Co., Ltd., Waltham, MA, USA). Elemental analyses were measured using a PE 2400 II elemental analyzer (Perkin-Elmer Instruments Co., Ltd., Waltham, MA, USA). *β*-Pinene (GC purity 98%) was provided by Jiangxi Cedar Natural Medicinal Oil Co., Ltd., Jian, China. Other reagents were purchased from commercial suppliers and used as received.

### 3.2. Synthesis of Nopol (***2***)

*β*-Pinene **1** (133.50 g, 980 mmol) and paraformaldehyde (42.84 g, 476 mmol) were mixed and stirred at 75 °C. Then, ZnCl_2_ (1.50 g, 11.0 mmol) was slowly added and stirred for 1 h. Afterwards, the reaction mixture was stirred at 110 °C for 12 h. The reaction process was monitored by TLC. When the reaction was completed, the mixture was cooled to room temperature. The organic layer was separated, washed three times with water, dried over anhydrous Na_2_SO_4_, and distilled in a vacuum to give compound **2** as a colorless transparent liquid. Yield 60%; FT-IR (KBr, cm^−1^): 3369 (–OH), 3030 (=CH), 1471 (CH_2_), 1386, 1368 (C(CH_3_)_2_), 1048 (C–O); ^1^H-NMR (600 MHz, CDCl_3_): δ 5.33 (dt, *J* = 4.2, 1.3 Hz, 1H), 3.60 (d, *J* = 5.4 Hz, 2H), 2.38 (dt, *J* = 8.6, 5.6 Hz, 1H), 2.28 (d, *J* = 17.5 Hz, 1H), 2.24–2.19 (m, 3H), 2.12–2.08 (m, 1H), 2.03 (td, *J* = 5.6, 1.4 Hz), 1.49 (s, 1H), 1.27 (s, 3H), 1.14 (d, *J* = 8.6 Hz), 0.84 (s, 3H); ^13^C-NMR (151 MHz, CDCl_3_): δ 144.7, 119.4, 60.0, 45.6, 40.7, 40.2, 37.9, 31.8, 31.4, 26.3, 21.2.

### 3.3. Synthesis of Nopol Aldehyde (***3***) 

Compound **3** was prepared according to a method described in the literature [35]. Nopol **2** (1.66 g, 10 mmol), Dess–Martin periodinane (5.51g, 13 mmol) and NaHCO_3_ (2.18 g, 26 mmol) were dissolved in CH_2_Cl_2_ (40 mL) and stirred at 0 °C for 4 h. Upon completion, the filtrate was collected by filtration and washed three times with 5% Na_2_S_2_O_3_ (3 × 20 mL), then dried over anhydrous Na_2_SO_4_, and evaporated in vacuo to give compound **3** as a colorless transparent liquid. Yield 75%; FT-IR (KBr, cm^−1^): 3030 (C=C–H), 2768, 2733 (O=C–H), 1671 (C=O), 1623 (C=C); ESI-MS *m*/*z*: 165.08 ([M + H^+^]).

### 3.4. Synthesis of Nopol-Based Thiosemicarbazone (***4***)

Compound 4 was prepared by the method presented in our previous work [36]. To a stirred solution of thiosemicarbazide (0.91 g, 10 mmol) in distilled water (20.0 mL), a solution of nopol aldehyde 3 (1.64 g, 10 mmol) in anhydrous ethanol (20.0 mL) was added dropwise at 65 °C in 15 min. The reaction mixture was cooled to room temperature and filtrated. The resulting filter cake was washed several times with a mixture of distilled water and ethanol (distilled water–ethanol = 1:1, *v*/*v*) to give the crude product, which was purified by recrystallization in ethanol to obtain compound **4** as a white solid. Yield 85%; m.p.154.2–156.8 °C; FT-IR (KBr, cm^−1^): 3412, 3257, 3158 (N–H), 3030 (C=C–H), 1603 (C=C), 1535 (C=N), 1462, 1366, 1070 (N–C=S); ^1^H-NMR (600 MHz, CDCl_3_) δ 10.02 (s, 1H), 7.23 (t, *J* = 5.6 Hz, 1H), 7.08 (s, 1H), 6.49 (s, 1H), 5.32 (s, 1H), 2.89 (d, *J* = 5.4 Hz, 2H), 2.36 (dt, *J* = 8.5, 5.7 Hz, 1H), 2.23 (d, *J* = 25.7 Hz, 2H), 2.08 (s, 1H), 1.99 (t, *J* = 5.5 Hz, 1H), 1.25 (s, 3H), 1.15 (d, *J* = 8.6 Hz, 1H), 0.81 (s, 3H); ^13^C-NMR (151 MHz, CDCl_3_) δ 178.1, 146.3, 142.5, 120.2, 45.5, 40.6, 39.8, 38.1, 31.6, 31.4, 26.2, 21.2; ESI-MS *m*/*z*: 238.13 ([M + H^+^]).

### 3.5. Synthesis of 5-Nopyl-2-amino-1,3,4-thiadiazole (***5***)

As described in the literature [37], compound **4** (0.71 g, 3 mmol), iodine (0.91 g, 3.6 mmol), and potassium carbonate (1.24 g, 9 mmol) were added into dioxane (25 mL) at 80 °C and stirred for 6 h. The reaction process was monitored by TLC. Upon completion, the reaction mixture was cooled to room temperature, reacted with 5% Na_2_S_2_O_3_ (20 mL), and extracted with EtOAc. The upper layer with the desired organic compound was collected, dried over anhydrous Na_2_SO_4_, and evaporated in vacuo to obtain compound **5** as a brown solid. Yield 65%; m.p.167.9–169.1 °C; FT-IR (KBr, cm^−1^): 3297, 3101 (N–H), 3006 (=C–H), 1642 (C=C), 1520 (C=N); ^1^H-NMR (600 MHz, DMSO) δ 7.00 (s, 2H), 5.42 (s, 1H), 3.46 (q, *J* = 14.9 Hz, 2H), 2.35 (dt, *J* = 8.4, 5.6 Hz, 1H), 2.21 (dd, *J* = 52.9, 17.7 Hz, 2H), 2.05 (s, 1H), 2.02 (t, *J* = 5.2 Hz, 1H), 1.21 (s, 3H), 1.07 (d, *J* = 8.5 Hz, 1H), 0.75 (s, 3H); ^13^C- NMR (151 MHz, DMSO) δ 169.1, 156.7, 144.6, 119.6, 45.0, 40.4, 37.9, 37.5, 32.0, 31.3, 26.4, 21.4; ESI-MS *m*/*z*: 236.10 ([M + H^+^]). 

### 3.6. General Procedure for Synthesis of Nopol-Based 1,3,4-Thiadiazole-thiourea Compiunds (***6a***–***6r***)

To a mixture of compound **5** (0.24 g, 1 mmol) and sodium hydroxide (0.04 g, 1 mmol) in dry dioxane (20 mL), substituted isothiocyanate (1.2 mmol) was added slowly with vigorous stirring at room temperature. Then, the reaction mixture was heated to 90 °C and stirred for 8–12 h. After the reaction was completed, the residual sodium hydroxide was removed through filtration. The filtrate was concentrated in vacuo and the residue was purified by silica gel chromatography to afford the target compounds **6a**–**6r** in yields of 45%–70%. 

Compound(**6a**): *1-(5-((6,6-Dimethylbicyclo[3.1.1]hept-2-en-2-yl)methyl)-1,3,4-thiadiazol- 2-yl)-3-phenylthiourea*. White solid. Yield: 45.0%, m.p. 180.0–181.2 °C; FT-IR (KBr, cm^−1^): 3315 (N-H), 3024 (=C–H), 1667 (C=C), 1599, 1502 (Ar–C=C), 1553 (C=N), 1440, 1326, 1081 (N–C=S), 658 (C–S–C); ^1^H-NMR (500 MHz, DMSO) δ 13.99 (s, 1H, N–H), 10.40 (s, 1H, N–H), 7.68 (d, *J* = 7.9 Hz, 2H, C_15_-H, C_19_-H), 7.31 (t, *J* = 7.7 Hz, 2H, C_16_-H, C_18_-H), 7.08 (s, 1H, C_17_-H), 5.50 (s, 1H, C_3_-H), 3.58–3.50 (m, 2H, C_10_-H), 2.36 (dt, *J* = 8.4, 5.6 Hz, 1H, C_1_-H), 2.23 (dd, *J* = 49.3, 17.7 Hz, 2H, C_4_-H_a_, C_7_-H_a_), 2.05 (d, *J* = 4.9 Hz, 2H, C_7_-H_b_, C_5_-H), 1.22 (s, 3H, C_9_-CH_3_), 1.11 (d, *J* = 8.5 Hz, 1H, C_4_-H_b_), 0.76 (s, 3H, C_8_-CH_3_); ^13^C-NMR (126 MHz, DMSO) δ 184.6, 170.1, 156.7, 143.6, 140.2, 128.8, 124.3, 122.9, 120.6, 45.1, 40.4, 38.0, 37.6, 31.9, 31.4, 26.4, 21.4; ESI-MS *m*/*z*: 371.28 ([M + H^+^]). Anal. calcd. for C_19_H_22_N_4_S_2_: C, 61.59; H, 5.98; N, 15.12; Found: C, 61.56; H, 5.95; N:15.10.

Compound(**6b**): *1-(5-((6,6-Dimethylbicyclo[3.1.1]hept-2-en-2-yl)methyl)-1,3,4-thiadiazol- 2-yl)-3-(o-tolyl) thiourea*. White solid. Yield: 69.0%, m.p. 178.6–179.4 °C; FT-IR (KBr, cm^−1^): 3325, 3307 (N–H), 3022 (=C–H), 1632 (C=C), 1586, 1500 (Ar–C=C), 1524 (C=N), 1434, 1366, 1081 (N–C=S), 654 (C–S–C); 1H-NMR (500 MHz, DMSO) δ 13.27 (s, 1H, N–H), 9.98 (s, 1H, N–H), 7.32–7.22 (m, 2H, C_16_-H, C_18_-H), 7.21–7.14 (m, 2H, C_19_-H, C_17_-H), 5.48 (s, 1H, C_3_-H), 3.53 (q, *J* = 15.4 Hz, 2H, C_10_-H), 2.35 (dt, *J* = 8.4, 5.6 Hz, 1H, C_1_-H), 2.30–2.15 (m, 5H, C_4_-H_a_, C_7_-H_a_, C_20_-CH_3_), 2.04 (d, *J* = 4.9 Hz, 2H, C_7_-H_b_, C_5_-H), 1.22 (s, 3H, C_9_-CH_3_), 1.10 (d, *J* = 8.4 Hz, 1H, C_4_-H_b_), 0.76 (s, 3H, C_8_-CH_3_); ^13^C-NMR (126 MHz, DMSO) δ 185.1, 170.6, 157.2, 143.8, 138.3, 134.8, 130.7, 128.1, 127.0, 126.5, 120.4, 45.1, 40.4, 38.0, 37.4, 31.9, 31.4, 26.4, 21.4, 18.3; ESI-MS *m*/*z*: 385.28 ([M + H^+^]). Anal. calcd. for C_20_H_24_N_4_S_2_: C, 62.47; H, 6.29; N, 14.57; Found: C, 62.46; H, 6.29; N:14.50.

Compound(**6c**): *1-(5-((6,6-Dimethylbicyclo[3.1.1]hept-2-en-2-yl)methyl)-1,3,4-thiadiazol- 2-yl)-3-(m-tolyl) thiourea*. White solid. Yield: 50.3%, m.p. 182.6–184.6 °C; FT-IR (KBr, cm^−1^): 3330 (N-H), 3035 (=C-H), 1663 (C=C), 1611, 1495 (Ar-C=C), 1555 (C=N), 1438, 1359, 1089 (N-C=S), 660 (C-S-C); ^1^H-NMR (500 MHz, DMSO) δ 13.91 (s, 1H, N-H), 10.33 (s, 1H, N-H), 7.47 (d, *J* = 9.4 Hz, 2H, C_15_-H, C_19_-H), 7.18 (t, *J* = 7.7 Hz, 1H, C_18_-H), 6.90 (d, *J* = 6.6 Hz, 1H, C_17_-H), 5.50 (s, 1H, C_3_-H), 3.58–3.50 (m, 2H, C_10_-H), 2.36 (dt, *J* = 8.4, 5.6 Hz, 1H, C_1_-H), 2.28 (d, *J* = 15.2 Hz, 4H, C_4_-H_a_, C_20_-CH_3_), 2.18 (d, *J* = 17.6 Hz, 1H, C_7_-H_a_), 2.05 (d, *J* = 5.4 Hz, 2H, C_7_-H_b_, C_5_-H), 1.22 (s, 3H, C_9_-CH_3_), 1.11 (d, *J* = 8.5 Hz, 1H, C_4_-H_b_), 0.76 (s, 3H, C_8_-CH_3_); ^13^C-NMR (126 MHz, DMSO) δ 184.6, 170.1, 156.7, 143.6, 140.1, 138.0, 128.6, 125.1, 123.6, 112.8, 120.5, 45.1, 40.4, 38.0, 37.6, 31.9, 31.4, 26.4, 21.6, 21.4; ESI-MS *m*/*z*: 385.29 ([M + H^+^]). Anal. calcd. for C_20_H_24_N_4_S_2_:C, 62.47; H, 6.29; N, 14.57; Found: C, 62.45; H, 6.27; N:14.52.

Compound(**6d**): *1-(5-((6,6-Dimethylbicyclo[3.1.1]hept-2-en-2-yl)methyl)-1,3,4-thiadiazol- 2-yl)-3-(p-tolyl) thiourea***.** White solid. Yield: 61.2%, m.p. 185.5–187.0 °C; FT-IR (KBr, cm^−1^): 3321 (N–H), 3035 (=C-H), 1663 (C=C), 1601, 1512 (Ar–C=C), 1545 (C=N), 1436, 1368, 1081 (N–C=S), 652 (C-S-C); ^1^H-NMR (500 MHz, DMSO) δ 14.01 (s, 1H, N–H), 10.33 (s, 1H, N–H), 7.52 (d, *J* = 8.3 Hz, 2H, C_16_-H, C_18_-H), 7.11 (d, *J* = 8.0 Hz, 2H, C_15_-H, C_19_-H), 5.50 (s, 1H, C_3_-H), 3.57–3.49 (m, 2H, C_10_-H), 2.36 (dt, *J* = 8.5, 5.6 Hz, 1H, C_1_-H), 2.28 (d, *J* = 15.8 Hz, 4H, C_4_-H_a_, C_20_-CH_3_), 2.18 (d, *J* = 17.7 Hz, 1H, C_7_-H_a_), 2.05 (d, *J* = 4.5 Hz, 2H, C_7_-H_b_, C_5_-H), 1.22 (s, 3H, C_9_-CH_3_), 1.10 (d, *J* = 8.5 Hz, 1H, C_4_-H_b_), 0.76 (s, 3H, C_8_-CH_3_); ^13^C-NMR (126 MHz, DMSO) δ 184.6, 170.6, 156.4, 143.6, 137.6, 133.5, 129.2, 123.1, 120.5, 45.1, 40.4, 38.0, 37.6, 31.9, 31.4, 26.4, 21.4, 21.0; ESI-MS *m*/*z*: 385.29 ([M + H^+^]). Anal. calcd. for C_20_H_24_N_4_S_2_: C, 62.47; H, 6.29; N, 14.57; Found: C, 62.42; H, 6.28; N:14.51.

Compound(**6e**): *1-(5-((6,6-Dimethylbicyclo[3.1.1]hept-2-en-2-yl)methyl)-1,3,4-thiadiazol- 2-yl)-3-(3-methoxyphenyl) thiourea*. White solid. Yield: 64.6%, m.p. 152.0–155.0 °C; FT-IR (KBr, cm^−1^): 3323 (N–H), 3030 (=C–H), 1652 (C=C), 1599, 1495 (Ar–C=C), 1535 (C=N), 1434, 1359, 1079 (N–C=S), 652 (C–S–C); ^1^H-NMR (500 MHz, DMSO) δ 14.18 (s, 1H, N-H), 10.37 (s, 1H, N–H), 7.37 (s, 1H, C_15_-H), 7.26 (d, *J* = 8.0 Hz, 1H, C_18_-H), 7.20 (t, *J* = 8.1 Hz, 1H, C_19_-H), 6.66 (d, *J* = 6.5 Hz, 1H, C_17_-H), 5.51 (s, 1H, C_3_-H), 3.74 (s, 3H, C_20_-OCH_3_), 3.58–3.50 (m, 2H, C_10_-H), 2.36 (dt, *J* = 8.5, 5.6 Hz, 1H, C_1_-H), 2.24 (dd, *J* = 49.5, 17.7 Hz, 2H, C_4_-Ha, C_7_-Ha), 2.08–2.03 (m, 2H, C_7_-H_b_, C_5_-H), 1.22 (s, 3H, C_9_-CH_3_), 1.11 (d, *J* = 8.5 Hz, 1H, C_4_-H_b_), 0.76 (s, 3H, C_8_-CH_3_); ^13^C-NMR (126 MHz, DMSO) δ 184.7, 170.3, 159.7, 156.7, 143.6, 141.3, 129.5, 120.6, 114.9, 109.6, 108.5, 55.5, 45.1, 40.4, 38.0, 37.6, 31.9, 31.4, 26.4, 21.4; ESI-MS *m*/*z*: 401.29 ([M + H^+^]). Anal. calcd. for C_20_H_24_N_4_OS_2_:C, 59.97; H, 6.04; N, 13.99; Found: C, 59.92; H, 6.00; N:13.92.

Compound(**6f**): *1-(5-((6,6-Dimethylbicyclo[3.1.1]hept-2-en-2-yl)methyl)-1,3,4-thiadiazol- 2-yl)-3-(4-methoxyphenyl) thiourea*. White solid. Yield: 70.0%, m.p. 185.1–185.8 °C; FT-IR (KBr, cm^−1^): 3319 (N-H), 3024 (=C-H), 1665 (C=C), 1601, 1510 (Ar-C=C), 1553 (C=N), 1438, 1372, 1077 (N-C=S), 652 (C-S-C); ^1^H-NMR (500 MHz, DMSO) δ 13.74 (s, 1H, N-H), 10.27 (s, 1H, N-H), 7.51 (d, *J* = 8.9 Hz, 2H, C_15_-H, C_19_-H), 6.89 (dd, *J* = 8.7, 1.9 Hz, 2H, C_16_-H, C_18_-H), 5.49 (s, 1H, C_3_-H), 3.74 (s, 3H, C_20_-OCH_3_), 3.53 (q, *J* = 15.4 Hz, 2H, C_10_-H), 2.36 (dt, *J* = 8.4, 5.6 Hz, 1H, C_1_-H), 2.23 (dd, *J* = 49.5, 17.7 Hz, 2H, C_4_-H_a_, C_7_-H_a_), 2.05 (d, *J* = 5.1 Hz, 2H, C_7_-H_b_, C_5_-H), 1.22 (s, 3H, C_9_-CH_3_), 1.10 (d, *J* = 8.5 Hz, 1H, C_4_-H_b_), 0.76 (s, 3H, C_8_-CH_3_); ^13^C-NMR (126 MHz, DMSO) δ 180.7, 157.0, 156.6, 143.7, 133.0, 132.7, 126.5, 125.8, 120.5, 114.1, 114.0, 55.7, 45.1, 40.4, 38.0, 37.5, 31.9, 31.4, 26.4, 21.4; ESI-MS *m*/*z*: 401.31 ([M + H^+^]). Anal. calcd. for C_20_H_24_N_4_OS_2_: C, 59.97; H, 6.04; N, 13.99; Found: C, 59.93; H, 6.02; N:13.95.

Compound(**6g**): *1-(5-((6,6-Dimethylbicyclo[3.1.1]hept-2-en-2-yl)methyl)-1,3,4-thiadiazol- 2-yl)-3-(4-fluorophenyl) thiourea*. White solid. Yield: 55.5%, m.p. 192.6–193.6 °C; FT-IR (KBr, cm^−1^): 3323 (N–H), 3012 (=C–H), 1671 (C=C), 1613, 1510 (Ar–C=C), 1561 (C=N), 1436, 1363, 1079 (N–C=S), 652 (C–S–C); ^1^H-NMR (500 MHz, DMSO) δ 14.03 (s, 1H, N–H), 10.42 (s, 1H, N–H), 7.68–7.64 (m, 2H, C_15_-H, C_19_-H), 7.13 (t, *J* = 8.2 Hz, 2H, C_16_-H, C_18_-H), 5.50 (s, 1H, C_3_-H), 3.57–3.50 (m, 2H, C_10_-H), 2.35 (dt, *J* = 10.4, 5.3 Hz, 1H, C_1_-H), 2.23 (dd, *J* = 49.0, 17.7 Hz, 2H, C_4_-H_a_, C_7_-H_a_), 2.04 (d, *J* = 4.4 Hz, 2H, C_7_-H_b_, C_5_-H), 1.21 (s, 3H, C_9_-CH_3_), 1.10 (d, *J* = 8.5 Hz, 1H, C_4_-H_b_), 0.75 (s, 3H, C_8_-CH_3_); ^13^C-NMR (126 MHz, DMSO) δ 184.8, 170.6, 159.0 (d, *J* = 259.56 Hz), 156.7, 143.6, 136.6, 124.9, 120.6, 115.4 (d, *J* = 21.42 Hz), 45.1, 40.4, 38.0, 37.6, 31.9, 31.4, 26.4, 21.4; ESI-MS *m*/*z*: 389.26 ([M + H^+^]). Anal. calcd. for C_19_H_21_FN_4_S_2_: C, 58.74; H, 5.45; N 14.42; Found: C, 58.70; H, 5.43; N:13.41.

Compound(**6h**): *1-(3-Chlorophenyl)-3-(5-((6,6-dimethylbicyclo[3.1.1]hept-2-en-2-yl)meth yl)-1,3,4-thiadiazol-2-yl) thiourea*. White solid. Yield: 64.8%, m.p. 181.3–183.2 °C; FT-IR (KBr, cm^−1^): 3323 (N–H), 3022 (=C–H), 1663 (C=C), 1592, 1485 (Ar–C=C), 1547 (C=N), 1434, 1357, 1077 (N–C=S), 656 (C-S-C); ^1^H-NMR (500 MHz, DMSO) δ 14.35 (s, 1H, N–H), 10.52 (s, 1H, N–H), 7.86 (s, 1H, C_15_-H), 7.69 (d, *J* = 8.0 Hz, 1H, C_18_-H), 7.31 (t, *J* = 8.1 Hz, 1H, C_19_-H), 7.10 (d, *J* = 6.8 Hz, 1H, C_17_-H), 5.52 (s, 1H, C_3_-H), 3.59–3.52 (m, 2H, C_10_-H), 2.37 (dt, *J* = 8.4, 5.6 Hz, 1H, C_1_-H), 2.24 (dd, *J* = 49.7, 17.7 Hz, 2H, C_4_-H_a_, C_7_-H_a_), 2.06 (d, *J* = 4.9 Hz, 2H, C_7_-H_b_, C_5_-H), 1.22 (s, 3H, C_9_-CH_3_), 1.11 (d, *J* = 8.5 Hz, 1H, H_b_-4), 0.76 (s, 3H, H-8); ^13^C-NMR (126 MHz, DMSO) δ 185.4, 170.6, 156.7, 143.5, 141.7, 138.4, 133.2, 130.4, 123.6, 121.8, 120.7, 45.1, 40.4, 38.0, 37.6, 31.9, 31.4, 26.4, 21.4; ESI-MS *m*/*z*: 405.22 ([M + H^+^]). Anal. calcd. for C_19_H_21_ClN_4_S_2_: C, 56.35; H, 5.23; N, 13.83; Found: C, 56.31; H, 5.20; N:13.82.

Compound(**6i**): *1-(4-Chlorophenyl)-3-(5-((6,6-dimethylbicyclo[3.1.1]hept-2-en-2-yl)meth yl)-1,3,4-thiadiazol-2-yl) thiourea*. White solid. Yield: 68.4%, m.p. 193.9–196.3 °C; FT-IR (KBr, cm^−1^): 3319 (N–H), 3037 (=C–H), 1663 (C=C), 1597, 1495 (Ar–C=C), 1549 (C=N), 1436, 1361, 1091 (N–C=S), 652 (C–S–C); ^1^H-NMR (500 MHz, DMSO) δ 14.23 (s, 1H, N–H), 10.50 (s, 1H, N–H), 7.74 (d, *J* = 8.6 Hz, 2H, C_15_-H, C_19_-H), 7.34 (d, *J* = 8.1 Hz, 2H, C_16_-H, C_18_-H), 5.51 (s, 1H, C_3_-H), 3.58–3.51 (m, 2H, C_10_-H), 2.37 (dt, *J* = 8.3, 5.6 Hz, 1H, C_1_-H), 2.24 (dd, *J* = 49.9, 17.7 Hz, 2H, C_4_-H_a_, C_7_-H_a_), 2.05 (d, *J* = 4.7 Hz, 2H, C_7_-H_b_, C_5_-H), 1.22 (s, 3H, C_9_-CH_3_), 1.11 (d, *J* = 8.5 Hz, 1H, C_4_-H_b_), 0.76 (s, 3H, C_8_-H); ^13^C-NMR (126 MHz, DMSO) δ 185.0, 170.6, 156.6, 143.5, 139.2, 128.6, 127.7, 124.1, 120.6, 45.1, 40.4, 38.0, 37.6, 31.9, 31.4, 26.4, 21.4; ESI-MS *m*/*z*: 405.26 ([M + H^+^]). Anal. calcd. for C_19_H_21_ClN_4_S_2_: C, 56.35; H, 5.23; N, 13.83; Found: C, 56.33; H, 5.21; N:13.80.

Compound(**6j**): *1-(3,4-Dichlorophenyl)-3-(5-((6,6-dimethylbicyclo[3.1.1]hept-2-en-2-yl) methyl)-1,3,4-thiadiazol-2-yl) thiourea*. White solid. Yield: 70.0%, m.p. 203.6–205.1 °C; FT-IR (KBr, cm^−1^): 3311 (N–H), 3024 (=C–H), 1663 (C=C), 1588, 1475 (Ar–C=C), 1539 (C=N), 1434, 1355, 1083 (N–C=S), 646 (C–S–C); ^1^H-NMR (500 MHz, DMSO) δ 14.39 (s, 1H, N–H), 10.59 (s, 1H, N–H), 8.09 (s, 1H, C_15_-H), 7.73 (dd, *J* = 8.7, 1.6 Hz, 1H, C_18_-H), 7.52 (d, *J* = 8.8 Hz, 1H, C_19_-H), 5.52 (s, 1H, C_3_-H), 3.60–3.52 (m, 2H, C_10_-H), 2.36 (dt, *J* = 8.4, 5.6 Hz, 1H, C_1_-H), 2.24 (dd, *J* = 49.7, 17.7 Hz, 2H, C_4_-H_a_, C_7_-H_a_), 2.05 (d, *J* = 5.1 Hz, 2H, C_7_-H_b_, C_5_-H), 1.22 (s, 3H, C_9_-CH_3_), 1.11 (d, *J* = 8.5 Hz, 1H, C_4_-H_b_), 0.76 (s, 3H, C_8_-CH_3_); ^13^C-NMR (126 MHz, DMSO) δ 185.4, 170.9, 156.8, 143.5, 140.4, 131.1, 130.6, 125.3, 123.3, 122.2, 120.7, 45.1, 40.3, 38.0, 37.6, 31.9, 31.4, 26.4, 21.4; ESI-MS *m*/*z*: 439.19 ([M + H^+^]). Anal. calcd. for C_19_H_20_Cl_2_N_4_S_2_: C, 51.93; H, 4.59; N, 12.75; Found: C, 51.92; H, 4.56; N:12.75.

Compound(**6k**): *1-(2-Bromophenyl)-3-(5-((6,6-dimethylbicyclo[3.1.1]hept-2-en-2-yl)meth yl)-1,3,4-thiadiazol-2-yl) thiourea*. White solid. Yield: 61.8%, m.p.162.9–164.4 °C; FT-IR (KBr, cm^−1^): 3294 (N–H), 3030 (=C–H), 1640 (C=C), 1578, 1438 (Ar–C=C), 1528 (C=N), 1361, 1330, 1081 (N–C=S), 652 (C–S–C); ^1^H-NMR (500 MHz, CDCl_3_) δ 14.21 (s, 1H, N–H), 9.01 (s, 1H, N–H), 7.88 (d, *J* = 8.0 Hz, 1H, C_16_-H), 7.66 (d, *J* = 8.0 Hz, 1H, C_19_-H), 7.38 (t, *J* = 7.7 Hz, 1H, C_18_-H), 7.20–7.15 (m, 1H, C_17_-H), 5.48 (s, 1H, C_3_-H), 3.65–3.58 (m, 2H, C_10_-H), 2.36–2.32 (m, 1H, C_1_-H), 2.31–2.20 (m, 2H, C_4_-H_a_, C_7_-H_a_), 2.07 (d, *J* = 5.1 Hz, 2H, C_7_-H_b_, C_5_-H), 1.22 (s, 3H, C_9_-CH_3_), 1.16 (d, *J* = 8.7 Hz, 1H, C_4_-H_b_), 0.80 (s, 3H, C_8_-CH_3_); ^13^C- NMR (126 MHz, CDCl_3_) δ 179.6, 165.5, 161.5, 143.1, 136.7, 133.0, 129.0, 128.2, 127.6, 121.1, 120.0, 45.3, 40.4, 38.1, 37.4, 31.9, 31.3, 26.1, 21.1; ESI-MS *m*/*z*: 449.18 ([M + H^+^]). Anal. calcd. for C_19_H_21_BrN_4_S_2_: C, 50.78; H, 4.71; N, 12.47; Found: C, 50.75; H, 4.66; N:12.43.

Compound(**6l**): *1-(3-Bromophenyl)-3-(5-((6,6-dimethylbicyclo[3.1.1]hept-2-en-2-yl)meth yl)-1,3,4-thiadiazol-2-yl) thiourea*. White solid. Yield: 54.2%, m.p.178.1–179.3 °C; FT-IR (KBr, cm^−1^): 3330 (N–H), 3026 (=C–H), 1663 (C=C), 1588, 1481 (Ar–C=C), 1545 (C=N), 1434, 1357, 1081 (N–C=S), 652 (C-S–C); ^1^H-NMR (500 MHz, CDCl_3_) δ 14.49 (s, 1H, N–H), 9.31 (s, 1H, N–H), 7.99 (s, 1H, C_15_-H), 7.79 (d, *J* = 7.4 Hz, 1H, C_17_-H), 7.36 (d, *J* = 7.8 Hz, 1H, C_19_-H), 7.26 (dd, *J* = 10.4, 5.5 Hz, C_18_-H), 5.54 (s, 1H, C_3_-H), 3.71–3.61 (m, 2H, C_10_-H), 2.38 (dt, *J* = 8.7, 5.7 Hz, 1H, C_1_-H), 2.35–2.22 (m, 2H, C_4_-H_a_, C_7_-H_a_), 2.10 (d, *J* = 4.8 Hz, 2H, C_7_-H_b_, C_5_-H), 1.24 (s, 3H, C_9_-CH_3_), 1.19 (d, *J* = 8.7 Hz, 1H, C_4_-H_b_), 0.81 (s, 3H, C_8_-CH_3_); ^13^C-NMR (126 MHz, CDCl_3_) δ 177.2, 164.7, 161.9, 143.1, 139.8, 130.0, 128.7, 126.1, 122.2, 121.6, 121.2, 45.3, 40.4, 38.1, 37.3, 31.9, 31.3, 26.1, 21.1; ESI-MS *m*/*z*: 449.22 ([M + H^+^]). Anal. calcd. for C_19_H_21_BrN_4_S_2_: C, 50.78; H, 4.71; N, 12.47; Found: C, 50.77; H, 4.69; N:12.46.

Compound(**6m**): *1-(4-Bromophenyl)-3-(5-((6,6-dimethylbicyclo[3.1.1]hept-2-en-2-yl)meth yl)-1,3,4-thiadiazol-2-yl) thiourea*. White solid. Yield: 60.4%, m.p. 205.3–206.7 °C; FT-IR (KBr, cm^−1^): 3319 (N–H), 3037 (=C–H), 1663 (C=C), 1603, 1590, 1489 (Ar–C=C), 1547 (C=N), 1436, 1361, 1077 (N–C=S), 656 (C–S–C); ^1^H-NMR (500 MHz, DMSO) δ 14.21 (s, 1H, N–H), 10.49 (s, 1H, N–H), 7.69 (d, *J* = 8.8 Hz, 2H, C_15_-H, C_19_-H), 7.46 (d, *J* = 8.5 Hz, 2H, C_16_-H, C_18_-H), 5.51 (s, 1H, C_3_-H), 3.58–3.50 (m, 2H, C_10_-H), 2.36 (dt, *J* = 8.4, 5.6 Hz, 1H, C_1_-H), 2.23 (dd, *J* = 49.7, 17.7 Hz, 2H, C_4_-H_a_, C_7_-H_a_), 2.06–2.03 (m, 2H, C_7_-H_b_, C_5_-H), 1.22 (s, 3H, C_9_-CH_3_), 1.11 (d, *J* = 8.5 Hz, 1H, C_4_-H_b_), 0.75 (s, 3H, C_8_-CH_3_); ^13^C-NMR (126 MHz, DMSO) δ 184.8, 170.9, 156.6, 143.5, 139.6, 131.5, 124.4, 120.6, 115.8, 45.1, 40.4, 38.0, 37.6, 31.9, 31.4, 26.4, 21.4; ESI-MS *m*/*z*: 449.20 ([M + H^+^]). Anal. calcd. for C_19_H_21_BrN_4_S_2_: C, 50.78; H, 4.71; N, 12.47; Found: C, 50.74; H, 4.68; N:12.42.

Compound(**6n**): *1-(5-((6,6-Dimethylbicyclo[3.1.1]hept-2-en-2-yl)methyl)-1,3,4-thiadiazol- 2-yl)-3-(2-(trifluoromethyl) phenyl) thiourea*. Yellow solid. Yield: 45.5%, m.p. 159.7–161.3 °C; FT-IR (KBr, cm^−1^): 3305 (N-H), 3039 (=C–H), 1634 (C=C), 1609, 1526 (Ar–C=C), 1586 (C=N), 1436, 1357, 1081 (N–C=S), 656 (C-S-C); ^1^H-NMR (500 MHz, DMSO) δ 13.82 (s, 1H, N–H), 10.04 (s, 1H, N–H), 7.74 (d, *J* = 7.8 Hz, 1H, C_16_-H), 7.69 (t, *J* = 7.7 Hz, 1H, C_17_-H), 7.55 (d, *J* = 5.7 Hz, 1H, C_18_-H), 7.49 (t, *J* = 7.6 Hz, 1H, C_19_-H), 5.50–5.48 (m, 1H, C_3_-H), 3.58–3.50 (m, 2H, C_10_-H), 2.36 (dt, *J* = 8.5, 5.6 Hz, 1H, C_1_-H), 2.23 (d, *J* = 32.0 Hz, 2H, C_4_-H_a_, C_7_-H_a_), 2.05–2.03 (m, 2H, C_7_-H_b_, C_5_-H), 1.22 (s, 3H, C_9_-CH_3_), 1.10 (d, *J* = 8.5 Hz, 1H, C_4_-H_b_), 0.76 (s, 3H, C_8_-CH_3_); ^13^C-NMR (126 MHz, DMSO) δ 188.0, 172.0, 143.7, 133.3, 132.2, 127.5 (d, *J* = 63.9 Hz), 126.7 (q, *J* = 31.2, 30.7 Hz),126.4, 126.1 (q, *J* = 4.9 Hz) 125.7, 124.0 (d, *J* = 273.5 Hz), 120.5, 45.1, 40.4, 38.0, 37.4, 31.9, 31.4, 26.4, 21.4; ESI-MS *m*/*z*: 439.29 ([M + H^+^]). Anal. calcd. for C_20_H_21_F_3_N_4_S_2_: C, 54.78; H, 4.83; N, 12.78; Found: C, 54.77; H, 4.80; N:12.76.

Compound(**6o**): *1-(5-((6,6-Dimethylbicyclo[3.1.1]hept-2-en-2-yl)methyl)-1,3,4-thiadiazol- 2-yl)-3-(3-nitrophenyl) thiourea*. Yellow solid. Yield: 68.7%, m.p. 184.8–188.3 °C; FT-IR (KBr, cm^−1^): 3319 (N–H), 3024 (=C–H), 1665 (C=C), 1599, 1533 (Ar–C=C), 1557 (C=N), 1432, 1357, 1079 (N–C=S), 652 (C–S–C); ^1^H-NMR (500 MHz, DMSO) δ 14.25 (s, 1H, N–H), 10.79 (s, 1H, N–H), 8.61 (s, 1H, C_15_-H), 8.21 (d, *J* = 7.3 Hz, 1H, C_17_-H), 7.89 (d, *J* = 7.1 Hz, 1H, C_19_-H), 7.58 (t, *J* = 8.0 Hz, 1H, C_18_-H), 5.51 (s, 1H, C_3_-H), 3.60–3.52 (m, 2H, C_10_-H), 2.38–2.34 (m, 1H, C_1_-H), 2.23 (dd, *J* = 49.4, 17.6 Hz, 2H, C_4_-H_a_, C_7_-H_a_), 2.05 (d, *J* = 4.1 Hz, 2H, C_7_-H_b_, C_5_-H), 1.21 (s, 3H, C_9_-CH_3_), 1.11 (d, *J* = 8.3 Hz, 1H, C_4_-H_b_), 0.75 (s, 3H, C_8_-CH_3_); ^13^C-NMR (126 MHz, DMSO) δ 185.5, 170.1, 156.8, 148.2, 143.4, 141.4, 130.1, 128.4, 120.7, 118.2, 116.5, 45.1, 40.4, 38.0, 37.6, 31.9, 31.4, 26.4, 21.4; ESI-MS *m*/*z*: 416.21 ([M + H^+^]). Anal. calcd. for C_19_H_21_N_5_O_2_S_2_:C, 54.92; H, 5.09; N, 16.85; Found: C, 54.90; H, 5.05; N:16.84.

Compound(**6p**): *1-(5-((6,6-Dimethylbicyclo[3.1.1]hept-2-en-2-yl)methyl)-1,3,4-thiadiazol- 2-yl)-3-(4-nitrophenyl) thiourea*. Yellow solid. Yield: 54.9%, m.p. 212.6–214.3 °C; FT-IR (KBr, cm^−1^): 3315 (N–H), 3030 (=C–H), 1665 (C=C), 1599, 1508 (Ar–C=C), 1564 (C=N), 1434, 1355, 1081 (N–C=S), 646 (C–S–C); ^1^H-NMR (500 MHz, DMSO) δ 14.49 (s, 1H, N–H), 10.93 (s, 1H, N–H), 8.16 (d, *J* = 9.2 Hz, 2H, C_16_-H, C_18_-H), 8.07 (d, *J* = 9.2 Hz, 2H, C_15_-H, C_19_-H), 5.52 (s, 1H, C_3_-H), 3.58 (q, *J* = 15.5 Hz, 2H, C_10_-H), 2.37 (dt, *J* = 8.2, 5.6 Hz, 1H, C_1_-H), 2.24 (dd, *J* = 49.6, 17.7 Hz, 2H, C_4_-H_a_, C_7_-H_a_), 2.06 (d, *J* = 4.9 Hz, 2H, C_7_-H_b_, C_5_-H), 1.22 (s, 3H, C_9_-CH_3_), 1.11 (d, *J* = 8.5 Hz, 1H, C_4_-H_b_), 0.76 (s, 3H, C_8_-CH_3_); ^13^C-NMR (126 MHz, DMSO) δ 185.5, 170.7, 157.1, 146.4, 143.4, 142.1, 124.9, 121.1, 120.8, 45.2, 40.4, 38.0, 37.6, 31.9, 31.4, 26.4, 21.4; ESI-MS *m*/*z*: 416.28 ([M + H^+^]). Anal. calcd. for C_19_H_21_N_5_O_2_S_2_: C, 54.92; H, 5.09; N, 16.85; Found: C, 54.89; H, 5.05; N:16.81.

Compound(**6q**): *1-(5-((6,6-Dimethylbicyclo[3.1.1]hept-2-en-2-yl)methyl)-1,3,4-thiadiazol- 2-yl)-3-isopropylthiourea*. White solid. Yield: 48.9%, m.p. 188.5–190.5 °C; FT-IR (KBr, cm^−1^): 3315 (N–H), 3037 (=C–H), 1636 (C=C), 1535 (C=N), 1434, 1380, 1081 (N–C=S), 652 (C–S–C); ^1^H-NMR (500 MHz, CDCl_3_) δ 14.14 (s, 1H, N-H), 7.54 (s, 1H, N-H), 5.48 (s, 1H, C_3_-H), 4.53 (dq, *J* = 13.1, 6.4 Hz, 1H, C_14_-H), 3.65–3.53 (m, 2H, C_10_-H), 2.38 (dt, *J* = 8.4, 5.6 Hz, 1H, C_1_-H), 2.34–2.20 (m, 2H, C_4_-H_a_, C_7_-H_a_), 2.08 (d, *J* = 4.5 Hz, 2H, C_7_-H_b_, C_5_-H), 1.33 (d, *J* = 6.3 Hz, 6H, C_15_-H, C_16_-H), 1.24 (s, 3H, C_9_-CH_3_), 1.19 (d, *J* = 8.7 Hz, 1H, C_4_-H_b_), 0.80 (s, 3H, C_8_-CH_3_); ^13^C NMR (126 MHz, CDCl_3_) δ 177.4, 164.6, 161.2, 143.4, 120.8, 100,0 47.2, 45.3, 40.5, 38.1, 37.3, 31.9, 31.3, 26.1, 22.1, 21.1; ESI-MS *m*/*z*:337.27([M + H^+^]). Anal. calcd. for C_16_H_24_N_4_S_2_: C, 57.11; H, 7.19; N, 16.65; Found: C, 57.10; H, 7.16; N:16.60.

Compound(**6r**): *1-Butyl-3-(5-((6,6-dimethylbicyclo[3.1.1]hept-2-en-2-yl)methyl)-1,3,4-thi adiazol-2-yl) thiourea*. White solid. Yield: 64.0%, m.p. 152.8–153.8 °C; FT-IR (KBr, cm^−1^):3325 (N-H), 3041 (=C-H), 1636 (C=C), 1537 (C=N), 1436, 1390, 1079 (N-C=S), 652 (C-S-C); ^1^H-NMR (500 MHz, CDCl_3_) δ 14.28 (s, 1H, N–H), 7.61 (s, 1H, N–H), 5.49 (s, 1H, C_3_-H), 3.69 (dd, *J* = 12.1, 6.4 Hz, 2H, C_14_-H), 3.60 (q, *J* = 15.5 Hz, 2H, C_10_-H), 2.38 (dt, *J* = 8.6, 5.6 Hz, 1H, C_1_-H), 2.34–2.22 (m, 2H, C_15_-H), 2.08 (dd, *J* = 11.4, 5.7 Hz, 2H, C_4_-H_a_, C_7_-H_a_), 1.72–1.68 (m, 2H, C_7_-H_b_, C_5_-H), 1.48 (dd, *J* = 14.9, 7.4 Hz, 2H, C_16_-H), 1.24 (s, 3H, C_9_-CH_3_), 1.19 (d, *J* = 8.7 Hz, 1H, C_4_-H_b_), 0.97 (t, *J* = 7.3 Hz, 3H, C_17_-H), 0.81 (s, 3H, C_8_-CH_3_); ^13^C-NMR (126 MHz, CDCl_3_) δ 178.6, 164.7, 161.3, 143.4, 120.8, 45.2, 40.5, 38.1, 37.3, 31.9, 31.3, 30.6, 26.1, 21.1, 20.1, 13.8; ESI-MS *m*/*z*: 351.31([M + H^+^]). Anal. calcd. for C_17_H_26_N_4_S_2_:C, 58.25; H, 7.48; N, 15.98; Found: C, 58.20; H, 7.46; N:15.95.

### 3.7. Antifungal Activity Test

Testing of the primary biological activity was performed in an isolated culture. Under sterile conditions, the tested compound was dissolved in acetone. Sorporl-144 (200 µg/mL) emulsifier was added to dilute the solution to 500 µg/mL. Then, 1 mL solution of the tested compound was poured into a culture plate, followed by the addition of 9 mL PSA culture medium. The final mass concentration of the title compound was 50 µg/mL. A bacterium tray of 5 mm in diameter cut along the external edge of the mycelium was transferred to the flat surface containing the tested compound and arranged in an equilateral triangular style in duplicate. The culture plates were cultivated at 24 ± 1 °C and the extended diameters of the circles of mycelium were measured after 48 h. The relative inhibition of the circle of mycelium compared with the blank assay was calculated by use of the equation:

Relative inhibitory rate (%) = (*CK* − *PT*)/*CK* × 100%

where *CK* is the extended diameter of the circle of mycelium during the blank assay and *PT* is the extended diameter of the circle of mycelium during testing.

### 3.8. 3D-QSAR Analysis

Molecular modeling was performed using SYBYL-X 2.1.1 software (Tripos, Inc., St. Louis, MO, USA). According to a report in the literature [38], the antifungal activity against *A. solani* was expressed in terms of activity factor (ED) by the formula:

ED = log {*I*/[(100 − *I*) × MW]}

where *I* is the percent inhibition at 50 µg/mL and MW was the molecular weight of the tested compounds.

**Figure 6:**
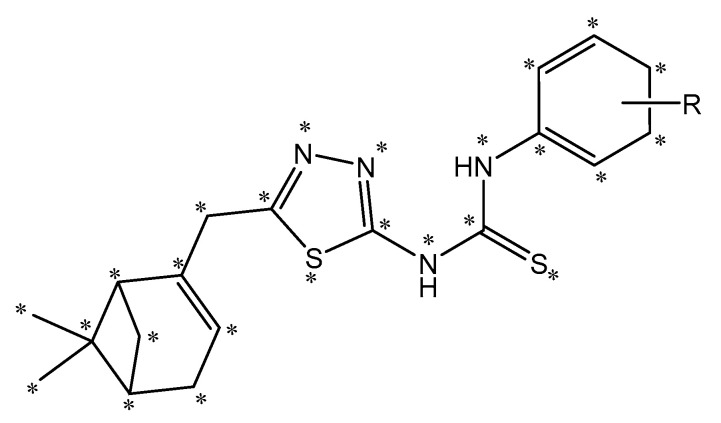
The asterisk skeleton of title compounds.

**Figure 7:**
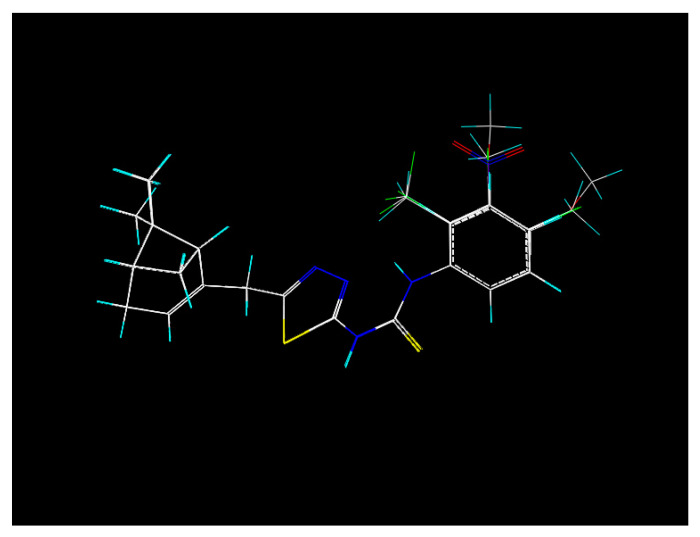
Superposition modes of compounds.

Complete conformational optimization of each structure was performed using a conjugate gradient procedure based on the Tripos force field and Gasteiger–Hückel charges. Compound **6g** (R = *p*-F Ph) was used as a template to build the other molecular structures. According to the common skeleton marked with an asterisk shown in Figure 6, fifteen optimized molecules containing benzene ring were superimposed (Figure 7). The values of the CoMFA field were automatically calculated by the SYBYL/CoMFA routine. A predictive 3D-QSAR model was established using CoMFA descriptors as independent variables and ED values as dependent variables. The cross-validation with the leave-one-out mothed was carried out to obtain the cross-validated *q*^2^ and the optimal number of components. Then, a non-cross-validation analysis under the optimal number of components was performed. The modeling capability was indicated by the correlation coefficient squared *r*^2^, and the prediction capability was judged by the *r*^2^ and *q*^2^.

## 4. Conclusions

Eighteen novel nopol-derived 1,3,4-thiadiazole-thiourea compounds were synthesized, characterized, and evaluated for their antifungal activity. As a result, at 50 µg/mL, all the target compounds exhibited better antifungal activity against *P. piricola*, *C. arachidicola*, and *A. solani*. Compound **6j** (R = *m, p*-Cl Ph) showed the best broad-spectrum antifungal activity against all the tested fungi. Compounds **6c** (R = *m*-Me Ph), **6q** (R = *i*-Pr), and **6i** (R = *p*-Cl Ph) had inhibition rates of 86.1%, 86.1%, and 80.2%, respectively, against *P. piricola*, much better than that of the positive control chlorothalonil. Besides, compounds **6h** (R = *m*-Cl Ph) and **6n** (R = *o*-CF_3_ Ph) held inhibition rates of 80.6% and 79.0% against *C. arachidicola* and *G. zeae*, respectively, much better than that of the commercial fungicide chlorothalonil. Therefore, these new analogs can serve as starting points for additional antifungal studies. In order to design more effective antifungal compounds, a reasonable and effective 3D-QSAR model had been established, and two new molecules with modification on phenyl groups were proposed. Furthermore, some intriguing structure–activity relationships were found and discussed by theoretical calculations.

## Figures and Tables

**Table 1:** Antifungal activity of the target compounds **6a**–**6r** at 50 µg/mL.

Compounds	Inhibition Rate (%) against the Tested Fungi								
	*F. oxysporum* f. sp. *cucumerinum*	*C. arachidicola*	*P. piricola*	*A. solani*	*G. zeae*	*R. solani*	*B. myadis*	*C. orbicalare*	Average (I)
**6a** (R = Ph)	34.1	64.3	64.3	60.6	54.8	19.8	37.7	35.5	46.4
**6b** (R = *o*-Me Ph)	37.8	67.1	75.2	72.3	47.9	19.8	29.1	35.5	48.1
**6c** (R = *m*-Me Ph)	28.5	52.9	86.1	61.3	56.6	17.3	33.4	25.7	45.2
**6d** (R = *p*-Me Ph)	30.4	50.0	66.5	33.1	47.9	53.9	24.9	29.6	42.0
**6e** (R = *m*-OMe Ph)	28.5	64.3	55.7	65.0	58.3	14.9	24.9	27.6	42.4
**6f** (R = *p*-OMe Ph)	21.1	50.0	68.7	50.9	39.3	17.3	22.8	25.7	37.0
**6g** (R = *p*-F Ph)	21.1	70.0	64.3	77.3	56.6	17.3	27.0	27.6	45.2
**6h** (R = *m*-Cl Ph)	30.5	80.6	55.5	69.7	56.8	19.3	36.5	33.6	47.8
**6i** (R = *p*-Cl Ph)	29.8	69.3	80.2	61.3	58.1	22.3	38.4	28.7	48.5
**6j** (R = *m, p*-Cl Ph)	36.1	66.3	66.3	70.3	65.4	21.8	39.7	49.3	51.9
**6k** (R = *o*-Br Ph)	32.2	64.3	75.2	69.0	47.9	14.9	35.5	33.5	46.6
**6l** (R = *m*-Br Ph)	26.7	52.9	51.3	71.0	30.7	19.8	35.5	29.6	39.7
**6m** (R = *p*-Br Ph)	34.1	67.1	64.3	61.3	27.2	17.3	29.1	31.6	41.5
**6n** (R = *o*-CF_3_ Ph)	34.1	61.4	57.8	72.8	79.0	16.1	31.3	37.5	48.8
**6o** (R = *m*-NO_2_ Ph)	26.7	72.9	53.5	70.9	56.6	17.3	29.1	25.7	44.1
**6p** (R = *p*-NO_2_ Ph)	35.9	72.9	36.1	63.8	51.4	17.3	35.5	37.5	43.8
**6q** (R = *i*-Pr)	34.1	70.0	86.1	60.6	58.3	17.3	22.8	29.6	47.4
**6r** (R = *n*-Bu)	28.5	61.4	75.2	63.8	51.4	14.9	24.9	27.6	43.5
Average (II)	30.6	64.3	65.7	64.2	52.5	19.9	31.0	31.7	-
Chlorothalonil	100	73.3	75.0	73.9	73.1	96.1	90.4	91.3	-

**Table 2:** The ED values of experimental and predicted activities.

Compounds	MW	ED	ED^[a]^	Residue
**6a**	370.13	−2.381	−2.368	−0.013
**6b**	384.14	−2.168	−2.197	0.029
**6c**	384.14	−2.385	−2.401	0.016
**6d**	384.14	−2.890	−2.907	0.017
**6e**	400.14	−2.333	−2.317	−0.016
**6f**	400.14	−2.587	−2.578	−0.009
**6g**	388.12	−2.057	−2.079	0.022
**6h**	404.09	−2.245	−2.246	0.001
**6i**	404.09	−2.407	−2.400	−0.007
**6j**	438.05	−2.267	−2.282	0.015
**6k**	448.04	−2.304	−2.264	−0.040
**6l**	448.04	−2.262	−2.260	−0.002
**6m**	448.04	−2.452	−2.443	−0.009
**6n**	438.12	−2.214	−2.225	0.011
**6o**	415.11	−2.231	−2.218	−0.013
**6p** *	415.11	−2.372	−2.360	−0.012

ED: experimental value; ED^[a]^: predictive value; *: test-set compound.

**Table 3:** Summary of CoMFA analysis.

					Contribution (%)	
	*q* ^2^	*r* ^2^	*S*	*F*	*Steric*	*Electrostatic*
CoMFA	0.753	0.992	0.021	294.200	52.9	47.1

*q*^2^: cross-validated correlational coefficient; *r*^2^: non-validated correlational coefficient; *S*: standard error of estimate; *F*: the Fischer ratio.

## Data Availability

Data is contained within the article or the supplementary data file.

## Supplementary Materials

Supplementary materials are available online. Figures S1–S85: FT-IR, ^1^H-NMR, ^13^C-NMR, and ESI-MS spectra of compounds **2**, **3**, **4**, **5**, and **6a**–**6r**.

## Sample Availability

Samples of the compounds are available from the authors.
