# Supplementary material for: Synthesis, Antifungal Activity, and 3D-QSAR Study of Novel Nopol-Derived 1,3,4-Thiadiazole-Thiourea Compounds
Source: Molecules. 2021 Mar 18;26(6):1708. doi: 10.3390/molecules26061708 (PMC8003325; doi:10.3390/molecules26061708)
Supplement: Supplementary file 1 [file molecules-26-01708-s001.zip › molecules-1142334-supplementary.pdf]

Synthesis, Antifungal Activity and 3D-QSAR Study of Novel Nopol-Derived 1,3,4-Thiadiazole-Thiourea Compounds

Ming Chen, Wen-Gui Duan*, Gui-Shan Lin*, Zhong-Tian Fan, Xiu Wang

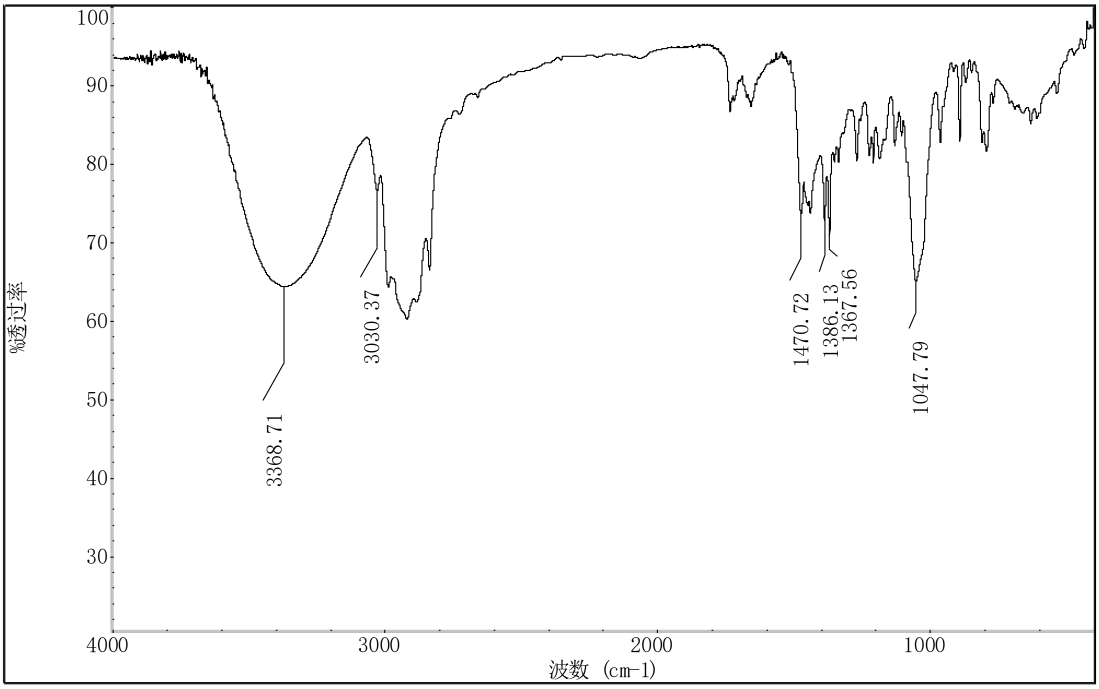

Figure S1. FT-IR spectrum of nopol **2**.

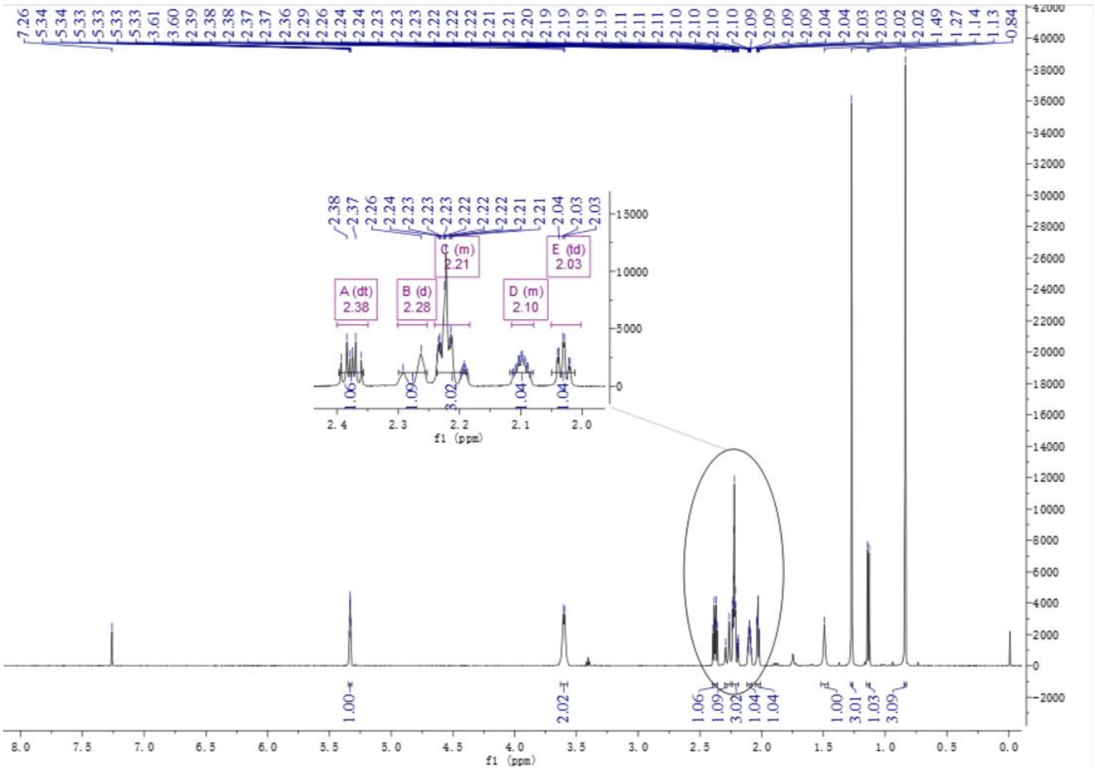

Figure S2. ^{1}H-NMR spectrum of nopol **2** in $CDCl_3$.

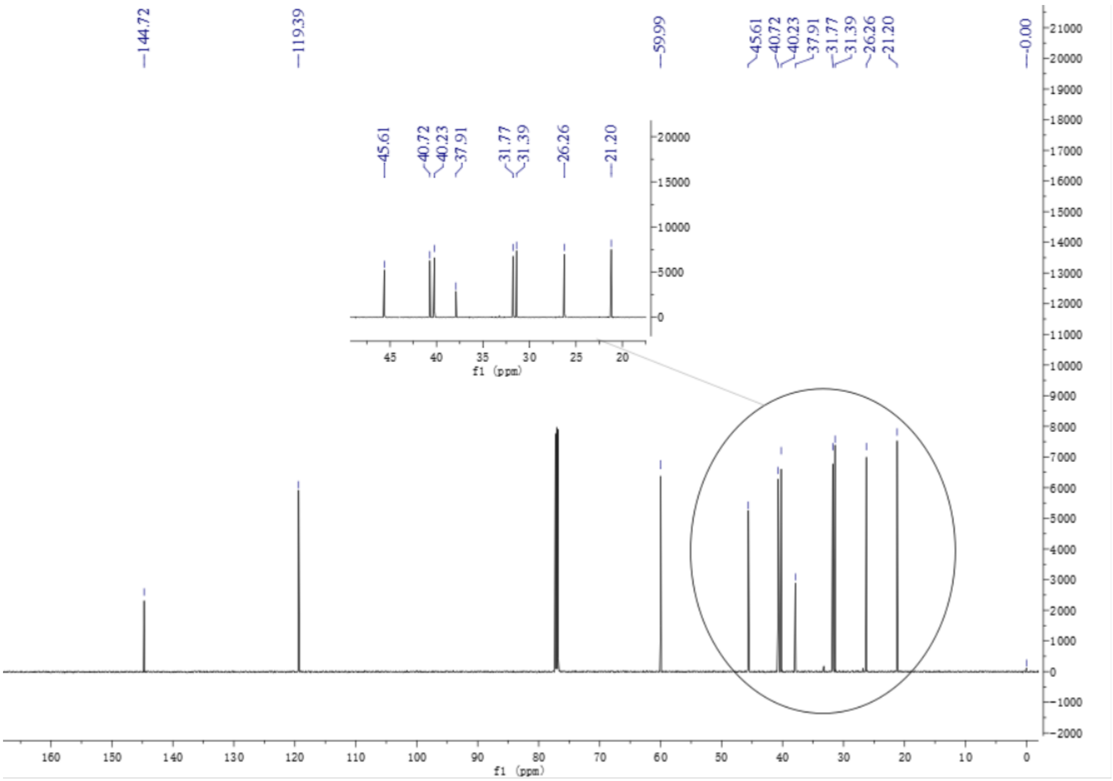

Figure S3. ^{13}C-NMR spectrum of nopol **2** in $CDCl_3$.

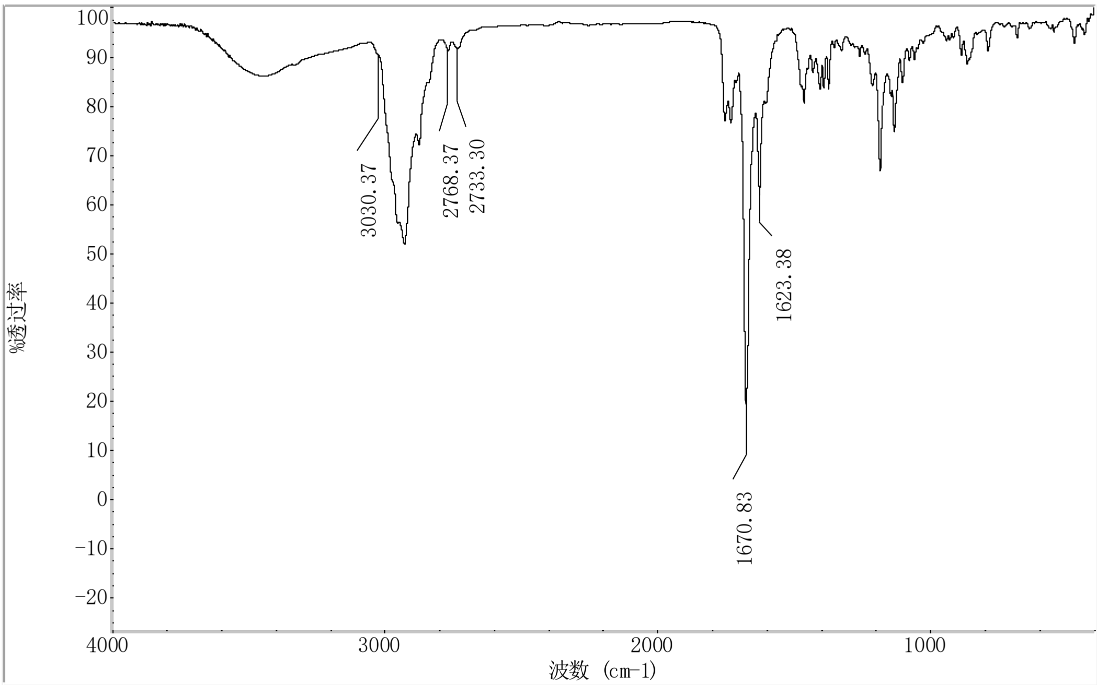

Figure S4. FT-IR spectrum of nopol aldehyde **3**.

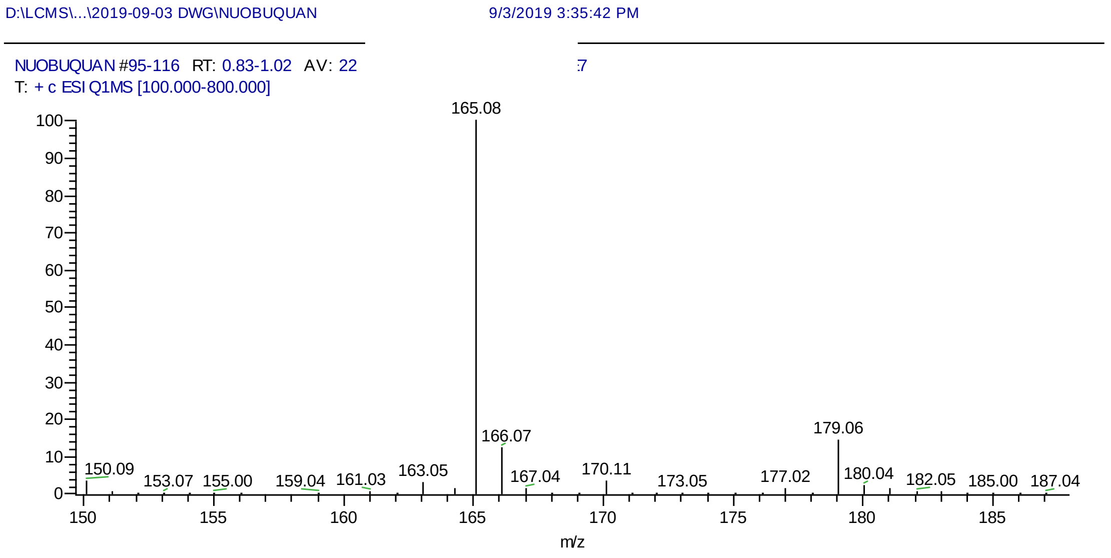

Figure S5. ESI-MS spectrum of nopol aldehyde **3**.

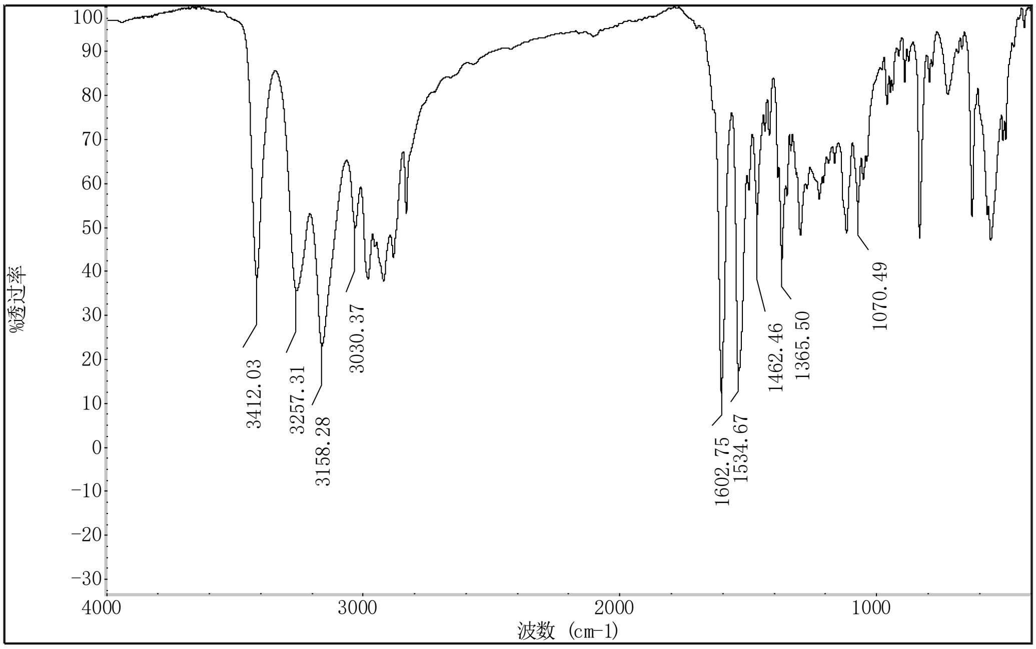

Figure S6. FT-IR spectrum of nopol-based thiosemicarbazone **4**.

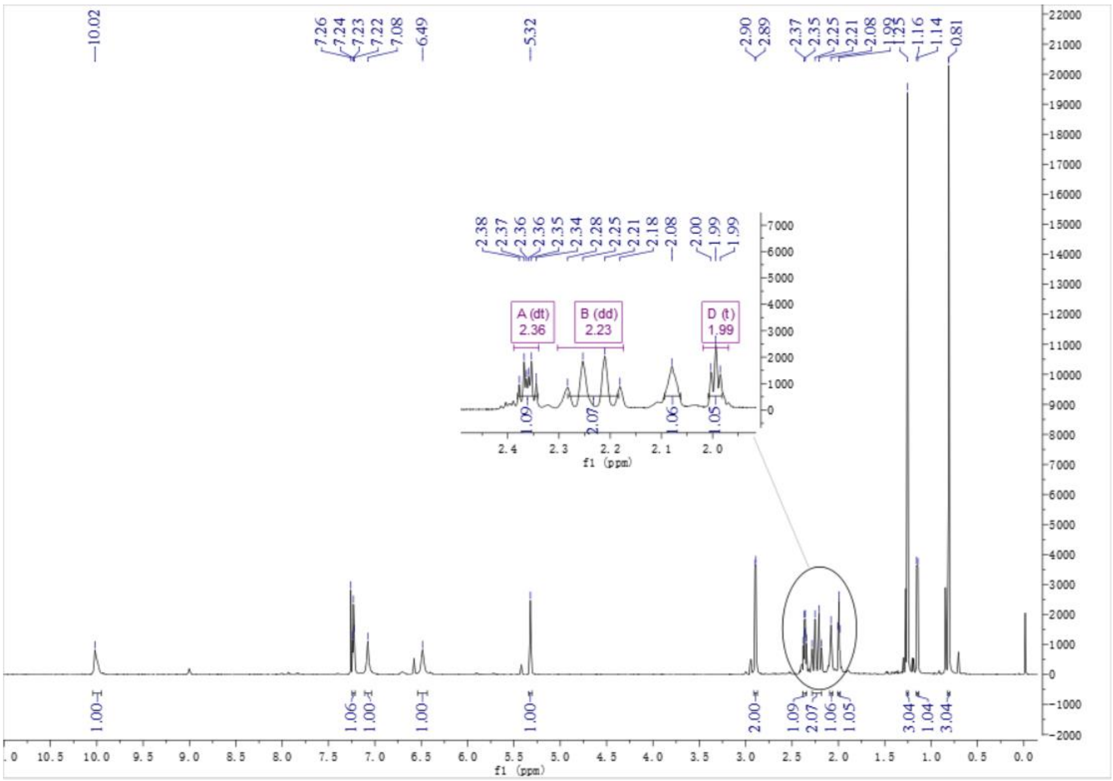

Figure S7. ^{1}H-NMR spectrum of nopol-based thiosemicarbazone **4** in $CDCl_3$.

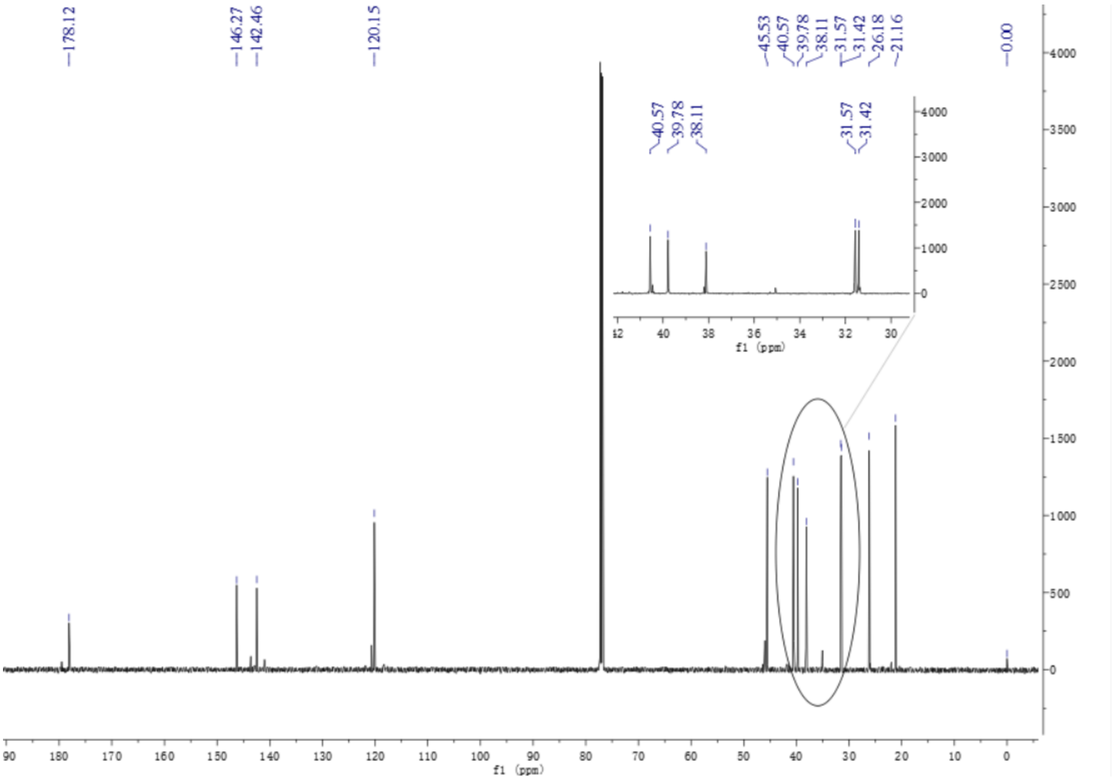

Figure S8. ^{13}C-NMR spectrum of nopol-based thiosemicarbazone **4** in $CDCl_3$.

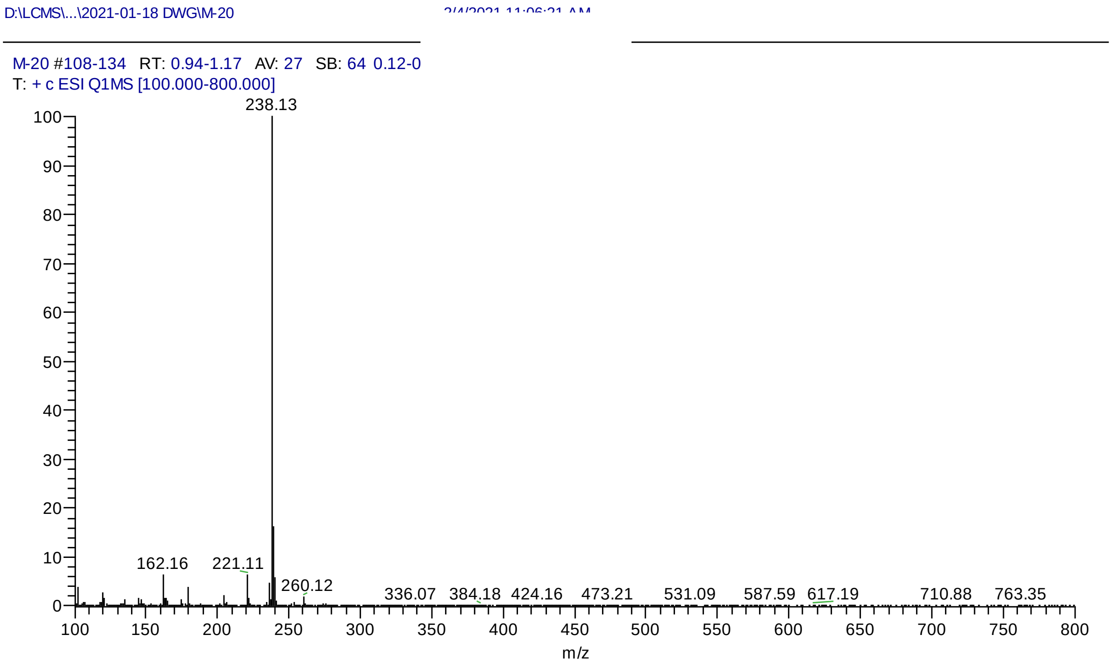

Figure S9. ESI-MS spectrum of nopol-based thiosemicarbazone **4**.

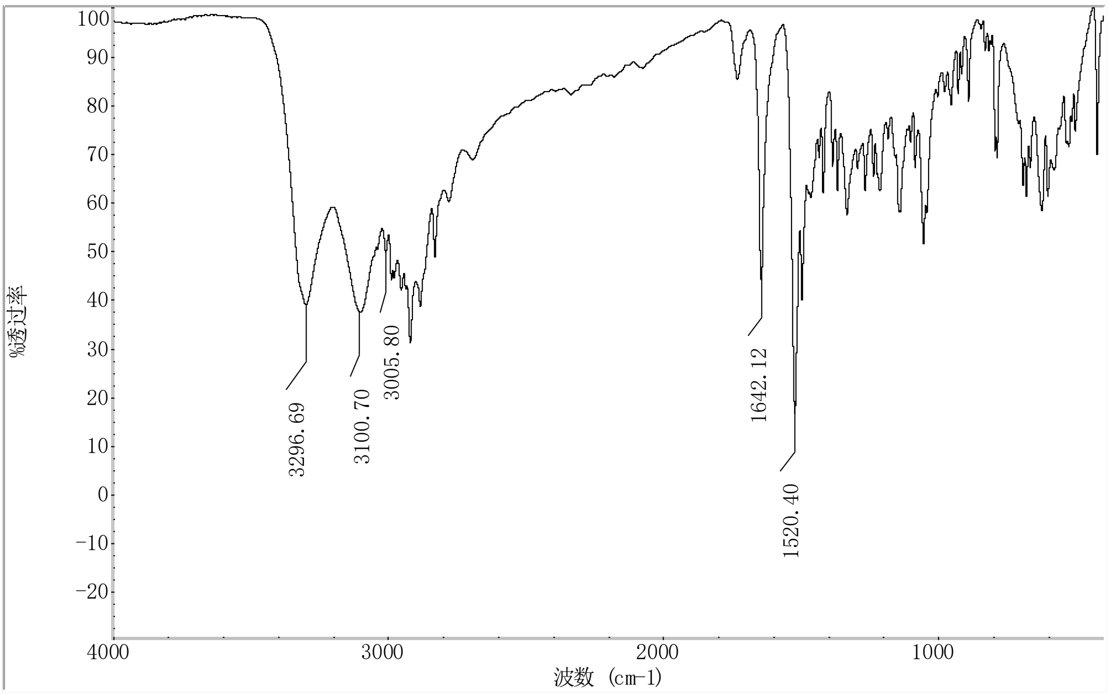

Figure S10. FT-IR spectrum of 5-nopyl-2-amino-1,3,4-thiadiazole **5**.

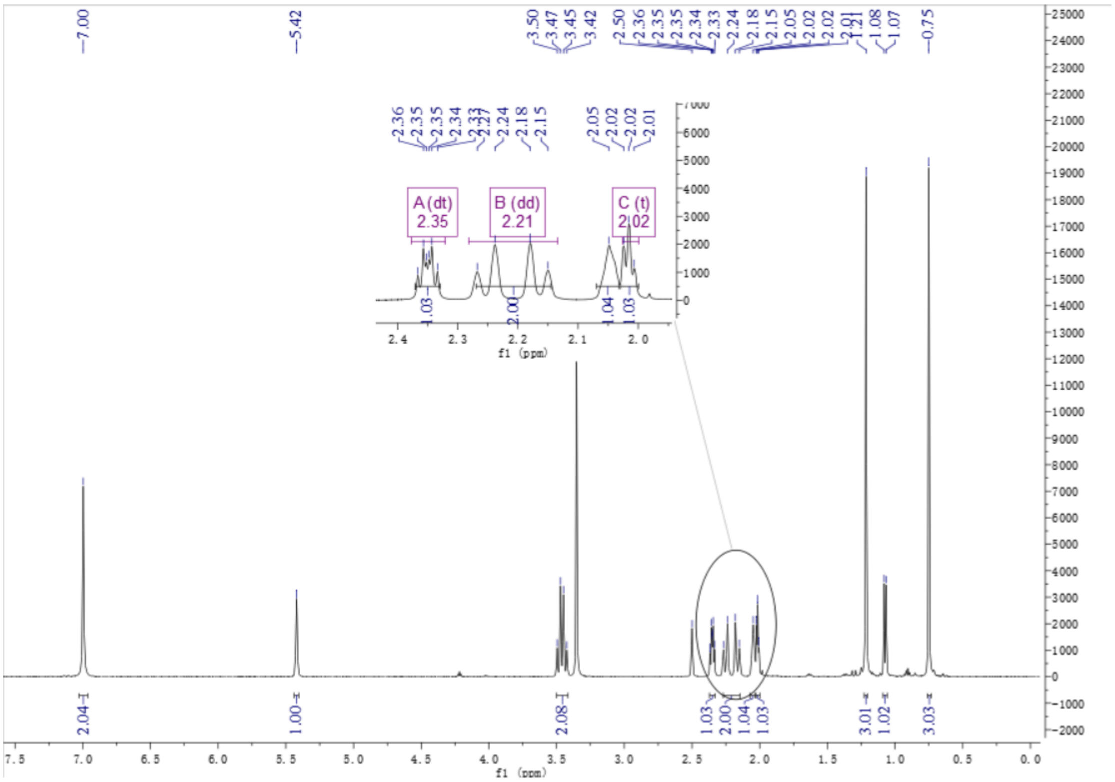

Figure S11. ^{1}H-NMR spectrum of 5-nopyl-2-amino-1,3,4-thiadiazole **5** in DMSO.

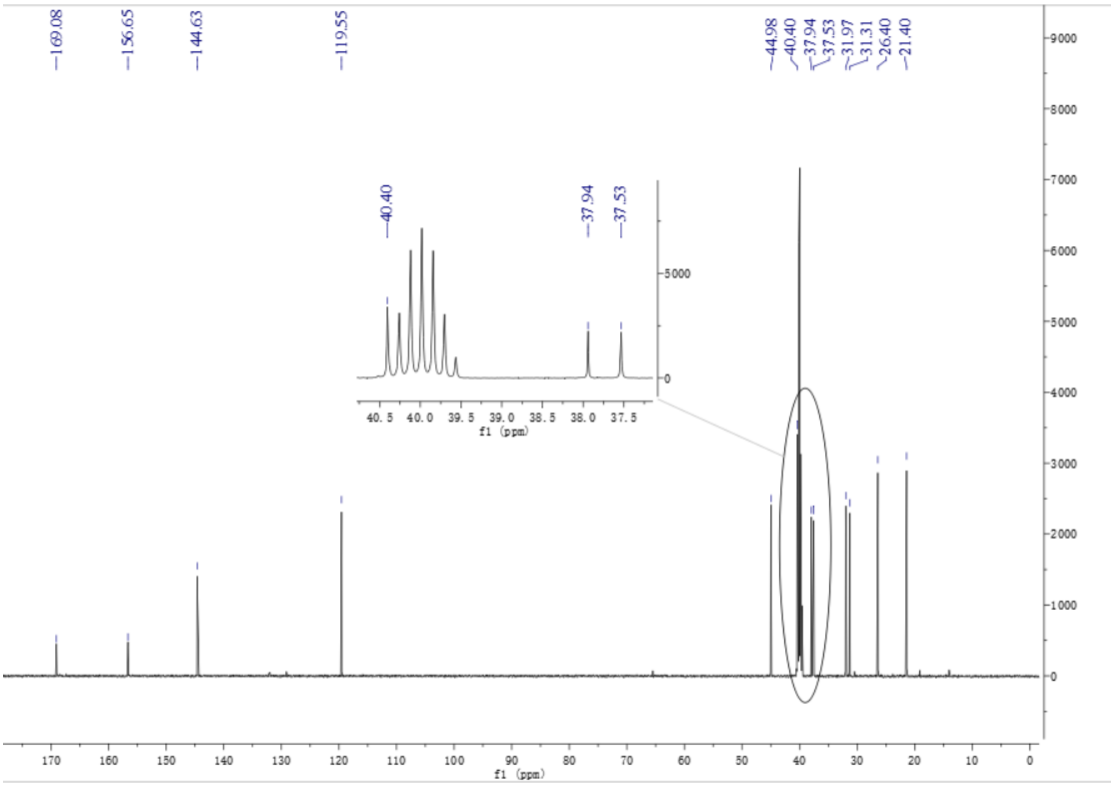

Figure S12. ^{13}C-NMR spectrum of 5-nopyl-2-amino-1,3,4-thiadiazole **5** in DMSO.

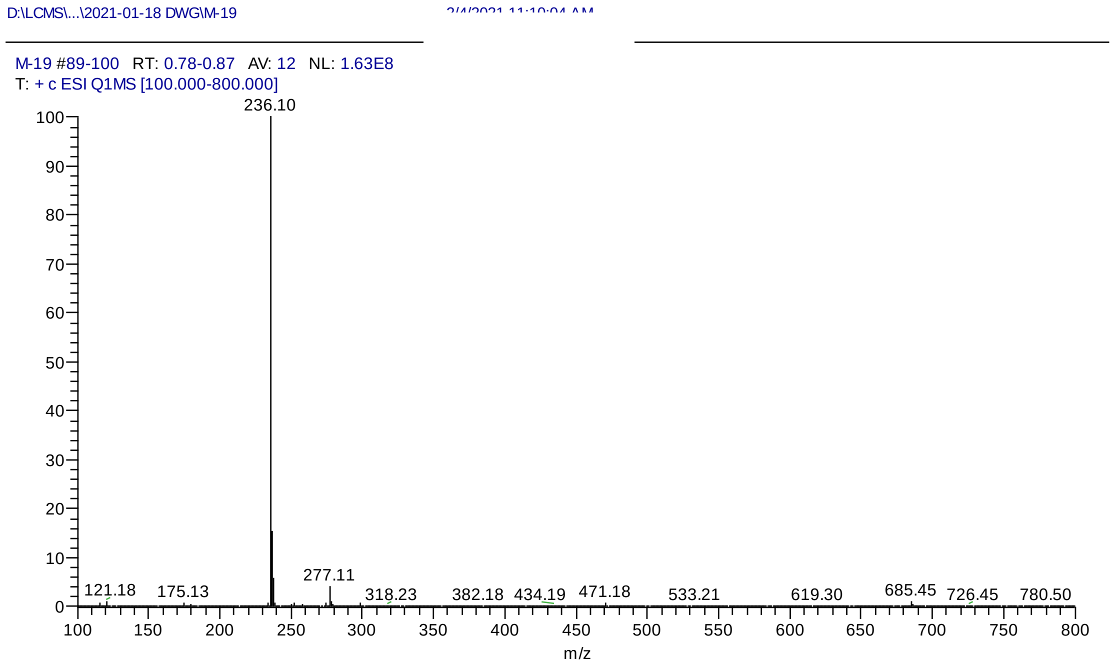

Figure S13. ESI-MS spectrum of 5-nopyl-2-amino-1,3,4-thiadiazole **5**.

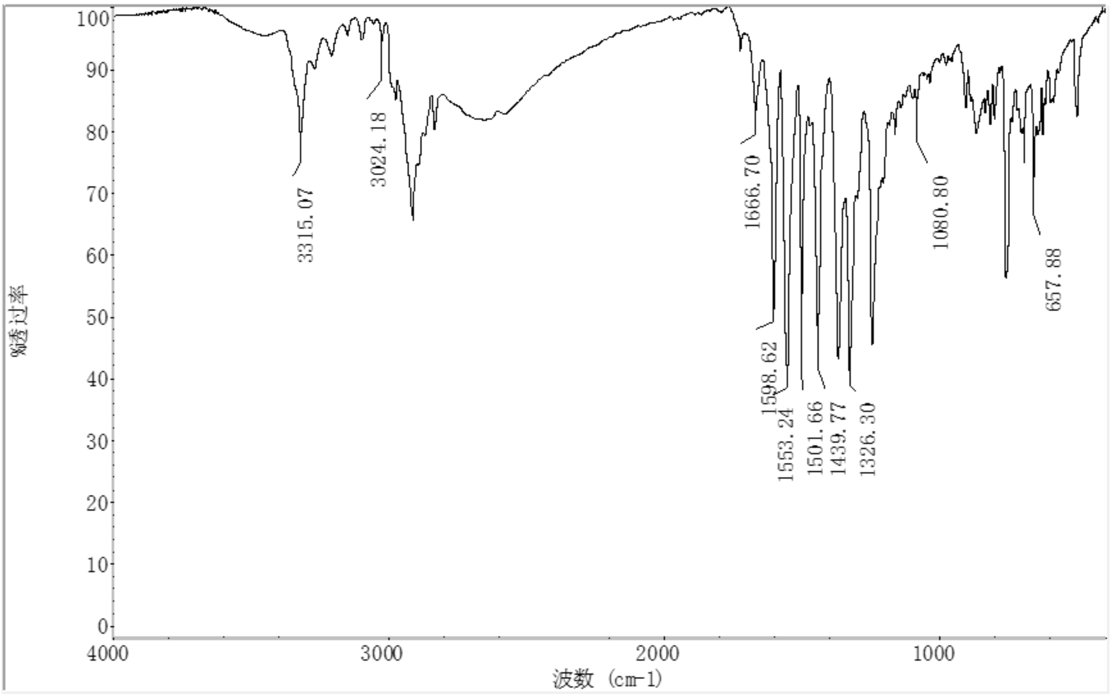

Figure S14. FT-IR spectrum of Compound **6a**(R=Ph).

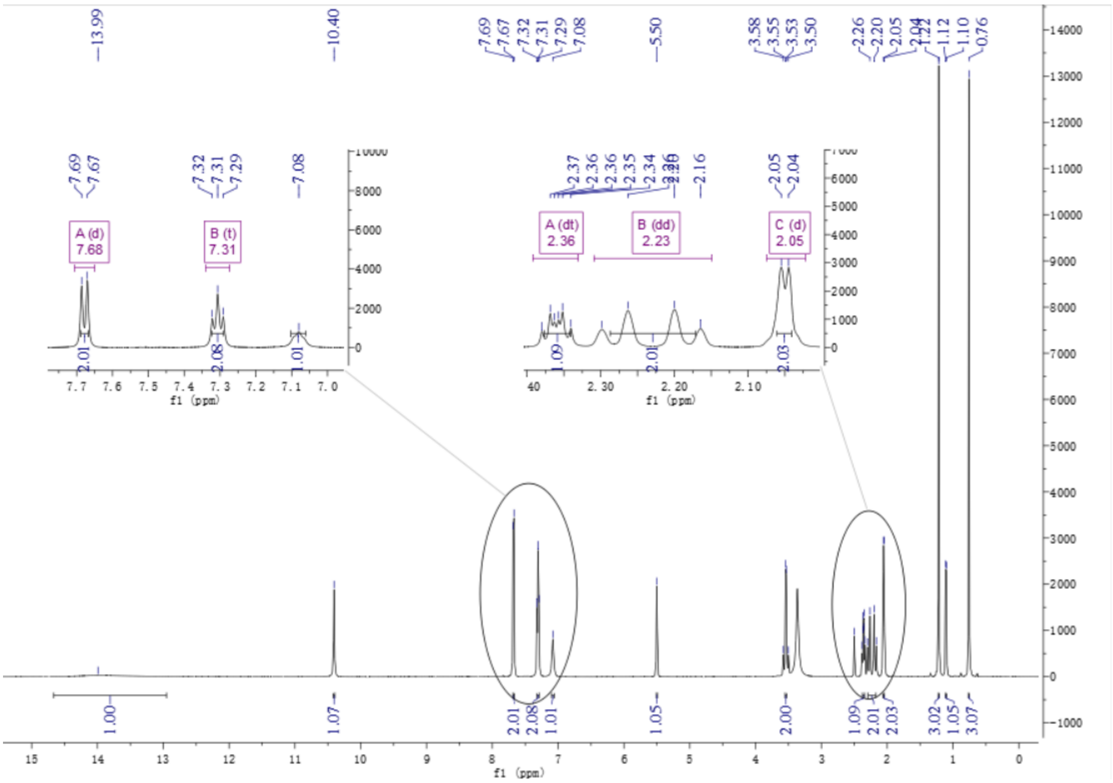

Figure S15. ^{1}H-NMR spectrum of Compound **6a**(R=Ph) in DMSO.

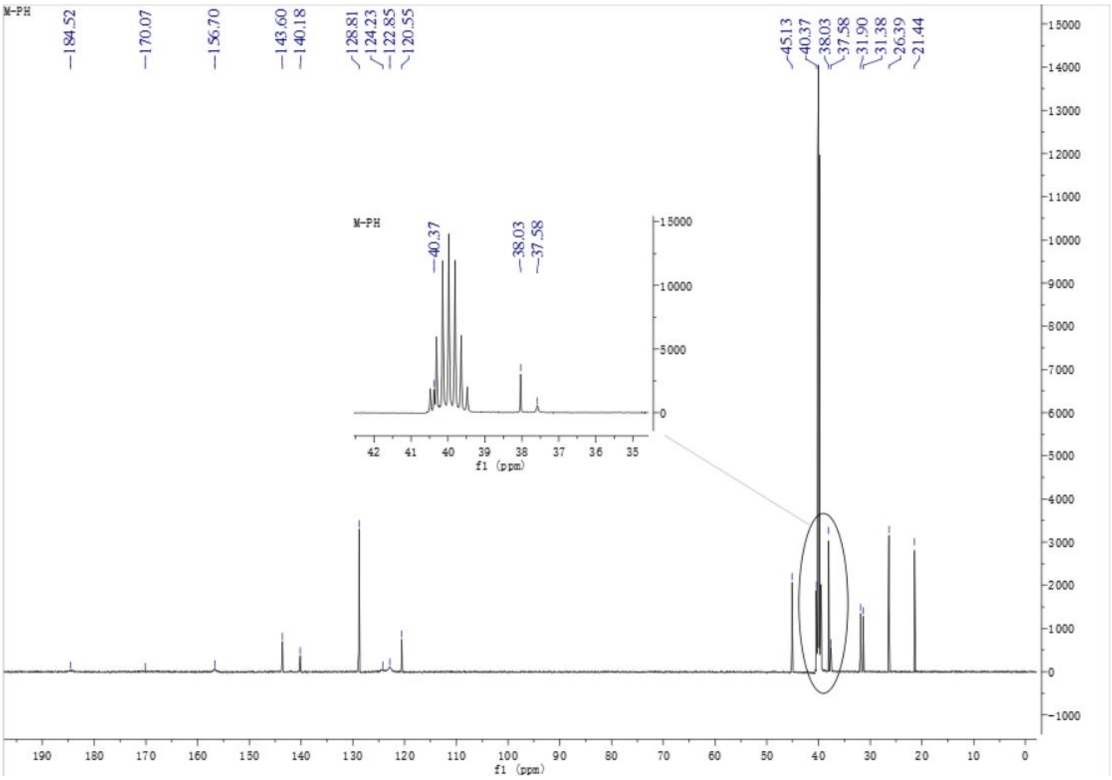

Figure S16. ^{13}C-NMR spectrum of Compound **6a**(R=Ph) in DMSO.

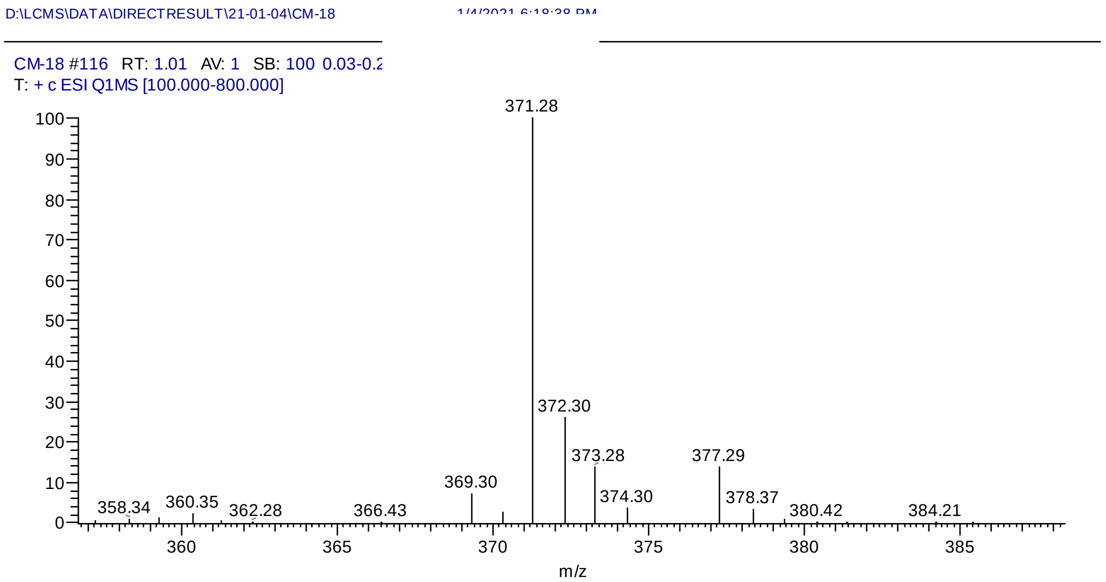

Figure S17. ESI-MS spectrum of Compound **6a**(R=Ph).

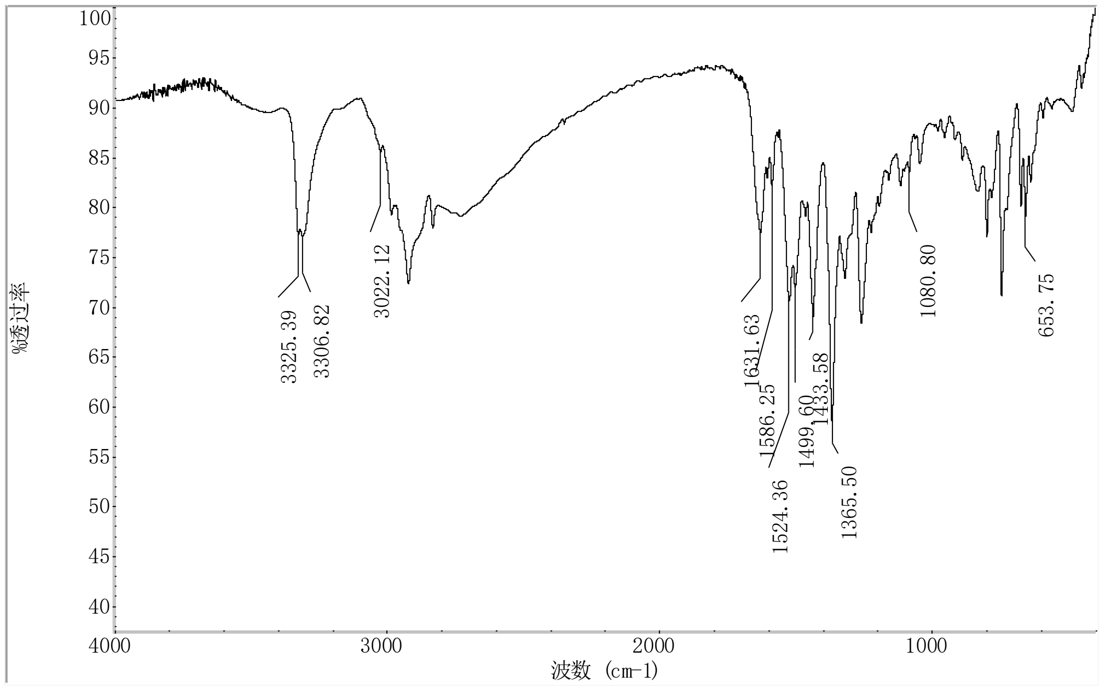

Figure S18. FT-IR spectrum of Compound **6b** (R=*o*-Me Ph).

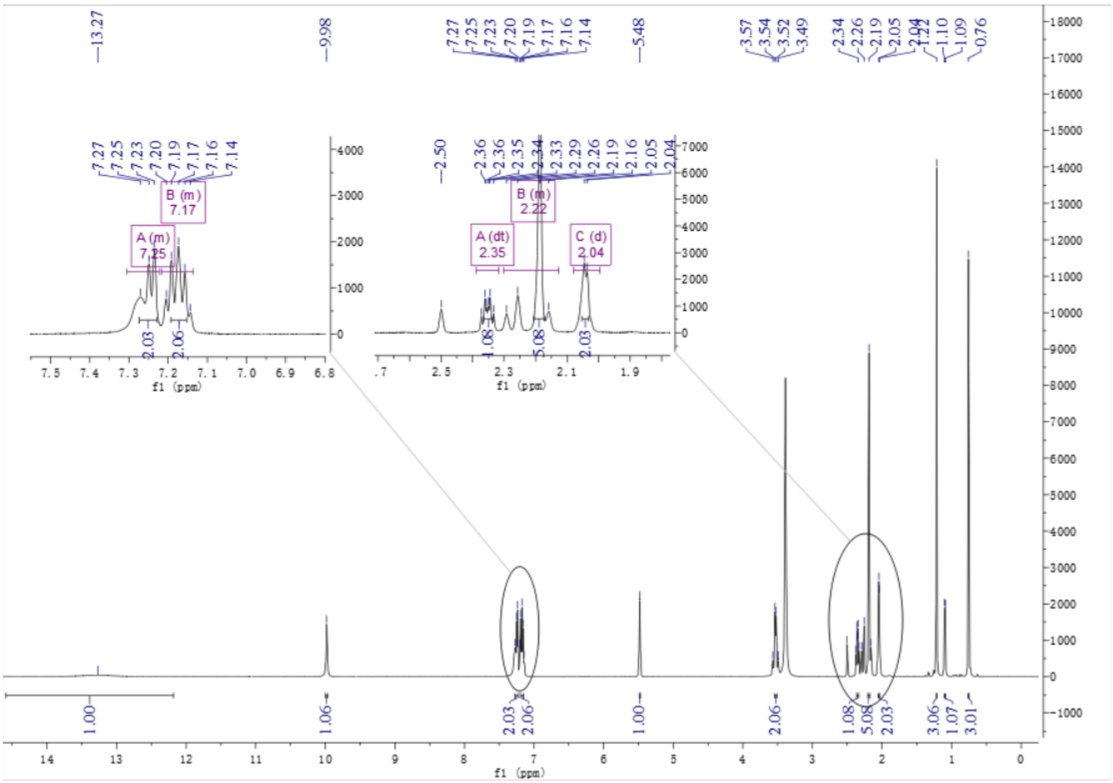

Figure S19. ^{1}H-NMR spectrum of Compound **6b** (R=*o*-Me Ph) in DMSO.

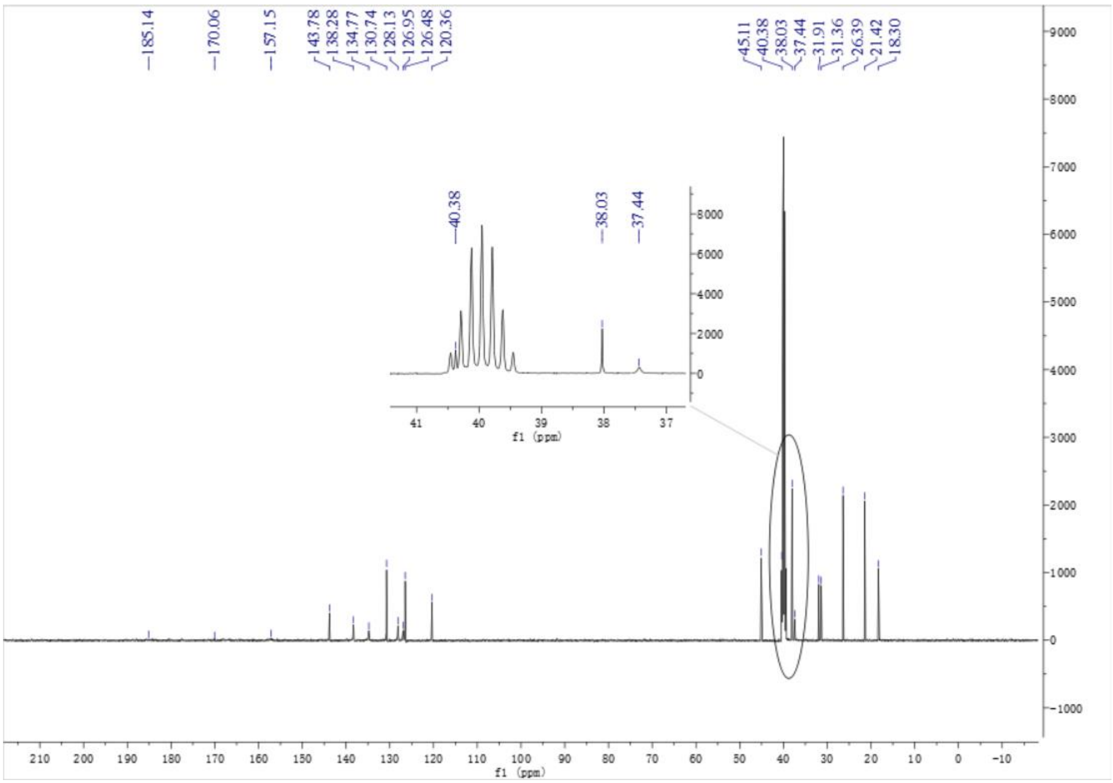

Figure S20. ^{13}C-NMR spectrum of Compound **6b** (R=*o*-Me Ph) in DMSO.

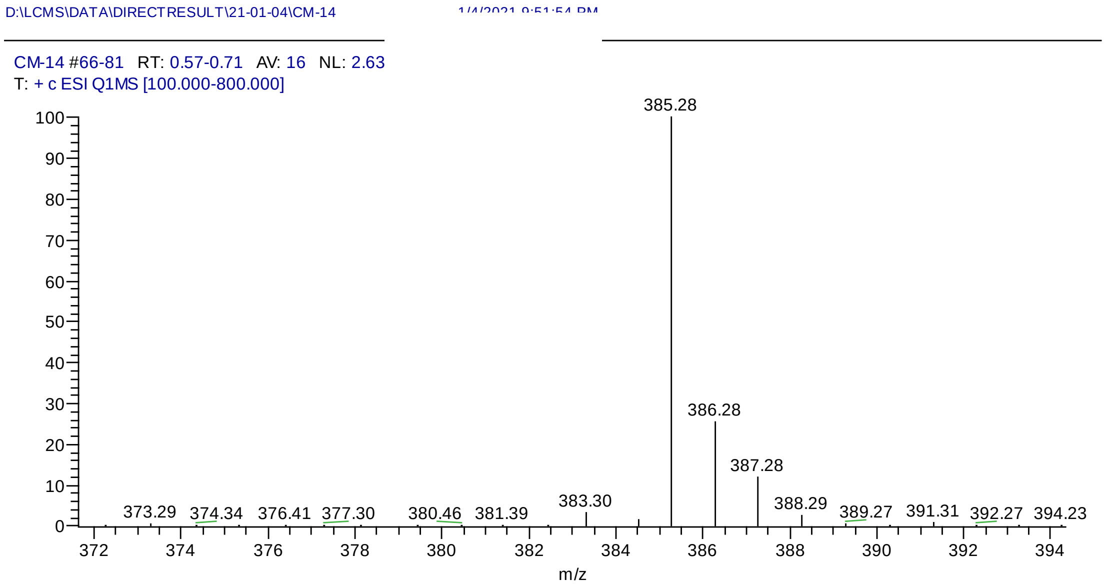

Figure S21. ESI-MS spectrum of Compound **6b** (R=*o*-Me Ph).

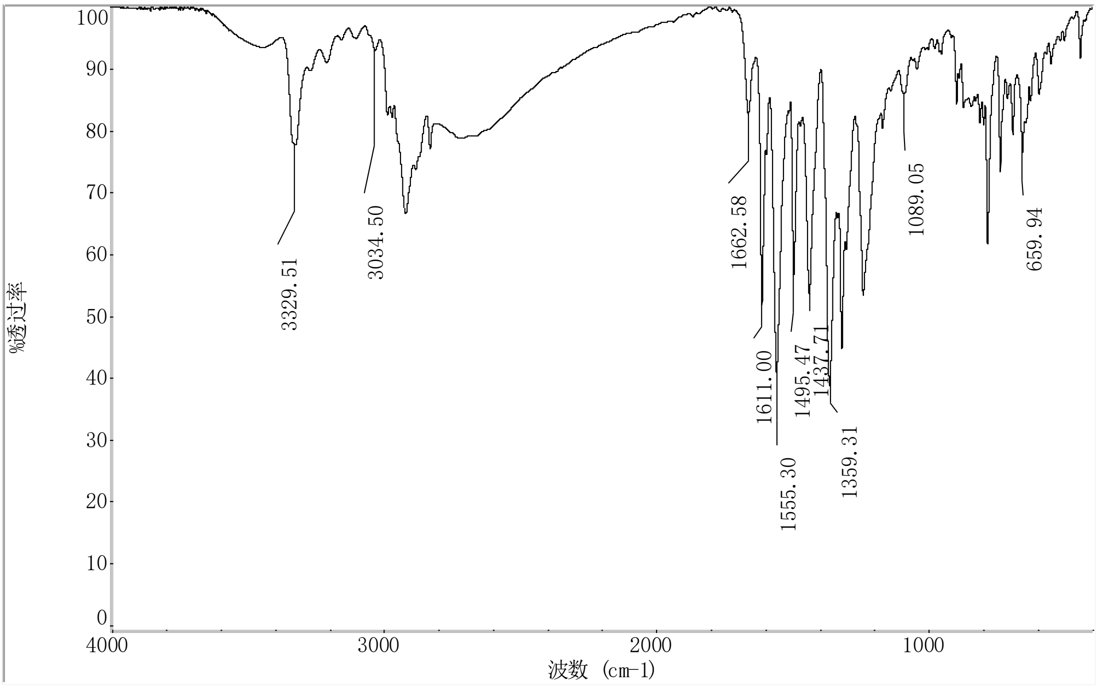

Figure S22. FT-IR spectrum of Compound **6c** (R=*m*-Me Ph).

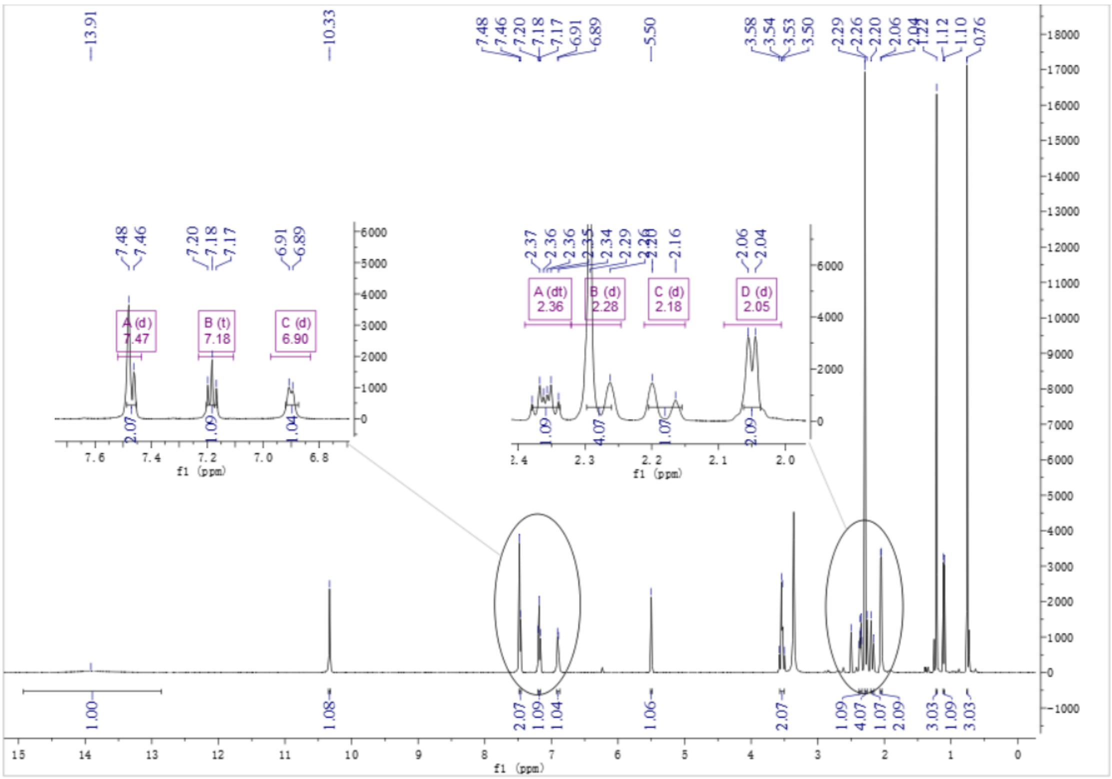

Figure S23. ^{1}H-NMR spectrum of Compound **6c** (R=*m*-Me Ph) in DMSO.

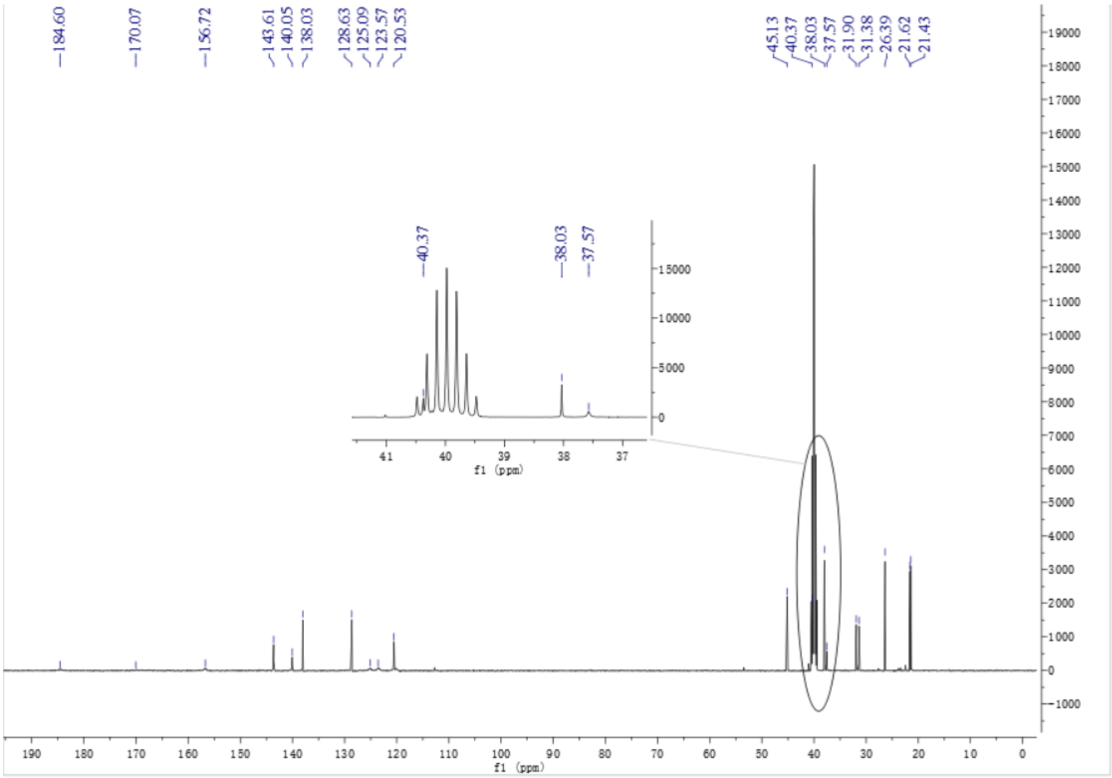

Figure S24. ^{13}C-NMR spectrum of Compound **6c** (R=*m*-Me Ph) in DMSO.

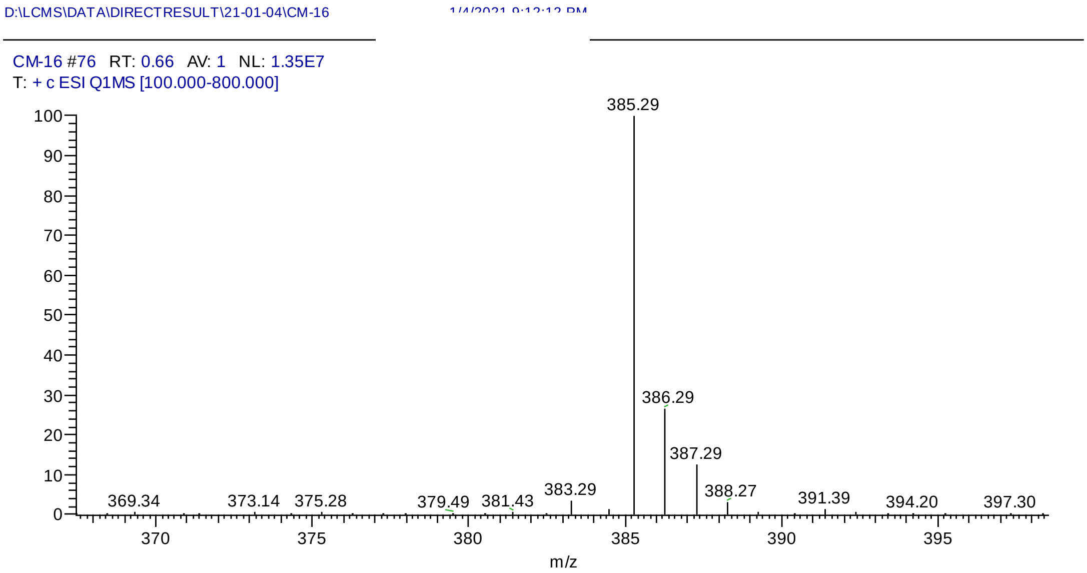

Figure S25. ESI-MS spectrum of Compound **6c** (R=*m*-Me Ph).

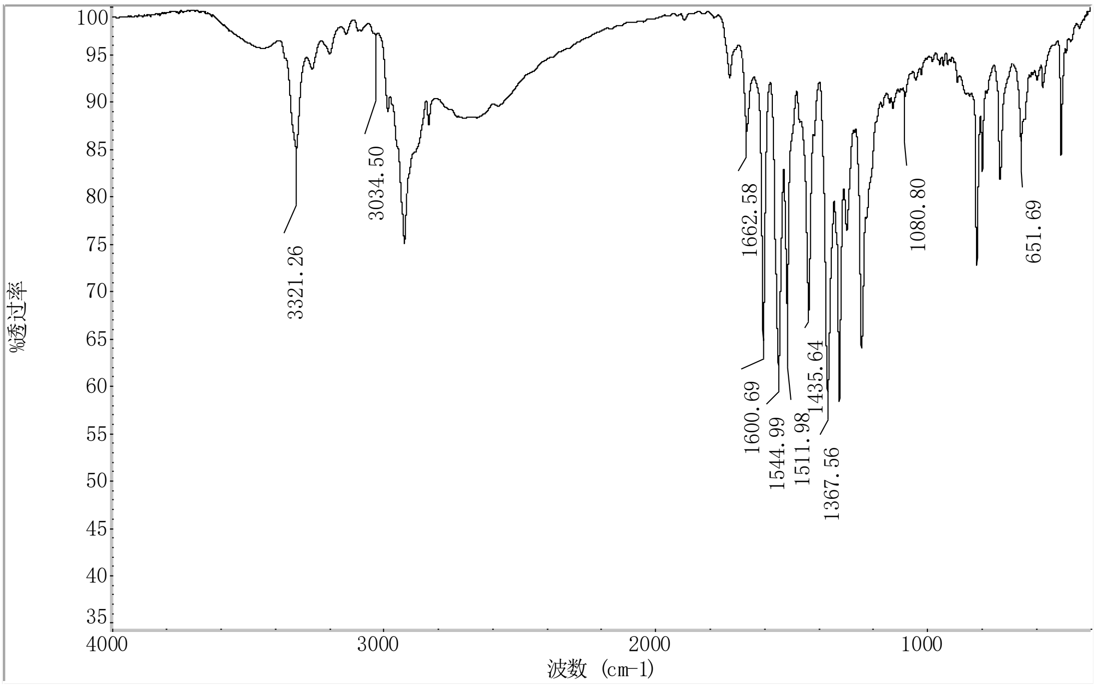

Figure S26. FT-IR spectrum of Compound **6d** (R=*p*-Me Ph).

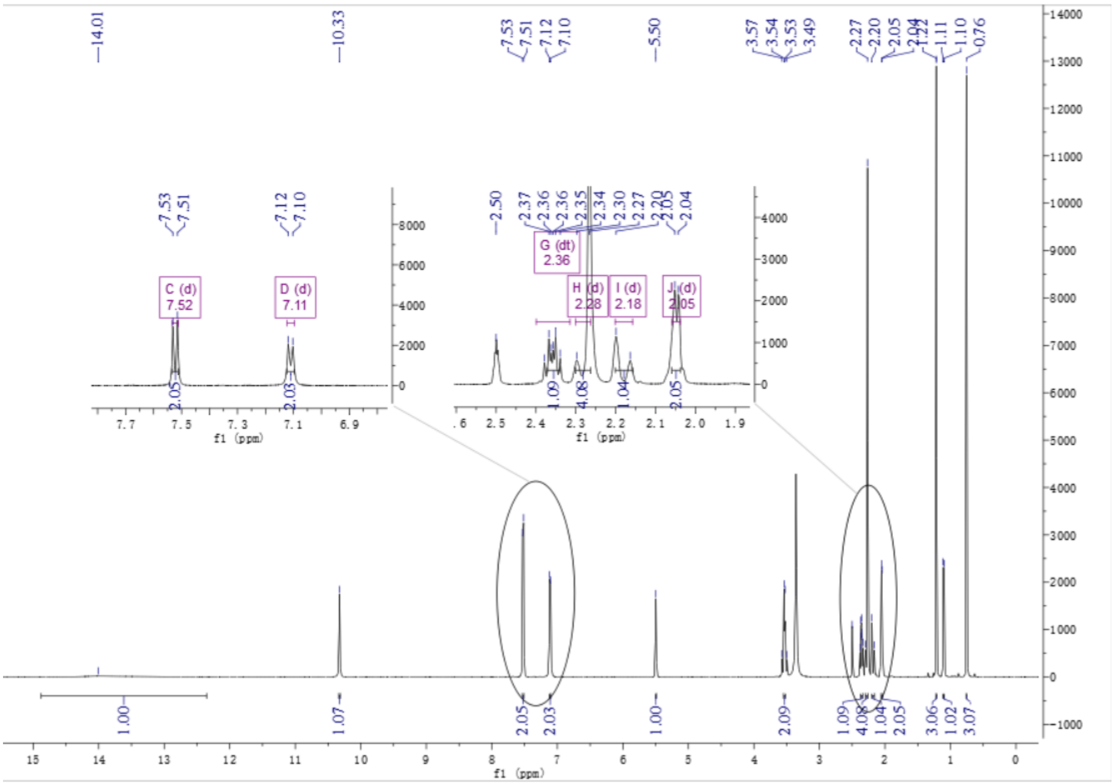

Figure S27. ^{1}H-NMR spectrum of Compound **6d** (R=*p*-Me Ph) in DMSO.

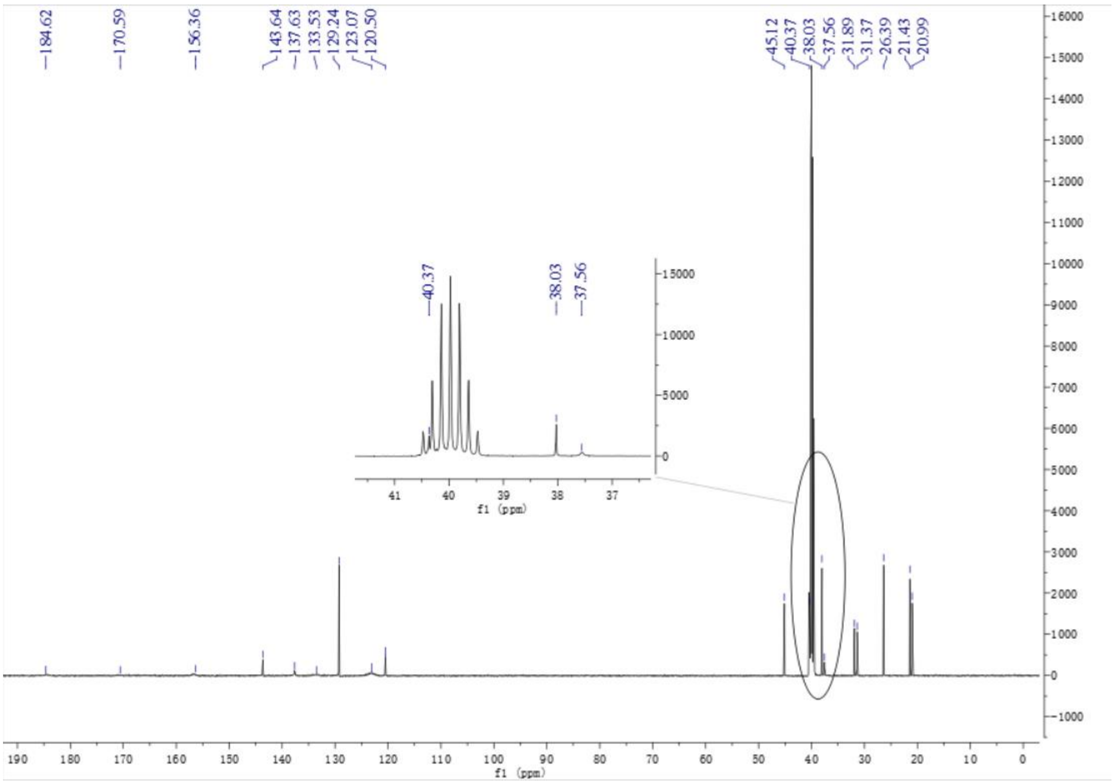

Figure S28. ^{13}C-NMR spectrum of Compound **6d** (R=*p*-Me Ph) in DMSO.

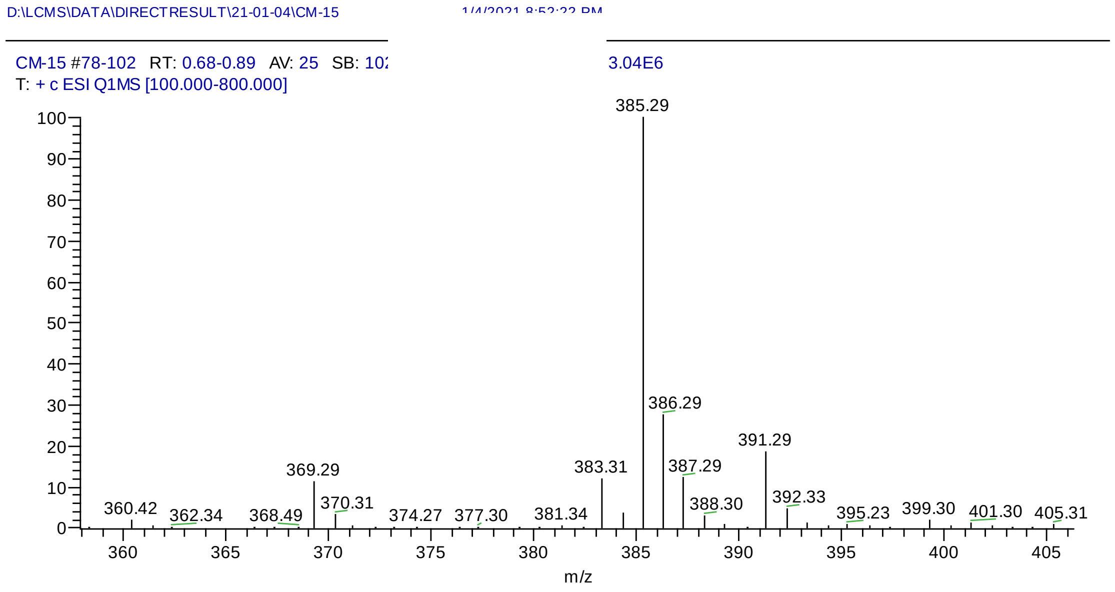

Figure S29. ESI-MS spectrum of Compound **6d** (R=*p*-Me Ph).

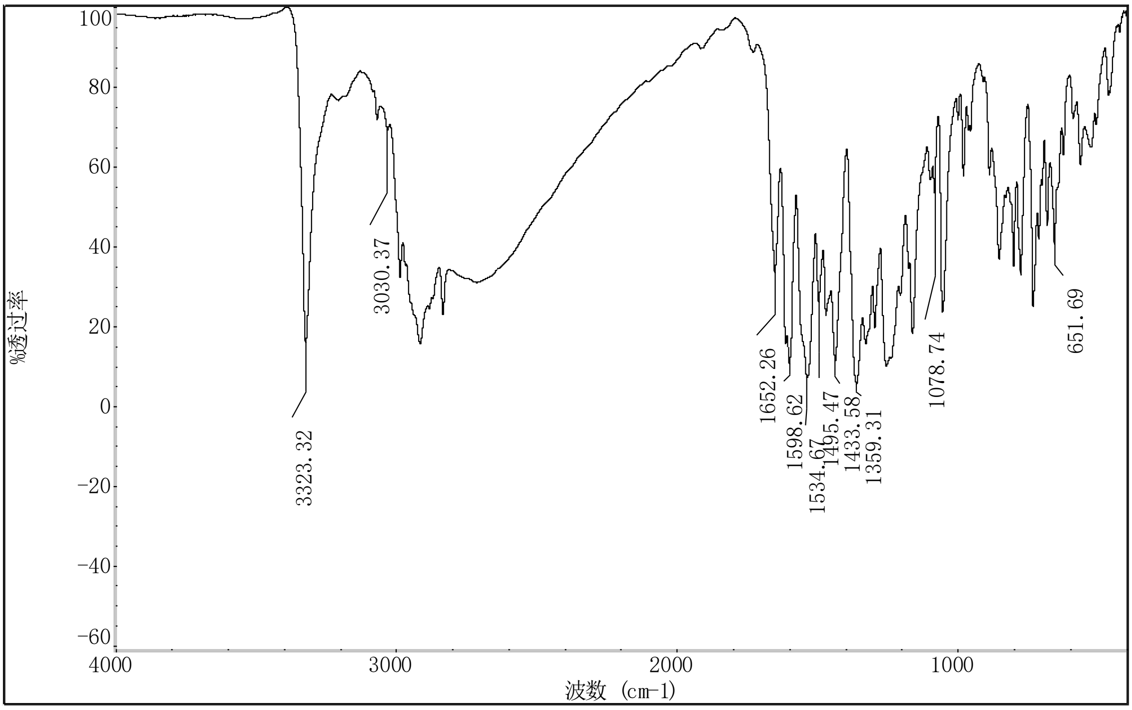

Figure S30. FT-IR spectrum of Compound **6e** (R=*m*-OMe Ph).

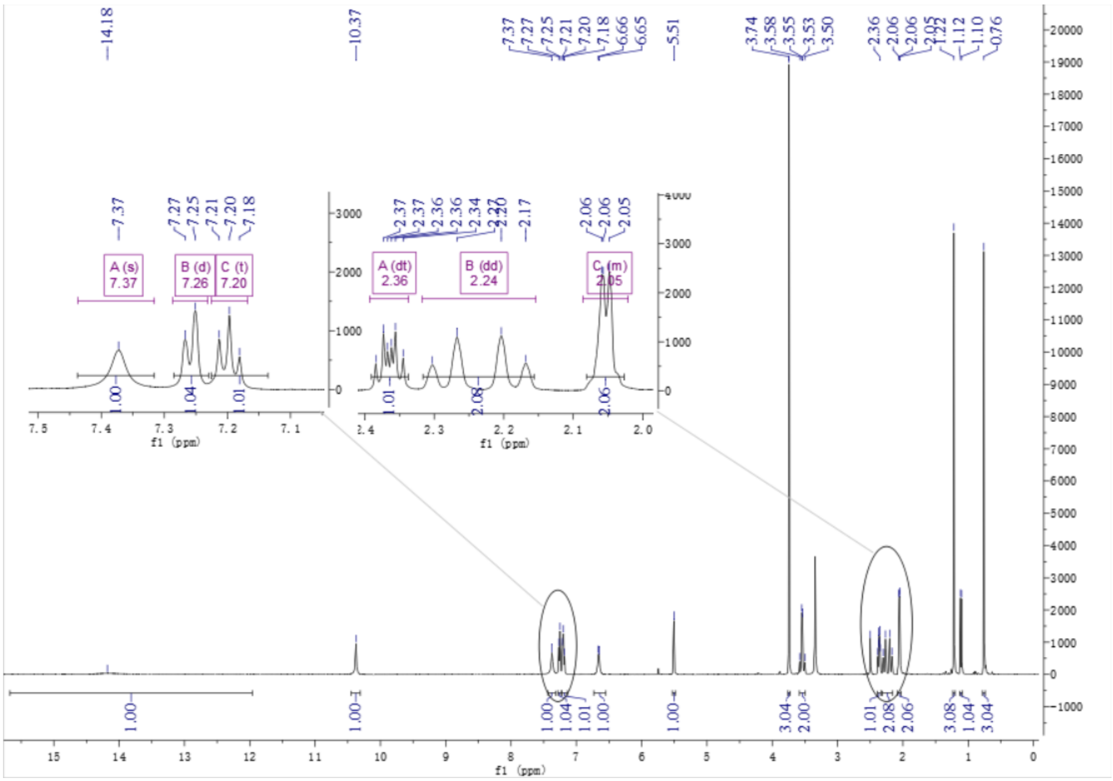

Figure S31. ^{1}H-NMR spectrum of Compound **6e** (R=*m*-OMe Ph) in DMSO.

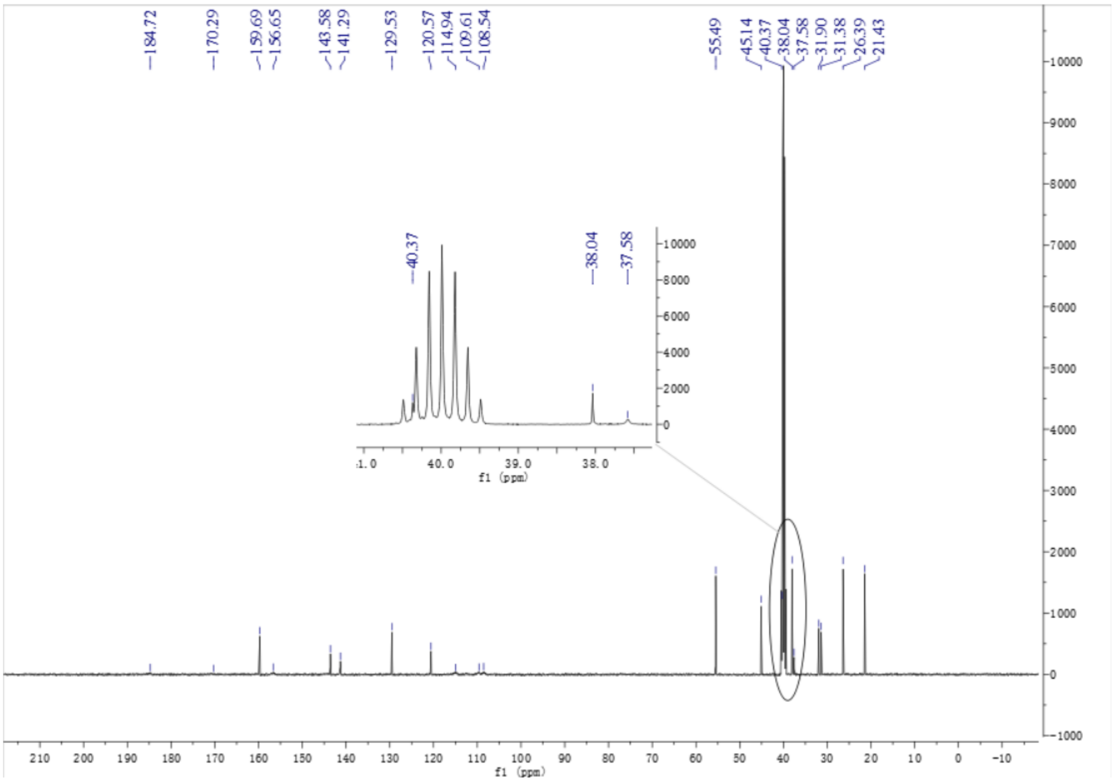

Figure S32. ^{13}C-NMR spectrum of Compound **6e** (R=*m*-OMe Ph) in DMSO.

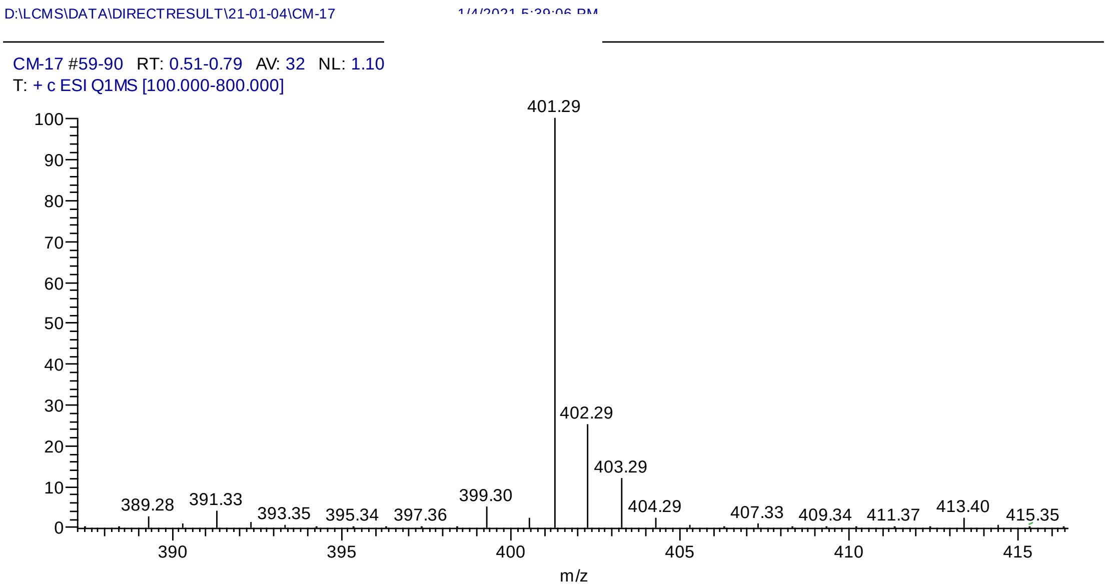

Figure S33. ESI-MS spectrum of Compound **6e** (R=*m*-OMe Ph).

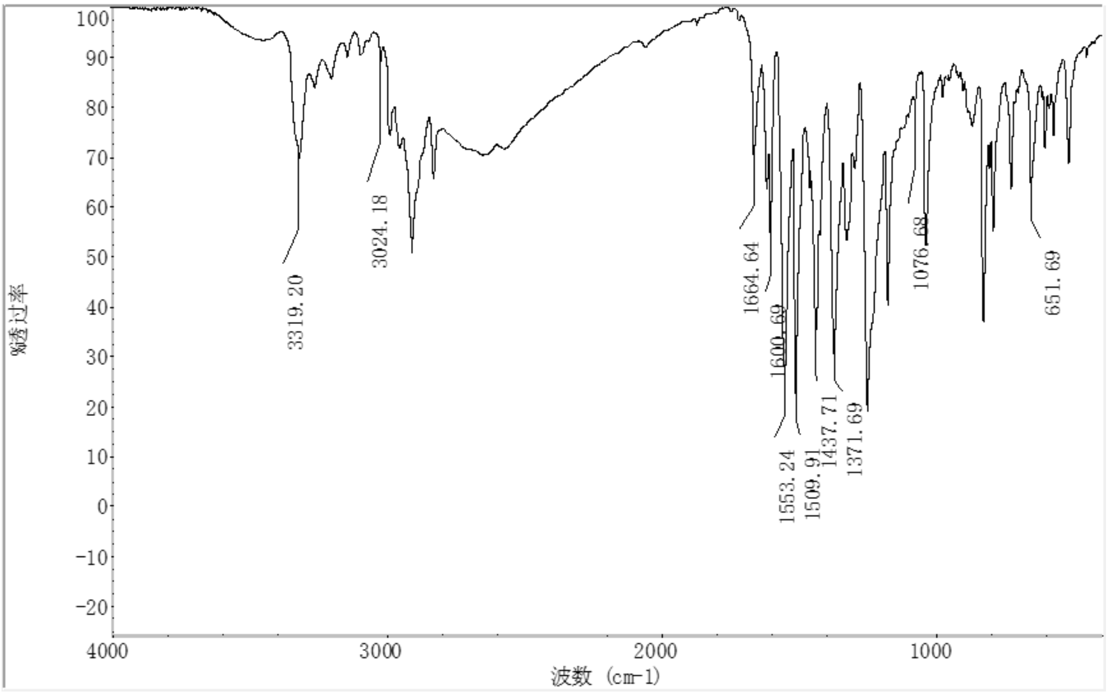

Figure S34. FT-IR spectrum of Compound **6f** (R=*p*-OMe Ph).

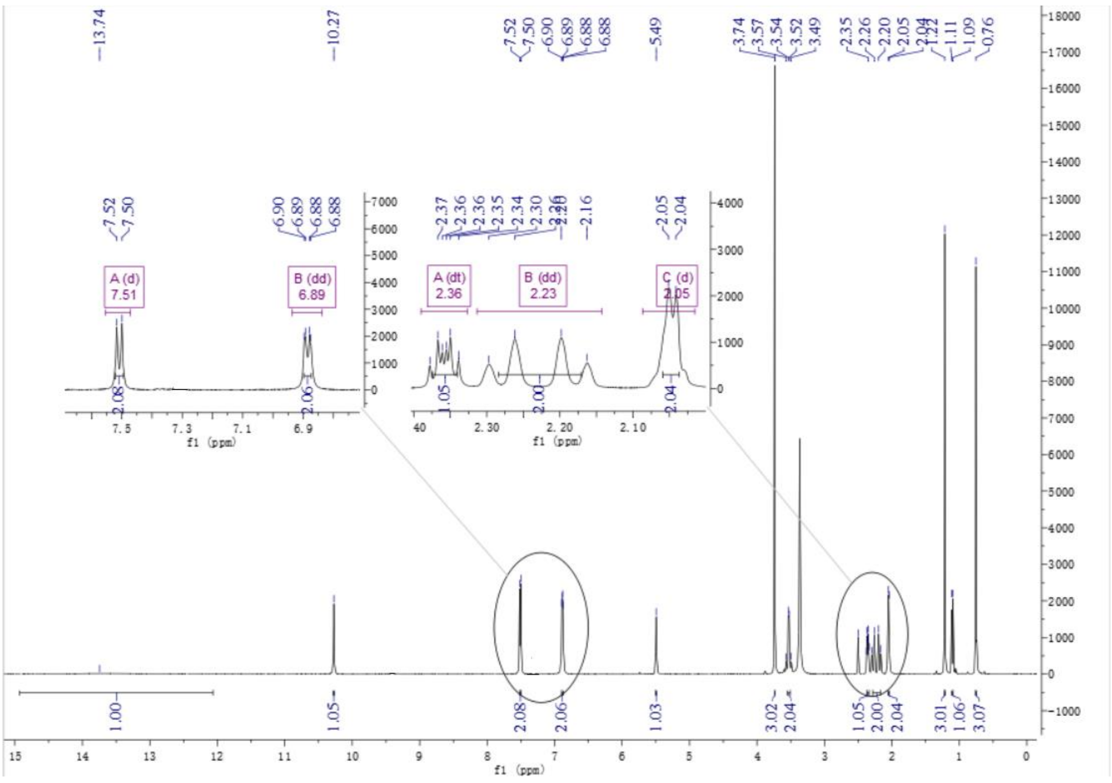

Figure S35. ^{1}H-NMR spectrum of Compound **6f** (R=*p*-OMe Ph) in DMSO.

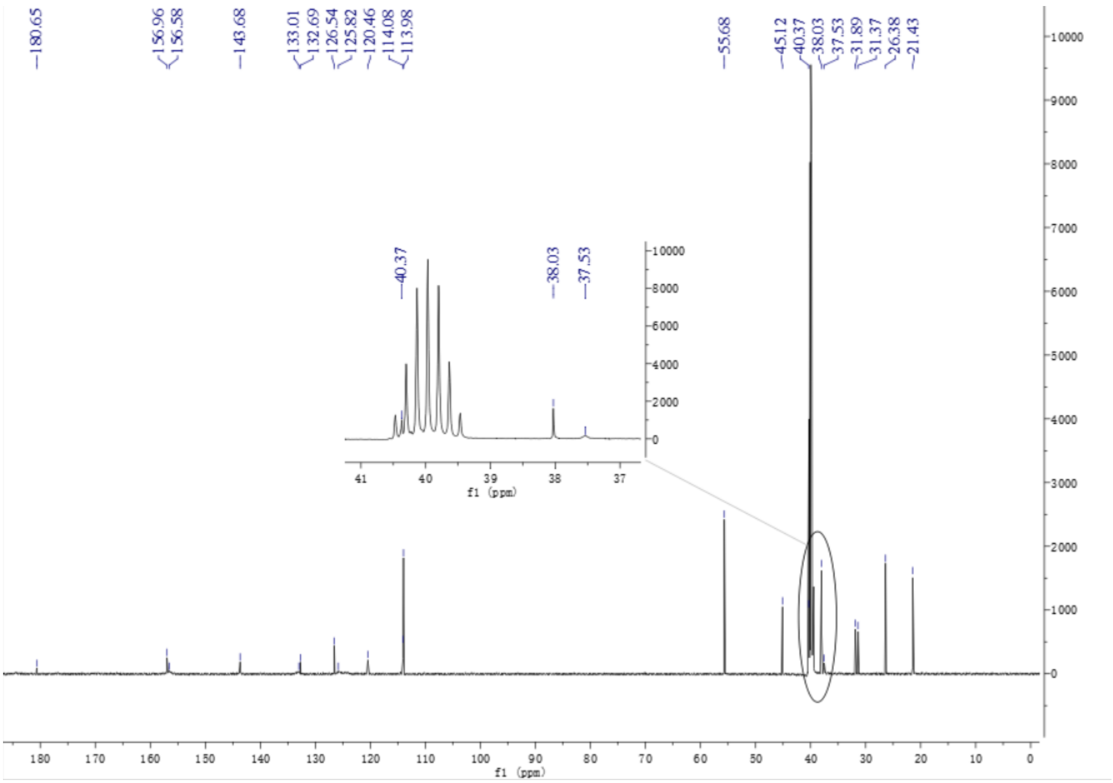

Figure S36. ^{13}C-NMR spectrum of Compound **6f** (R=*p*-OMe Ph) in DMSO.

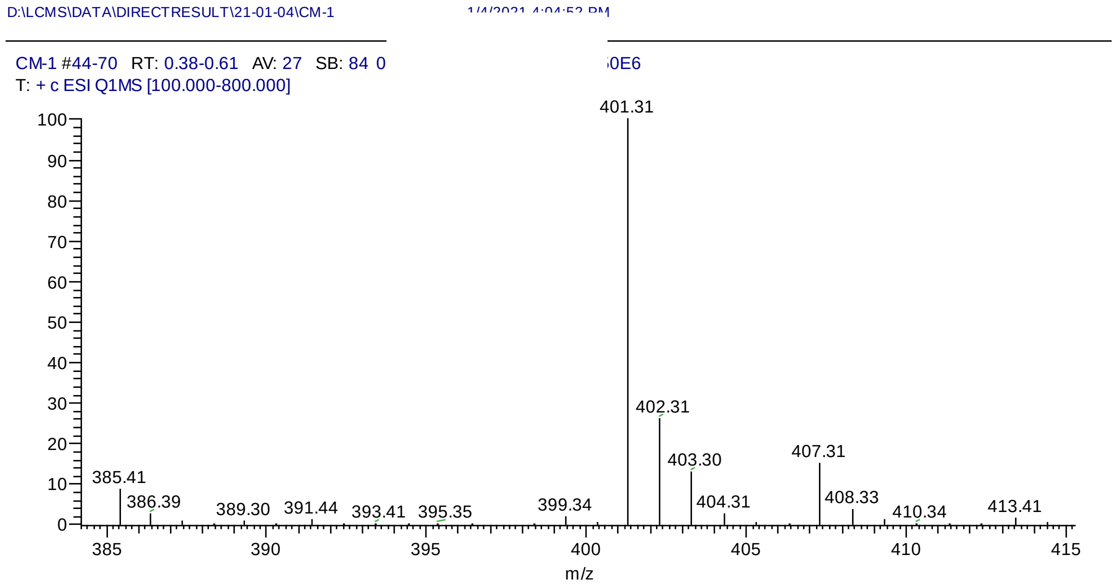

Figure S37. ESI-MS spectrum of Compound **6f** (R=*p*-OMe Ph).

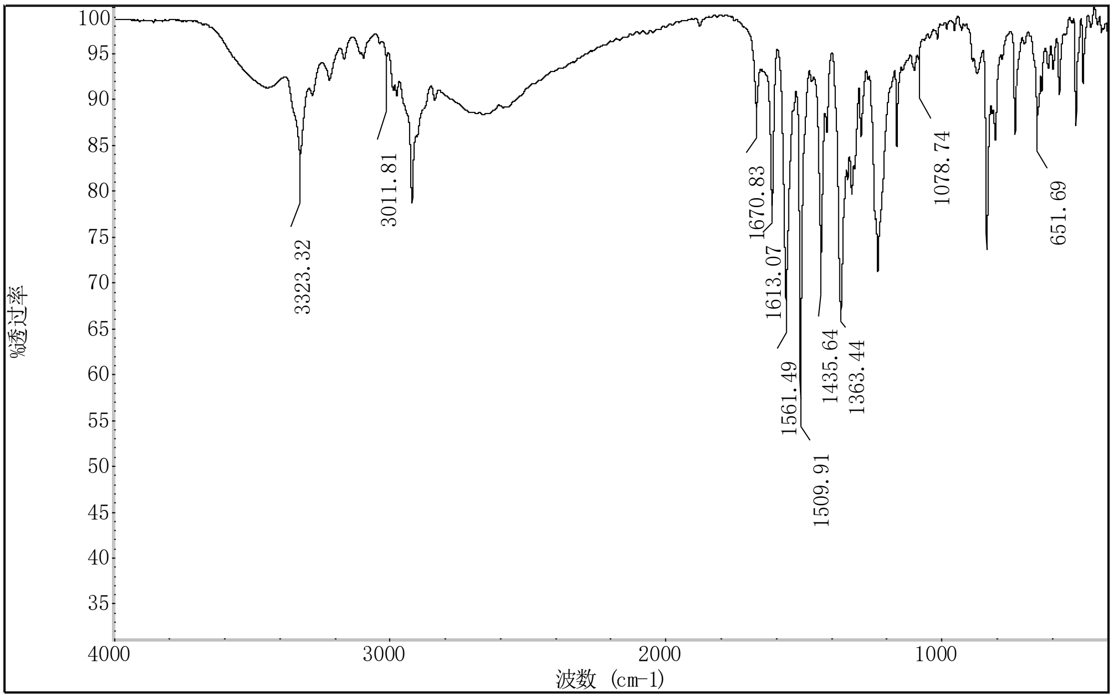

Figure S38. FT-IR spectrum of Compound **6g** (R=*p*-F Ph).

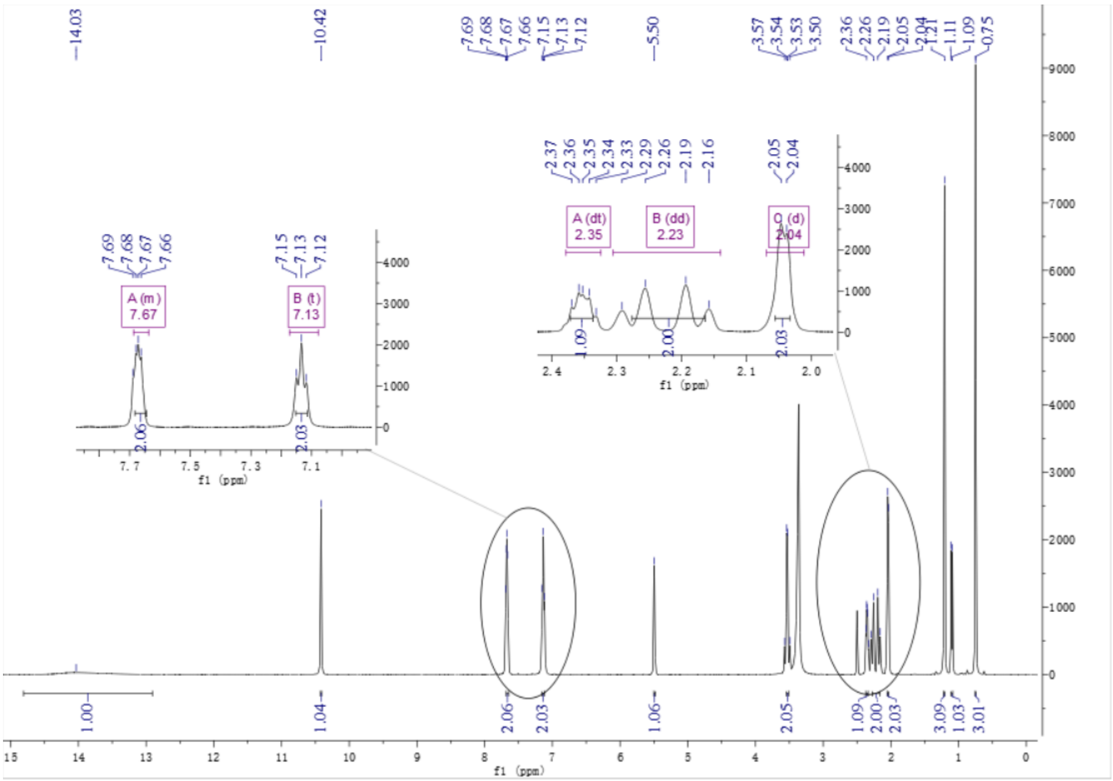

Figure S39. ^{1}H-NMR spectrum of Compound **6g** (R=*p*-F Ph) in DMSO.

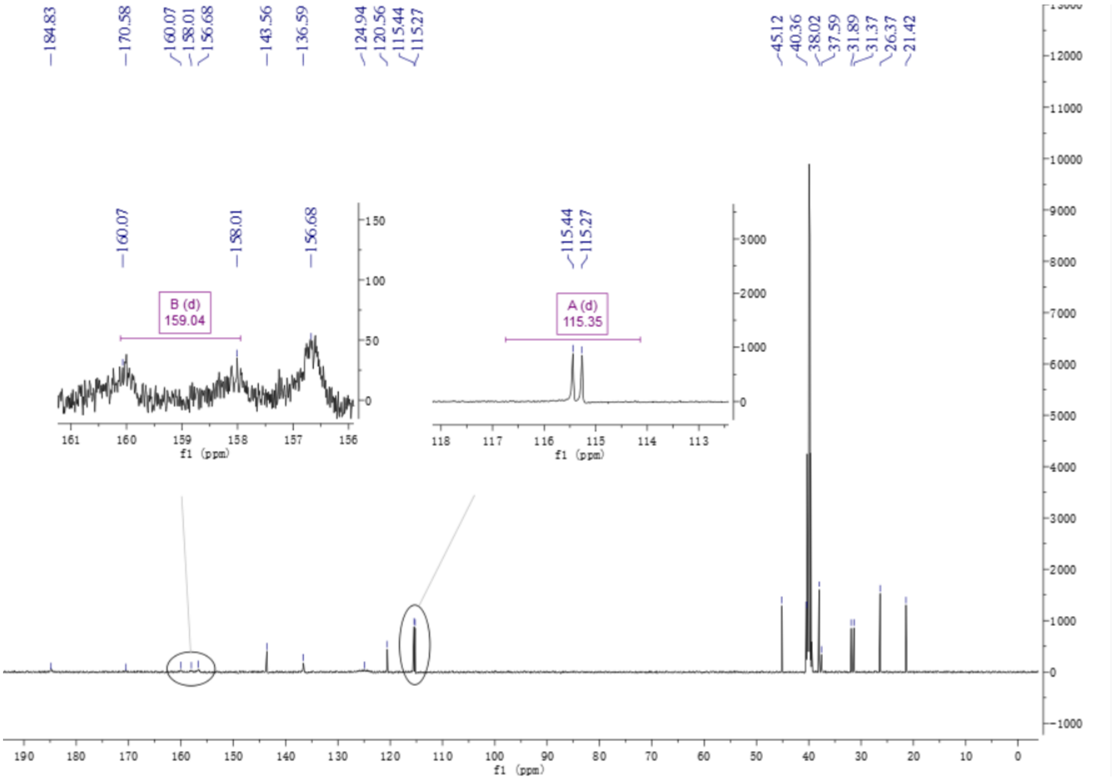

Figure S40. ^{13}C-NMR spectrum of Compound **6g** (R=*p*-F Ph) in DMSO.

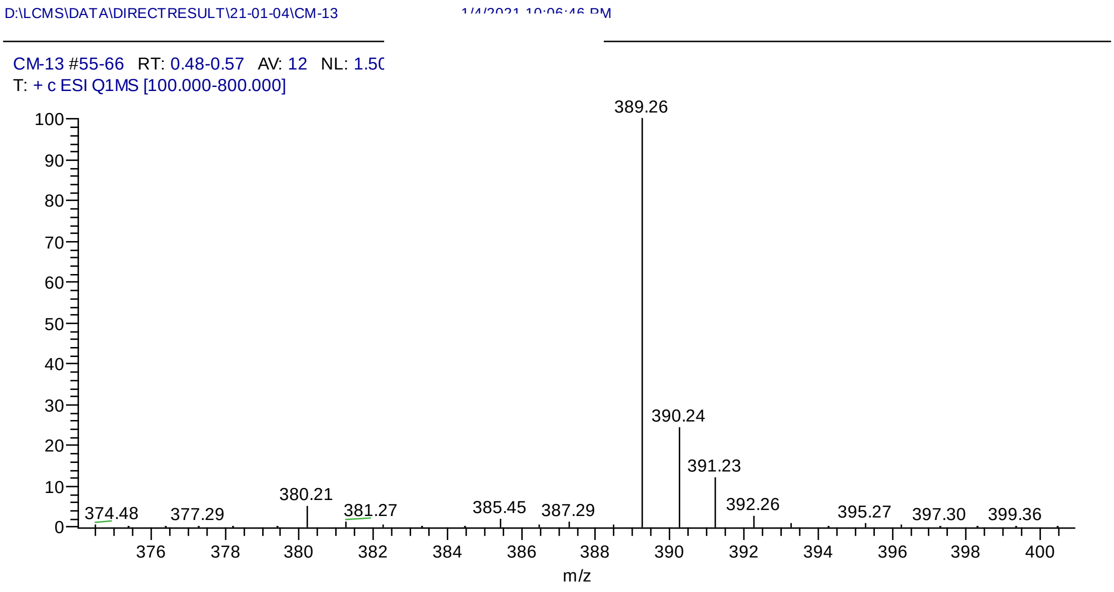

Figure S41. ESI-MS spectrum of Compound **6g** (R=*p*-F Ph).

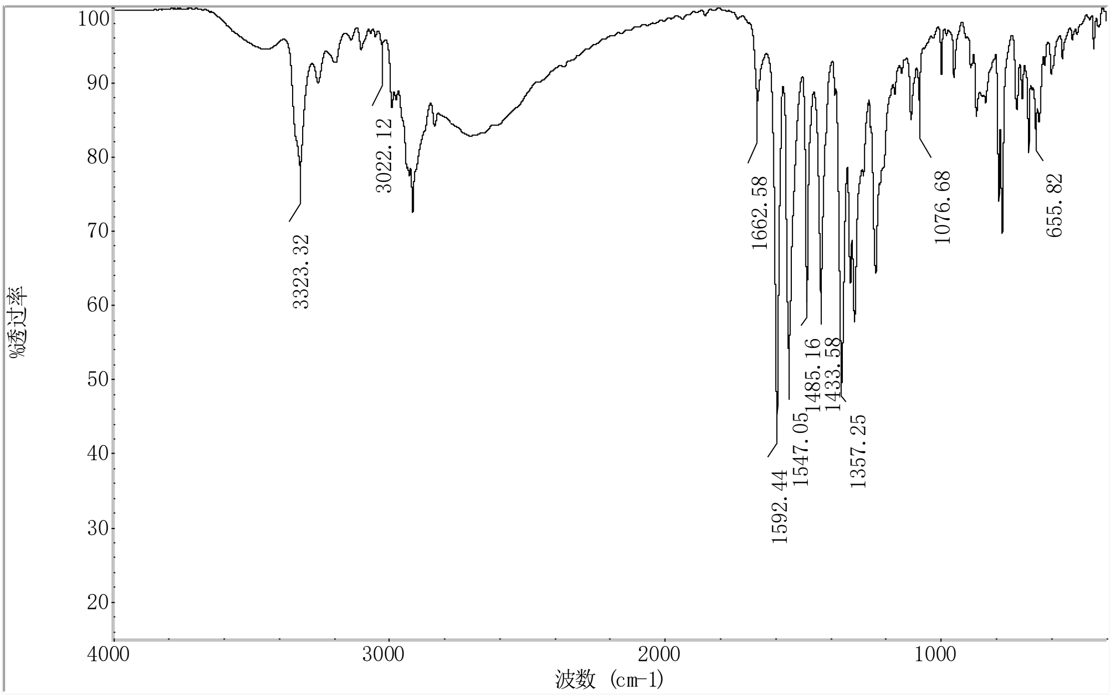

Figure S42. FT-IR spectrum of Compound **6h** (R=*m*-Cl Ph).

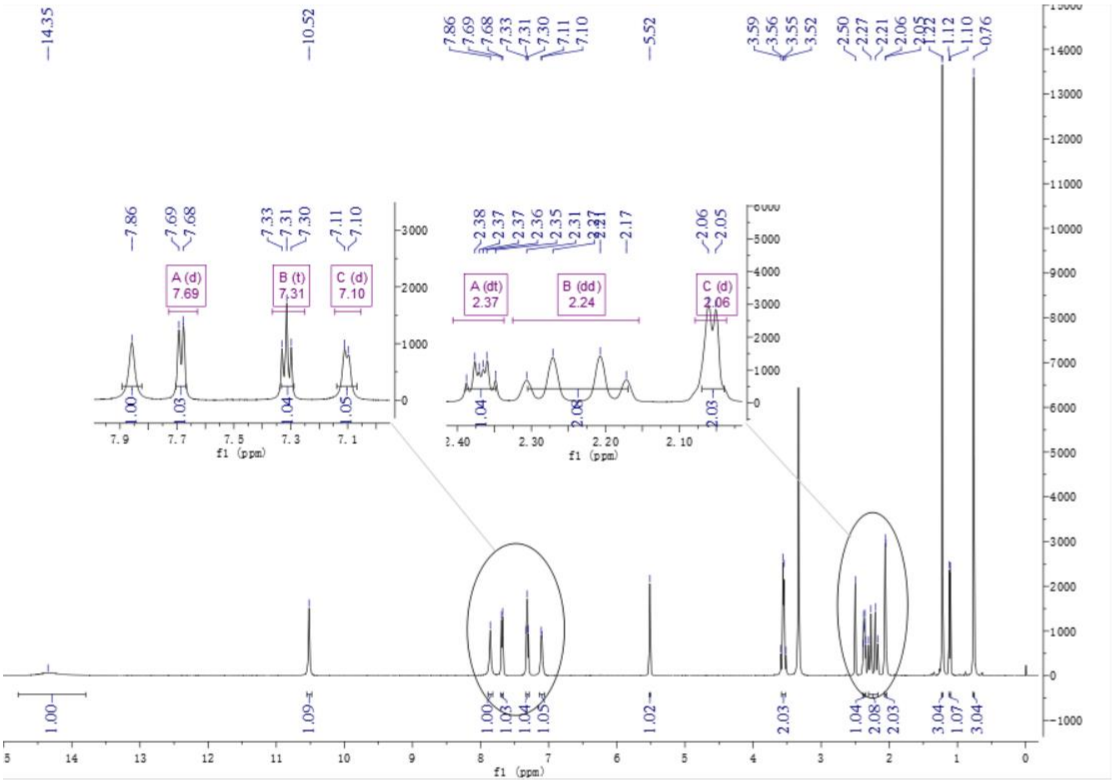

Figure S43. ^{1}H-NMR spectrum of Compound **6h** (R=*m*-Cl Ph) in DMSO.

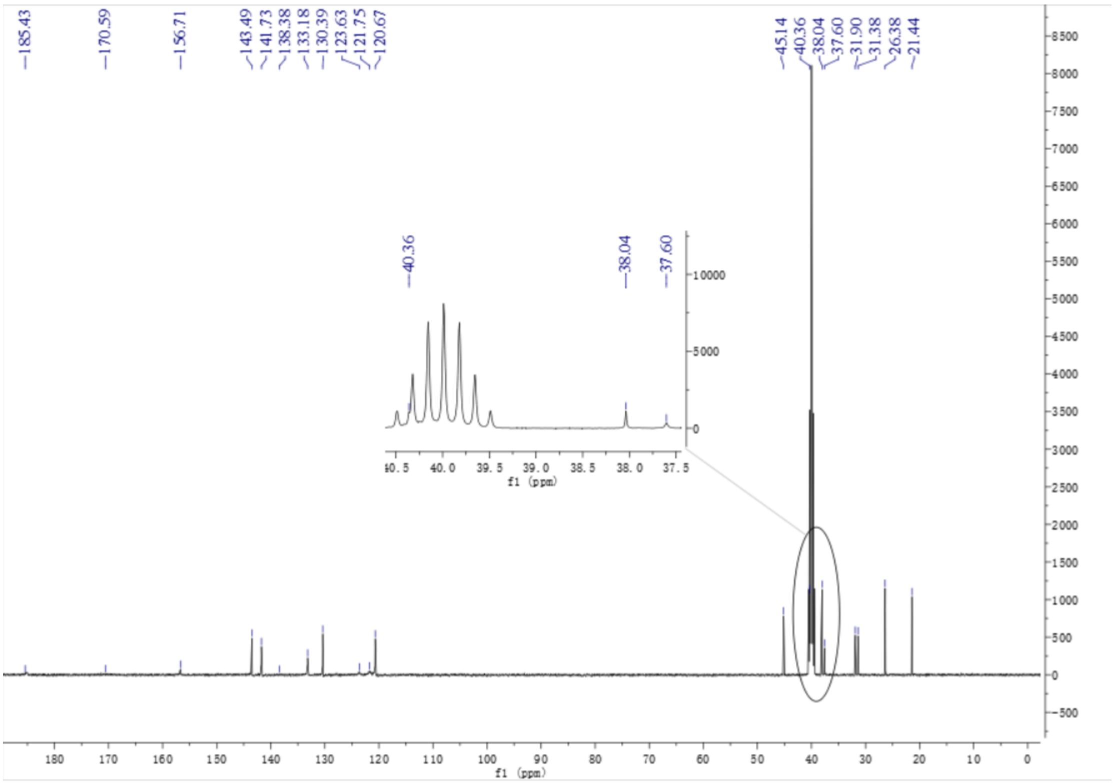

Figure S44. ^{13}C-NMR spectrum of Compound **6h** (R=*m*-Cl Ph) in DMSO.

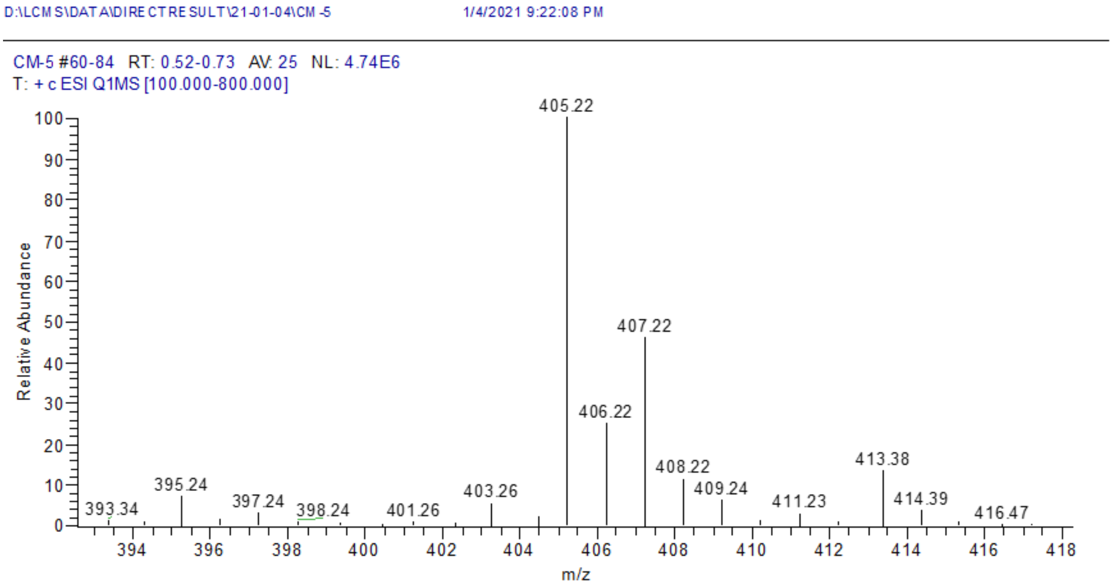

Figure S45. ESI-MS spectrum of Compound **6h** (R=*m*-Cl Ph)

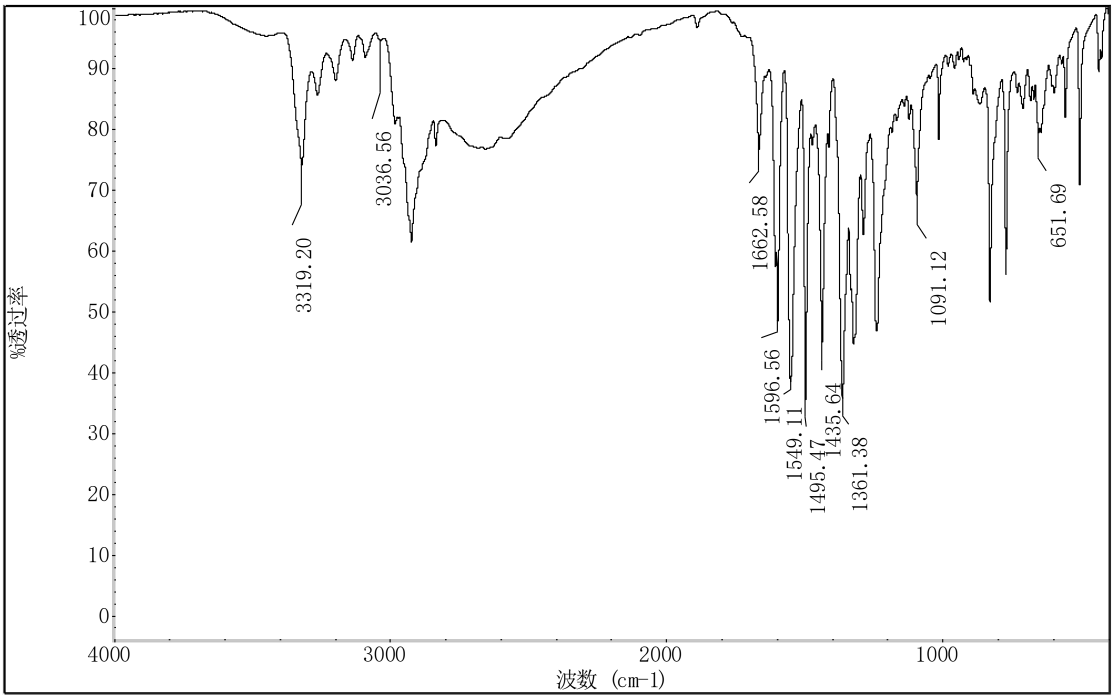

Figure S46. FT-IR spectrum of Compound **6i** (R=*p*-Cl Ph).

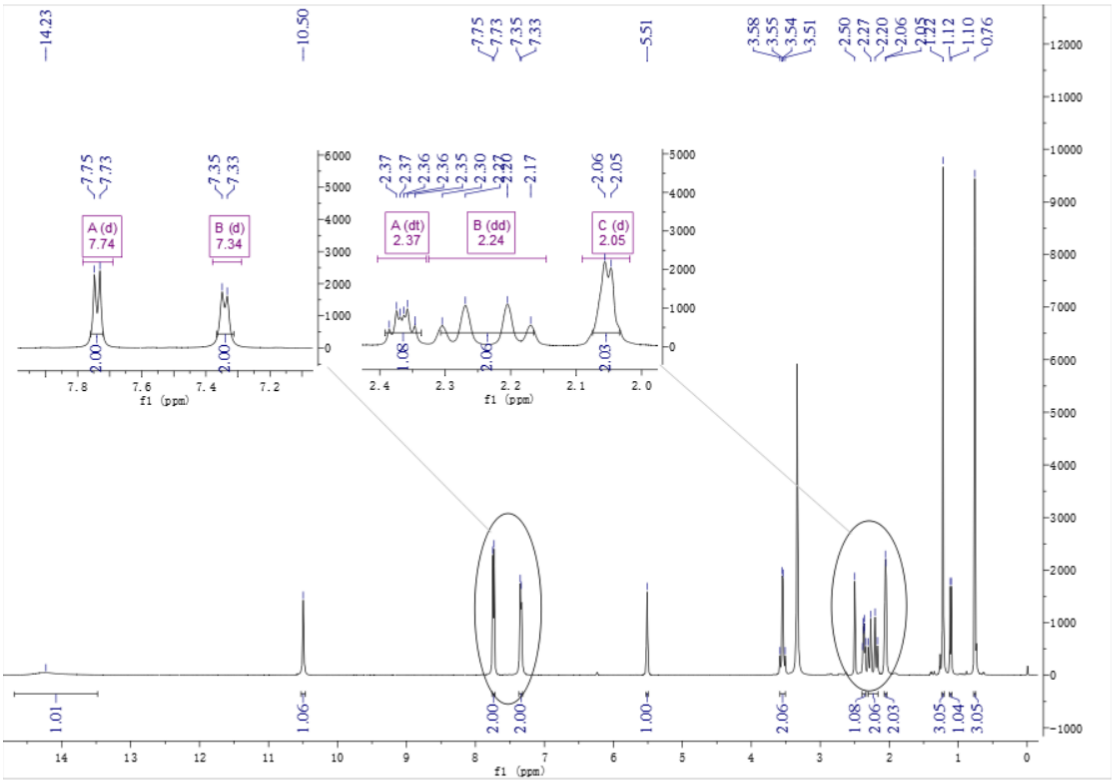

Figure S47. ^{1}H-NMR spectrum of Compound **6i** (R=*p*-Cl Ph) in DMSO.

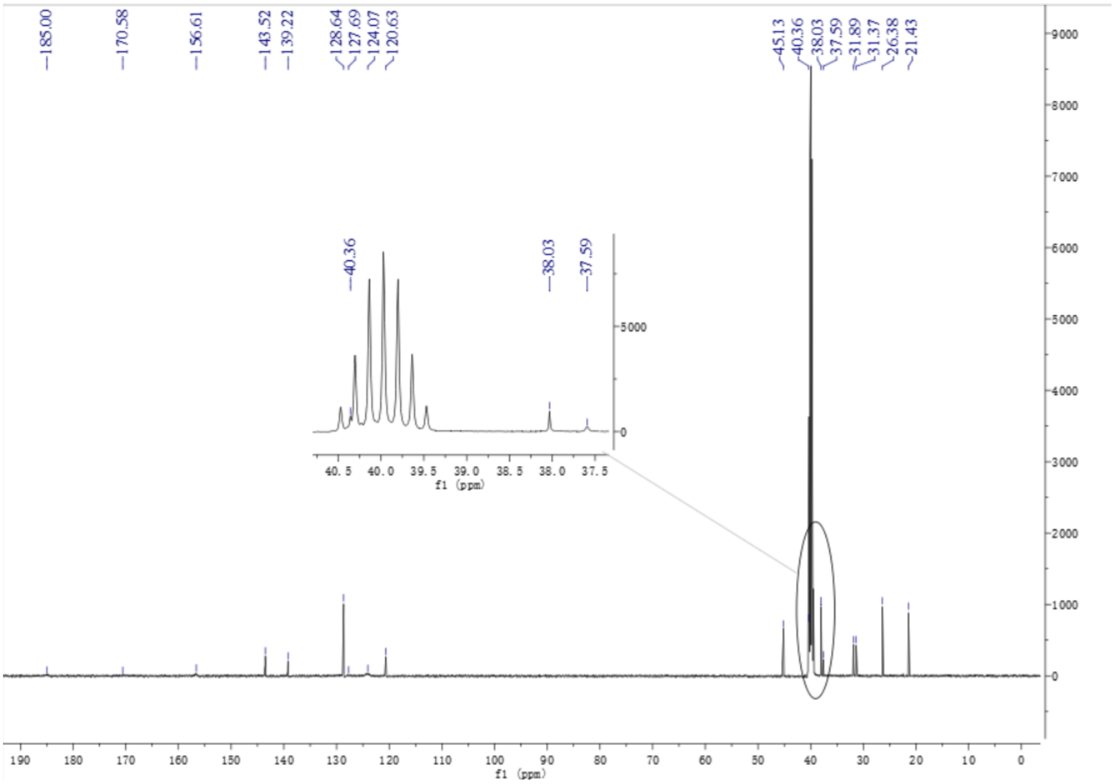

Figure S48. ^{13}C-NMR spectrum of Compound **6i** (R=*p*-Cl Ph) in DMSO.

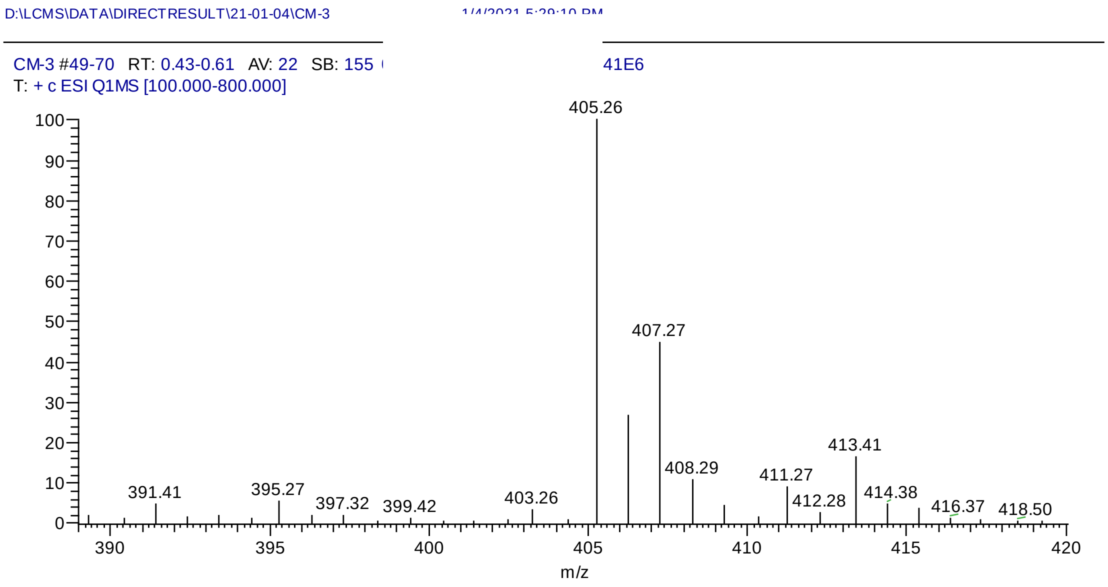

Figure S49. ESI-MS spectrum of Compound **6i** (R=*p*-Cl Ph).

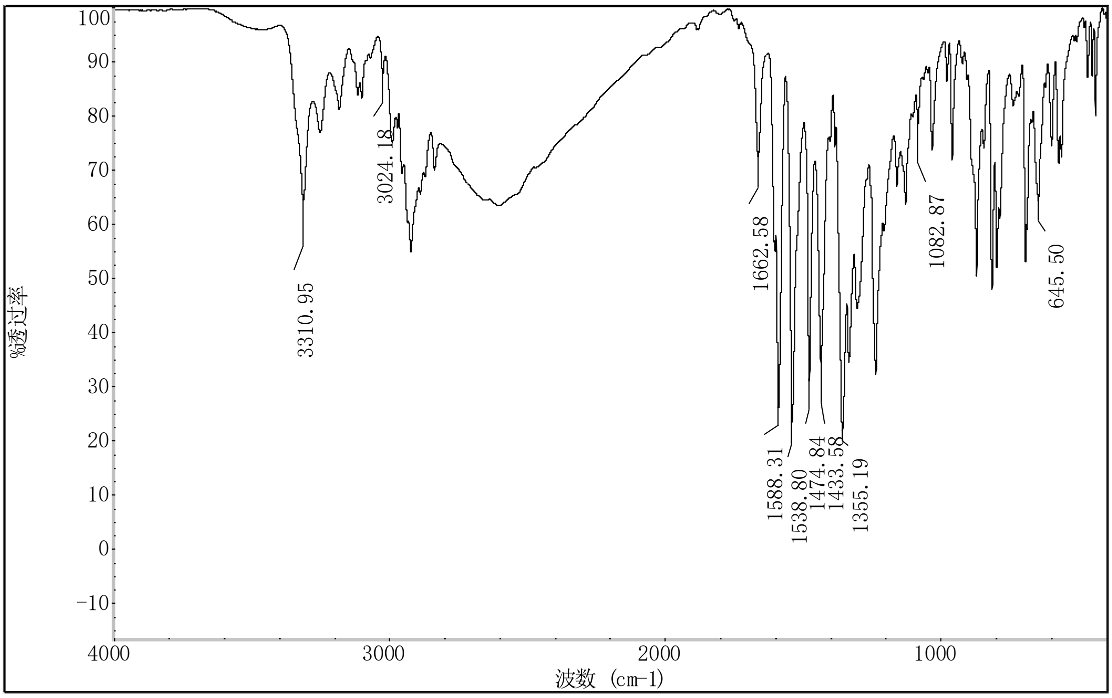

Figure S50. FT-IR spectrum of Compound **6j** (R=*m*, *p*-Cl Ph).

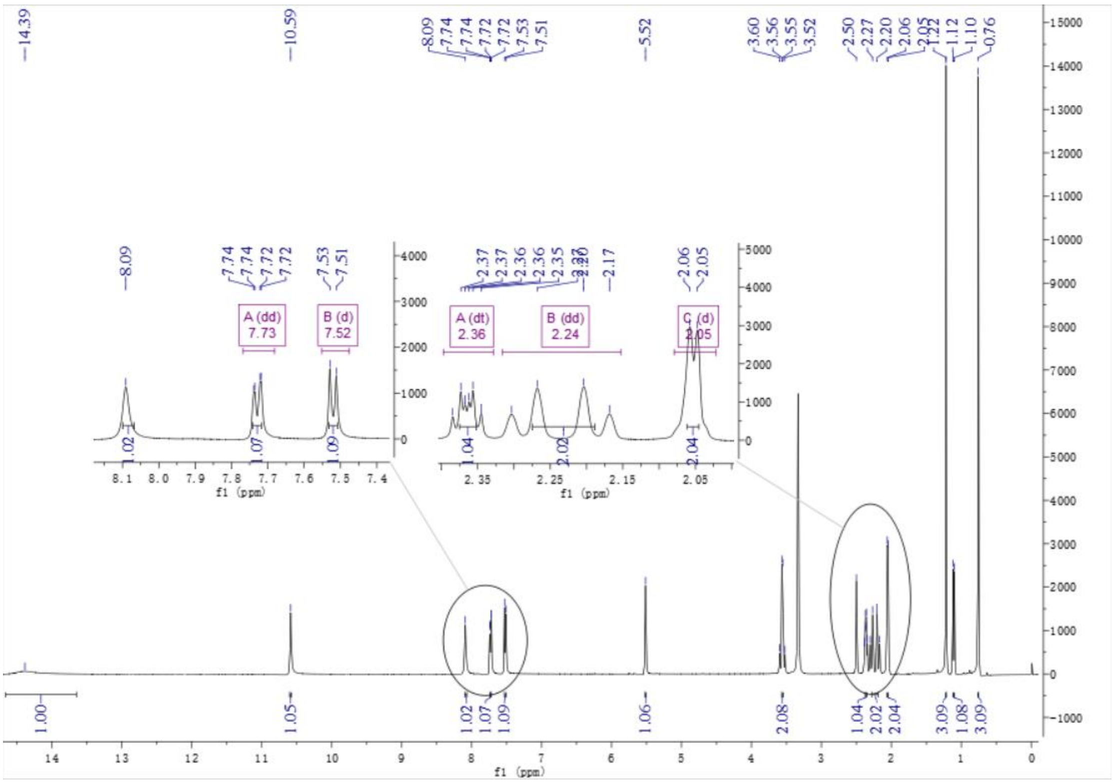

Figure S51. ^{1}H-NMR spectrum of Compound **6j** (R=*m*, *p*-Cl Ph) in DMSO.

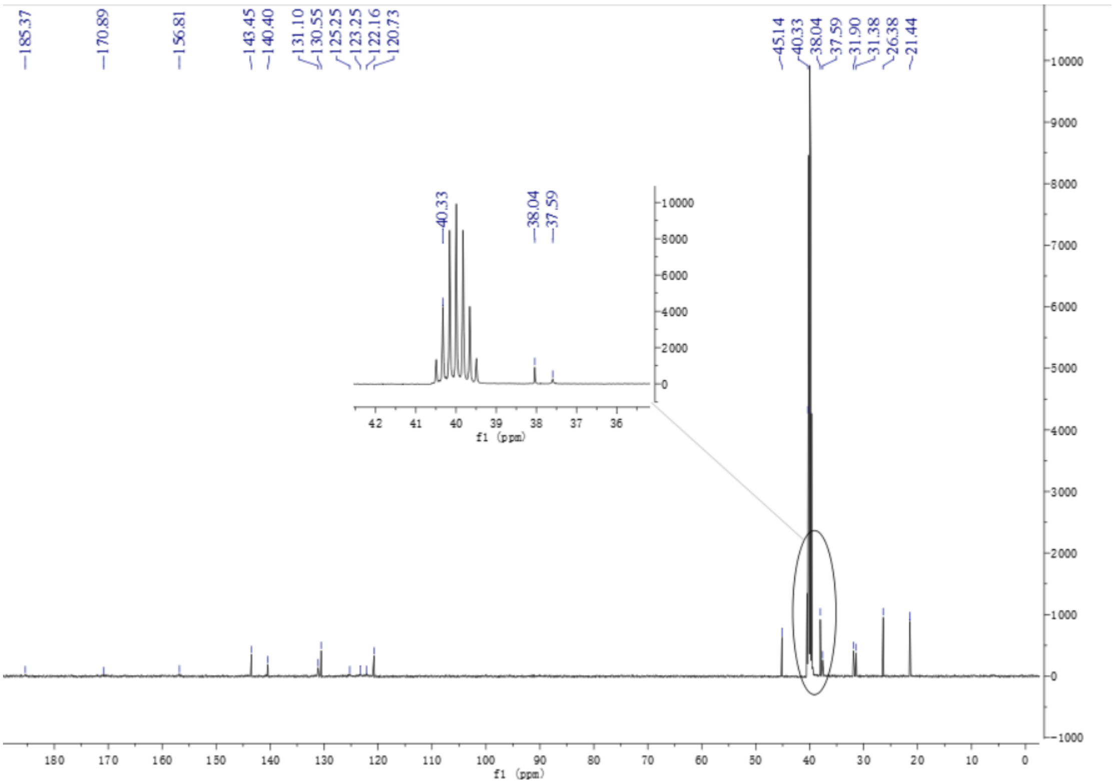

Figure S52. ^{13}C-NMR spectrum of Compound **6j** (R=*m*, *p*-Cl Ph) in DMSO.

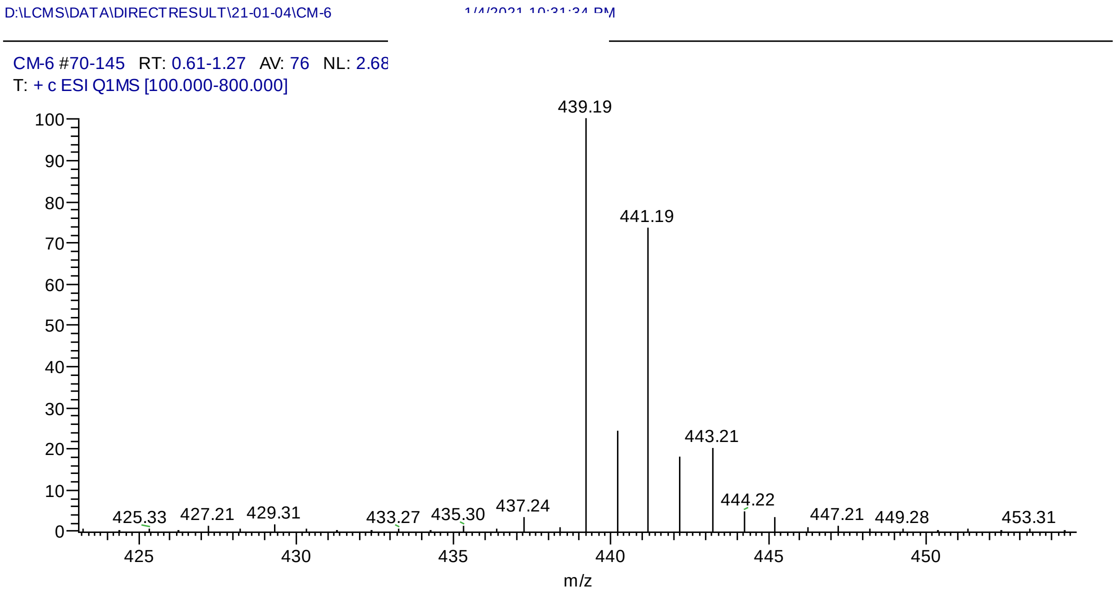

Figure S53. ESI-MS spectrum of Compound **6j** (R=*m*, *p*-Cl Ph).

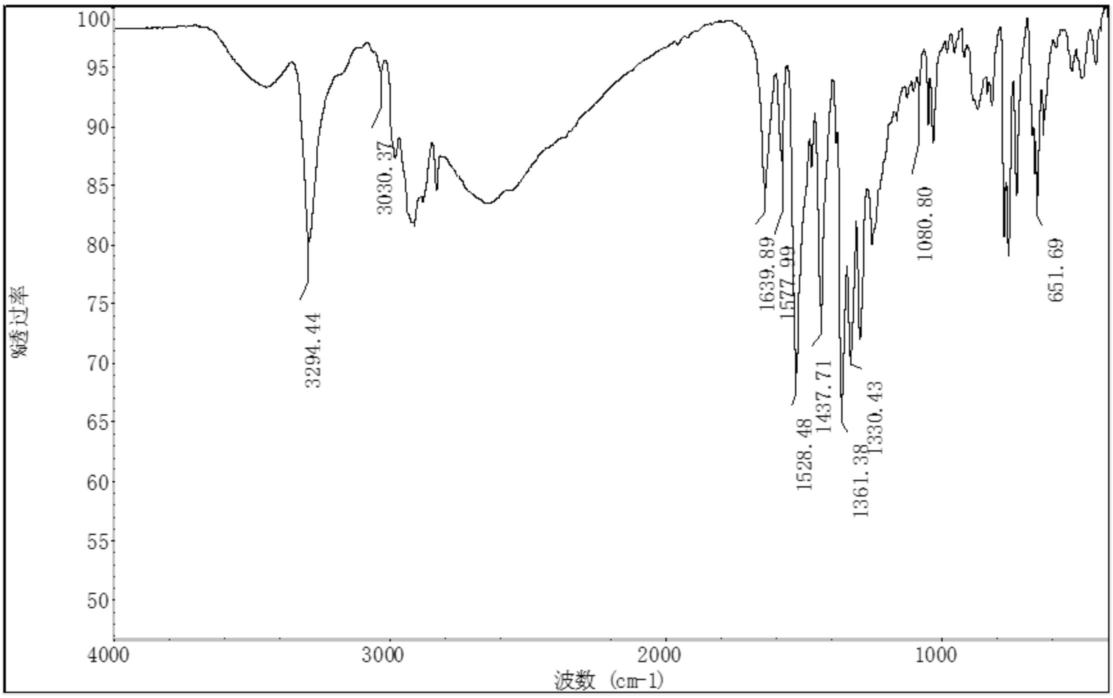

Figure S54. FT-IR spectrum of Compound **6k** (R=*o*-Br Ph).

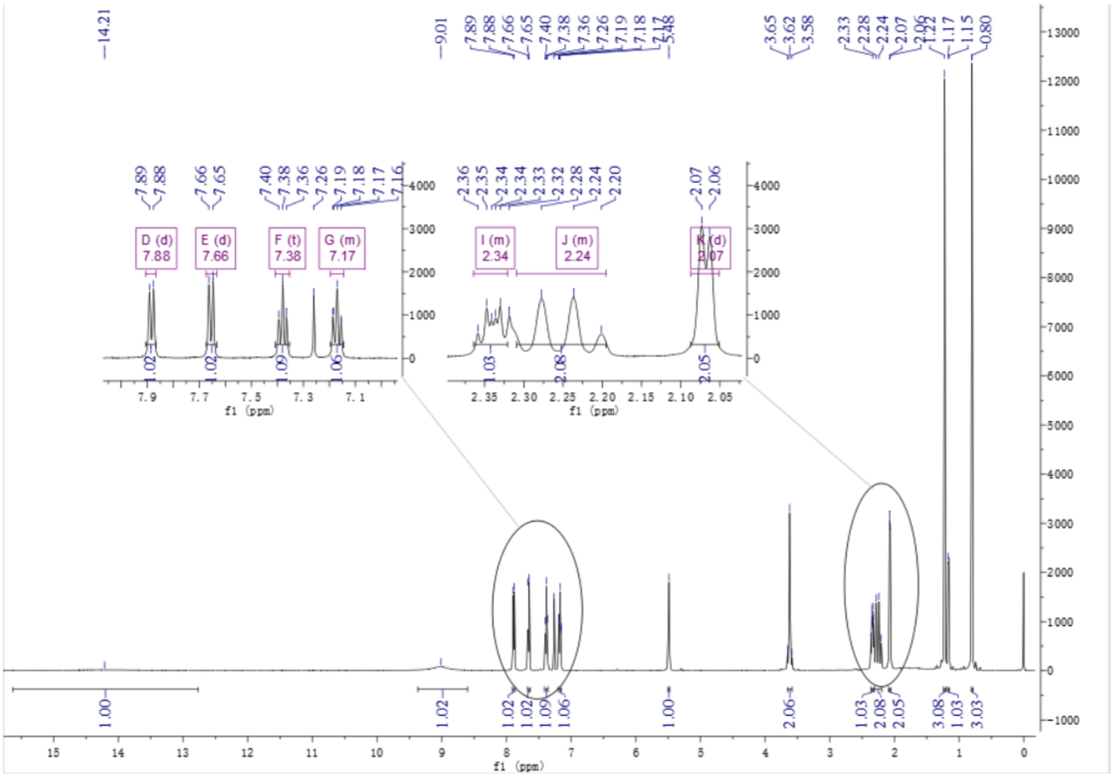

Figure S55. ^{1}H-NMR spectrum of Compound **6k** (R=*o*-Br Ph) in $CDCl_3$.

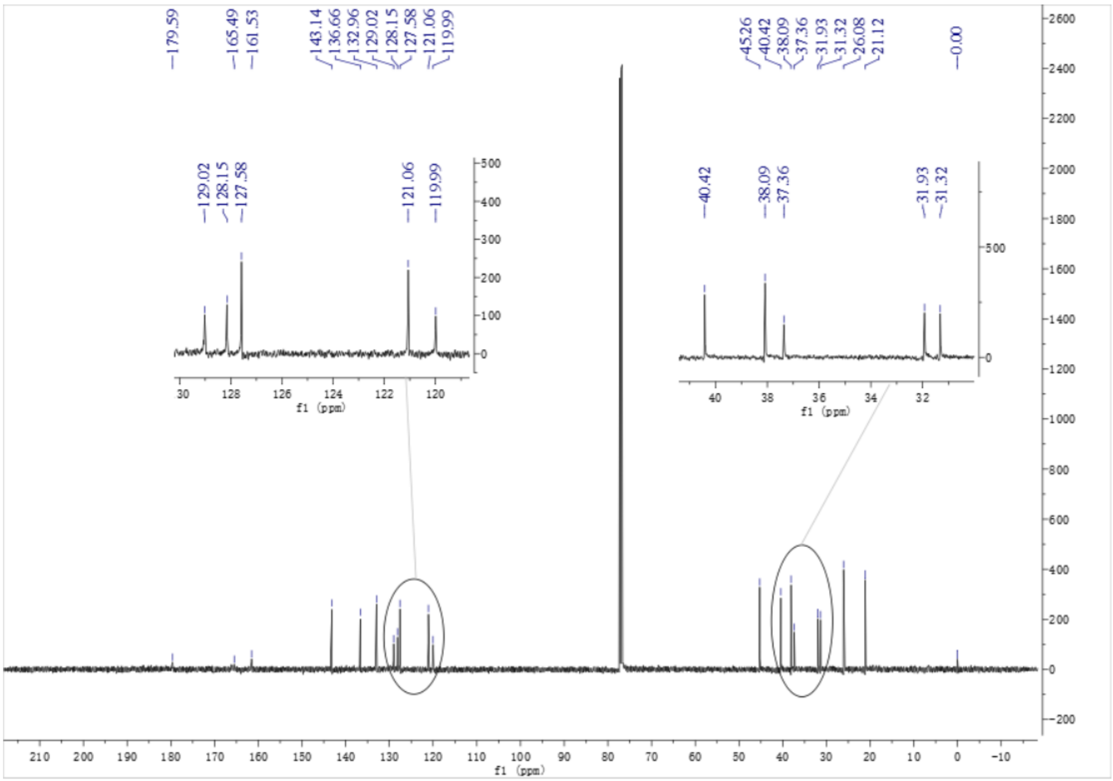

Figure S56. ^{13}C-NMR spectrum of Compound **6k** (R=*o*-Br Ph) in $CDCl_3$.

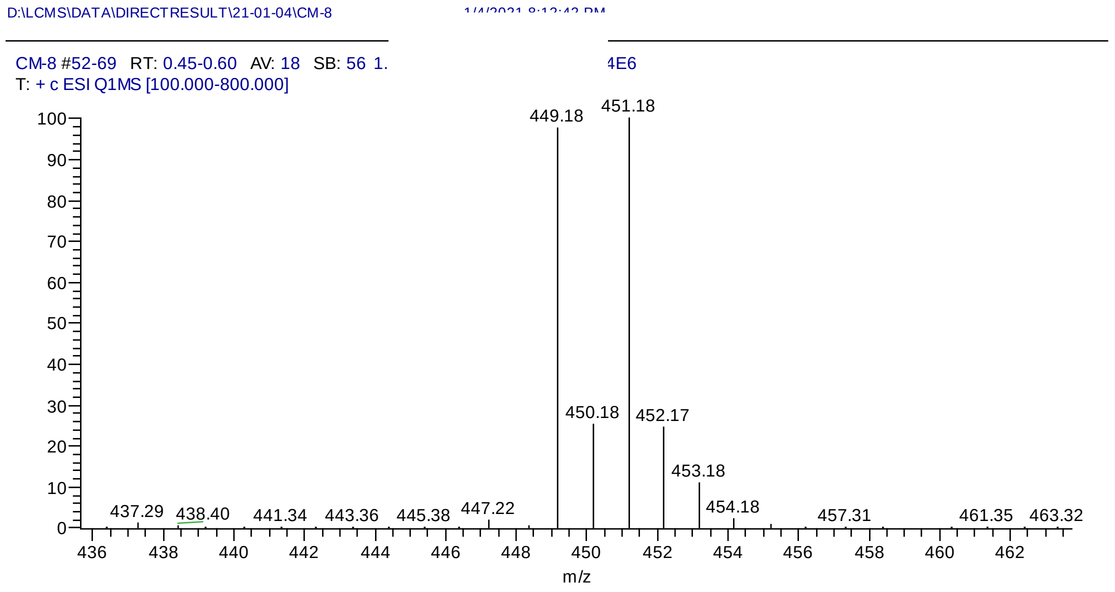

Figure S57. ESI-MS spectrum of Compound **6k** (R=*o*-Br Ph).

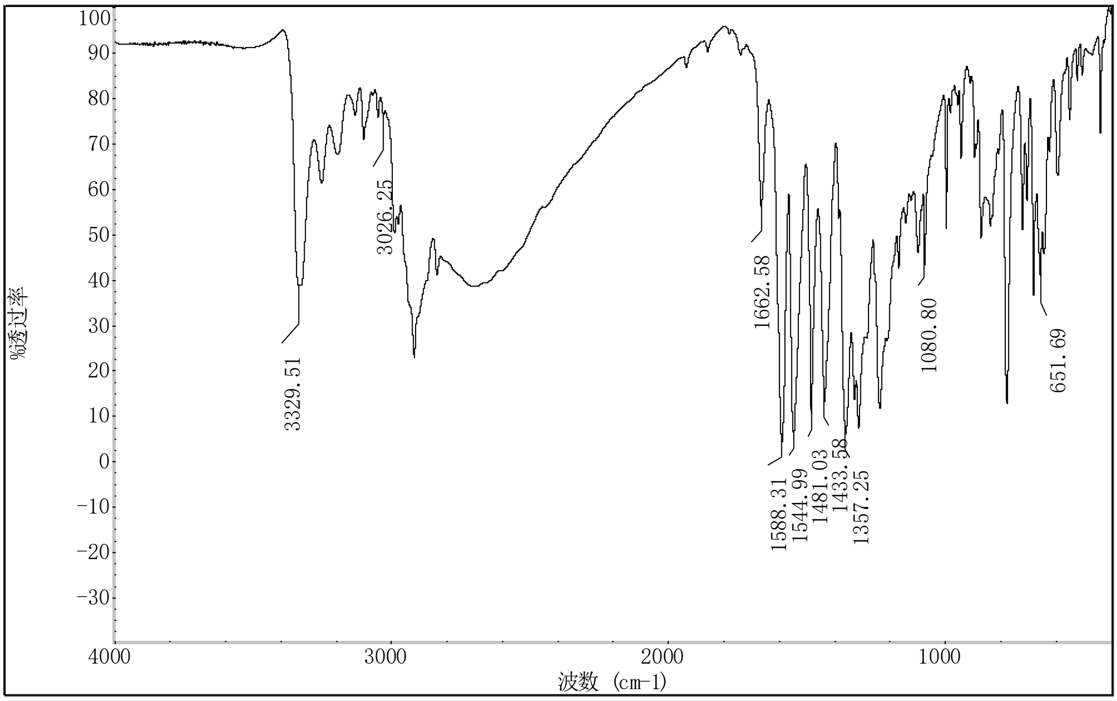

Figure S58. FT-IR spectrum of Compound **6l** (R=*m*-Br Ph).

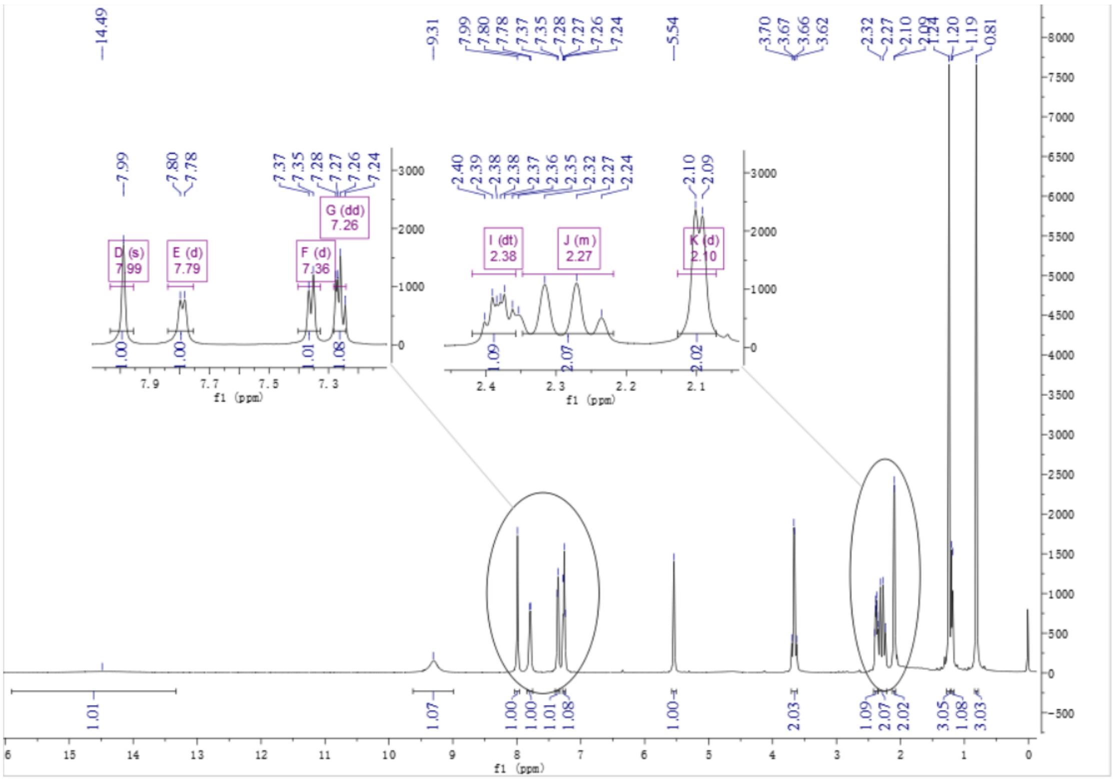

Figure S59. ^{1}H-NMR spectrum of Compound **6l** (R=*m*-Br Ph) in $CDCl_3$.

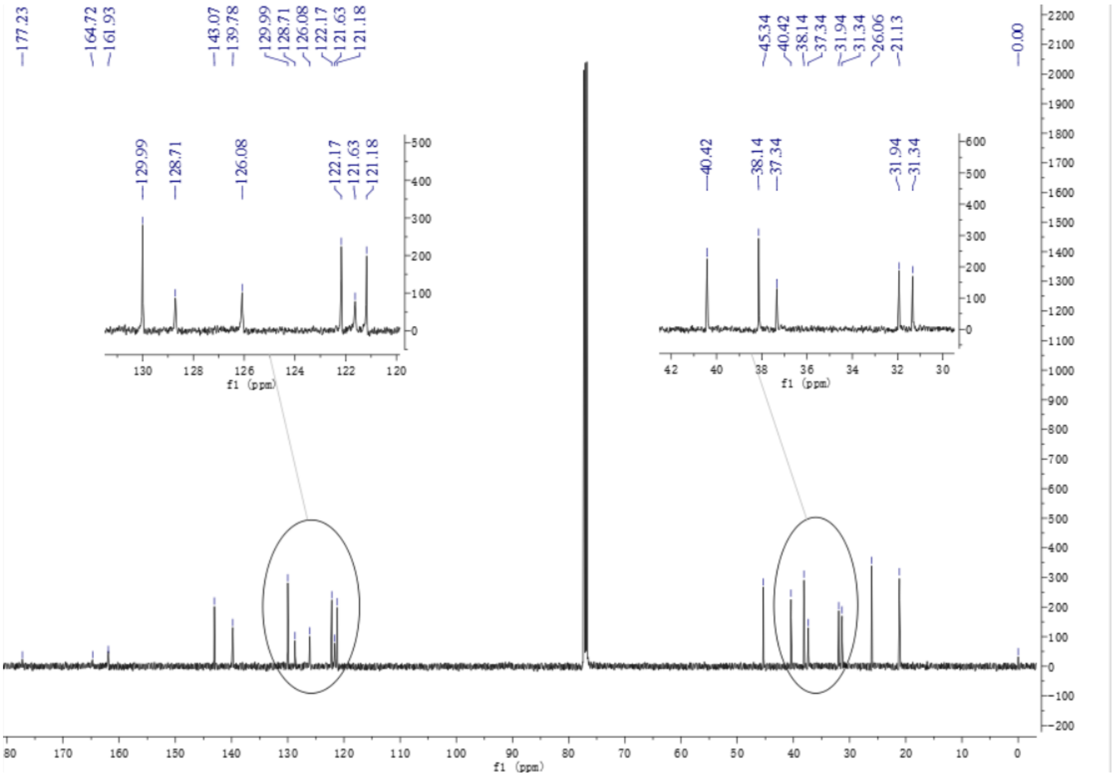

Figure S60. ^{13}C-NMR spectrum of Compound **6l** (R=*m*-Br Ph) in $CDCl_3$.

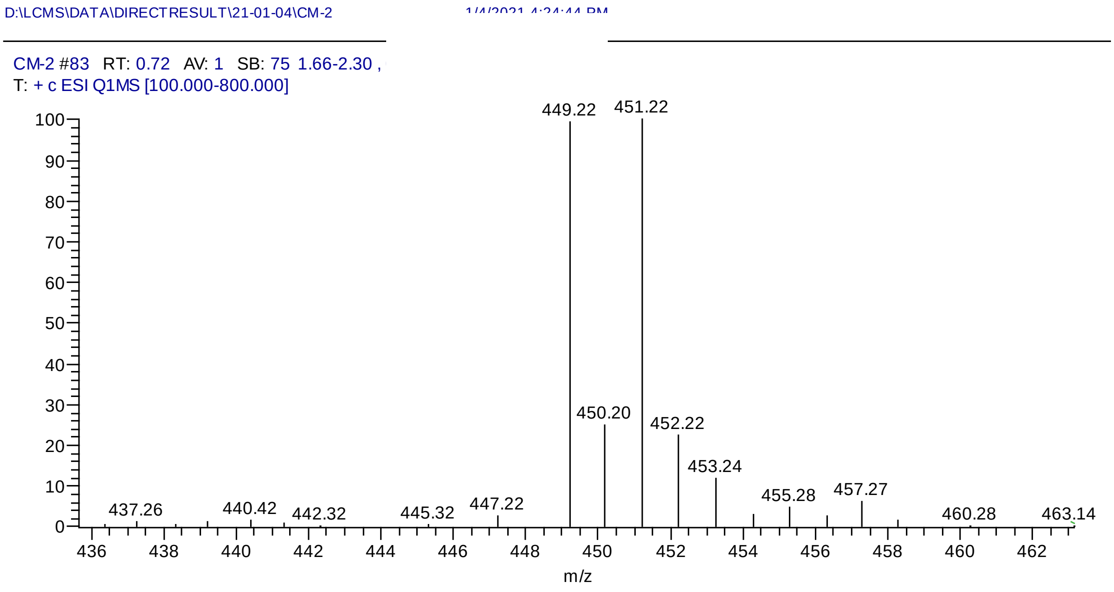

Figure S61. ESI-MS spectrum of Compound **6l** (R=*m*-Br Ph).

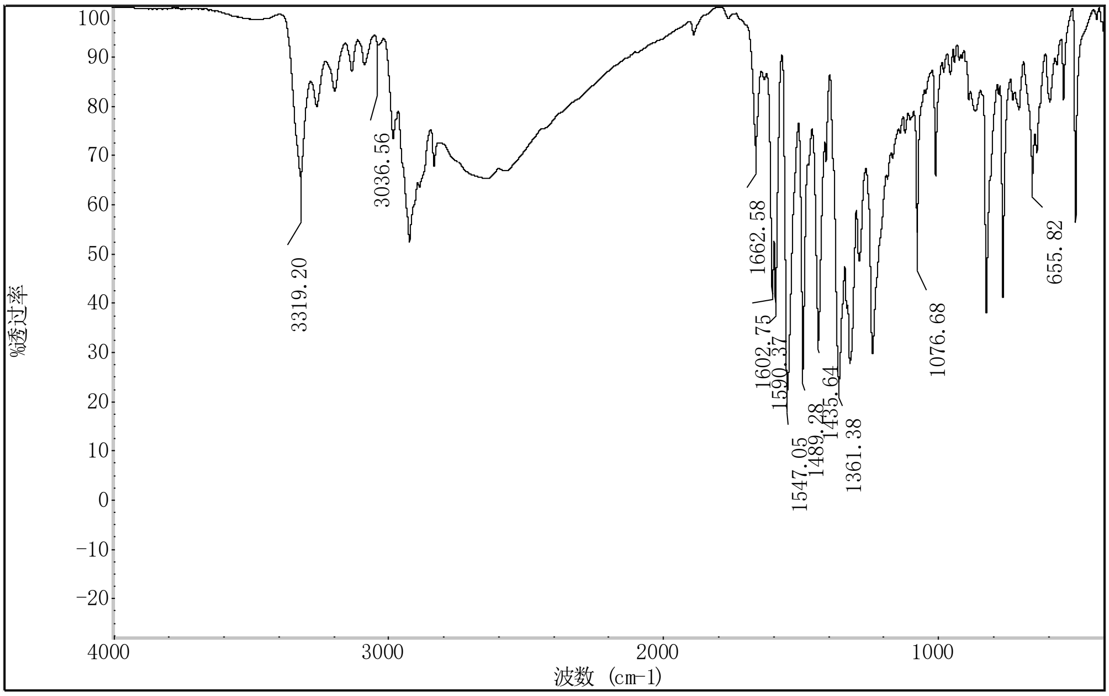

Figure S62. FT-IR spectrum of Compound **6m** (R=*p*-Br Ph).

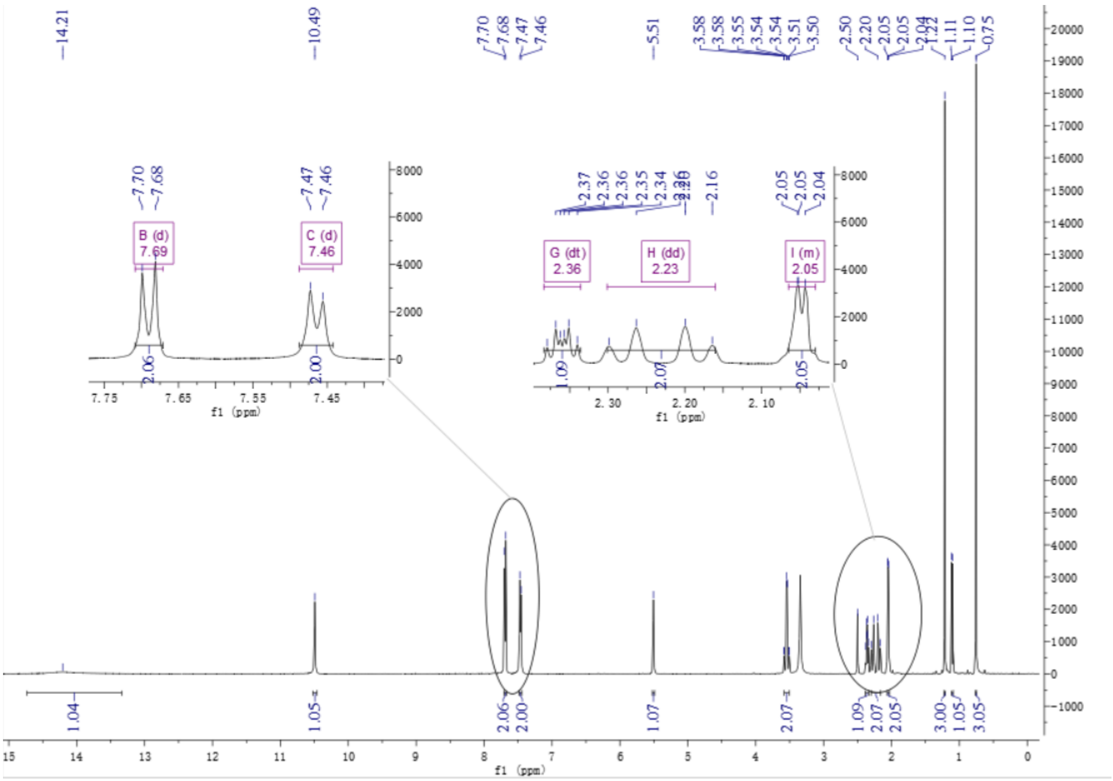

Figure S63. ^{1}H-NMR spectrum of Compound **6m** (R=*p*-Br Ph) in DMSO.

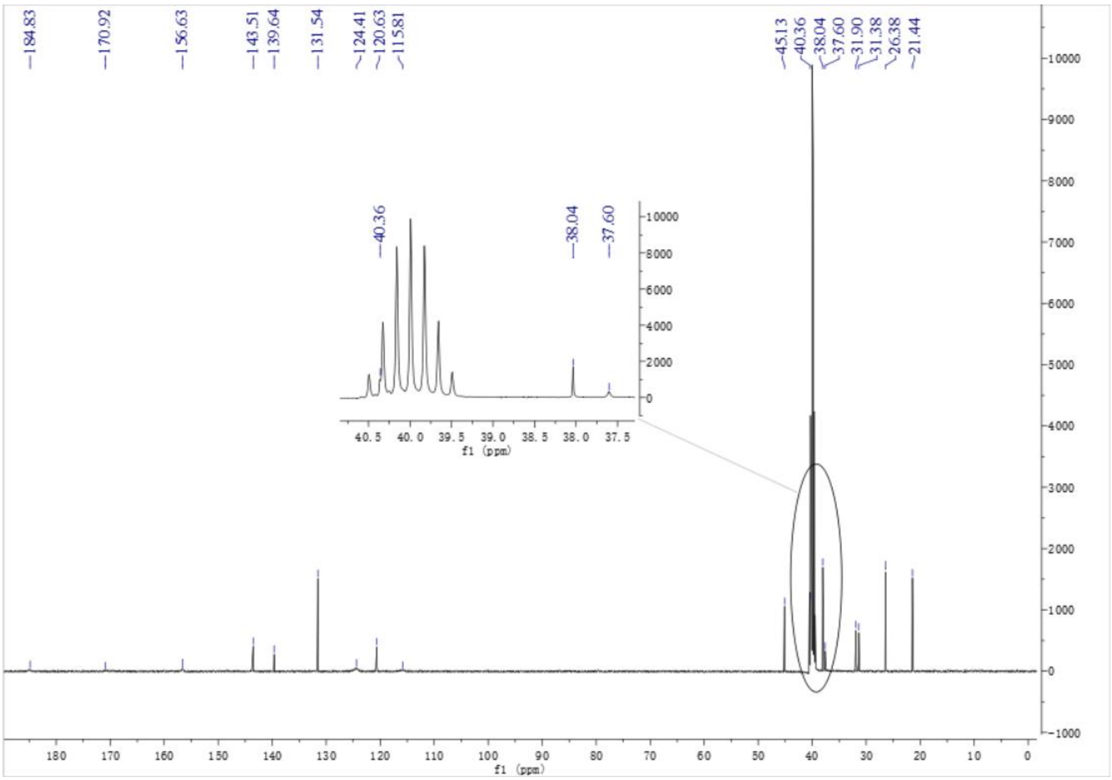

Figure S64. ^{13}C-NMR spectrum of Compound **6m** (R=*p*-Br Ph) in DMSO.

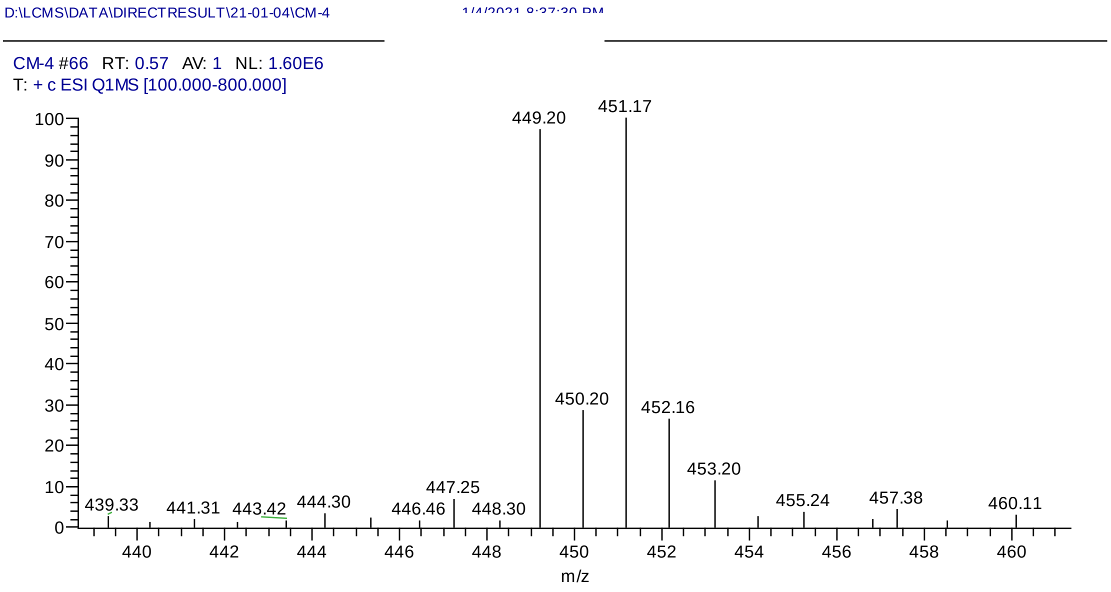

Figure S65. ESI-MS spectrum of Compound **6m** (R=*p*-Br Ph).

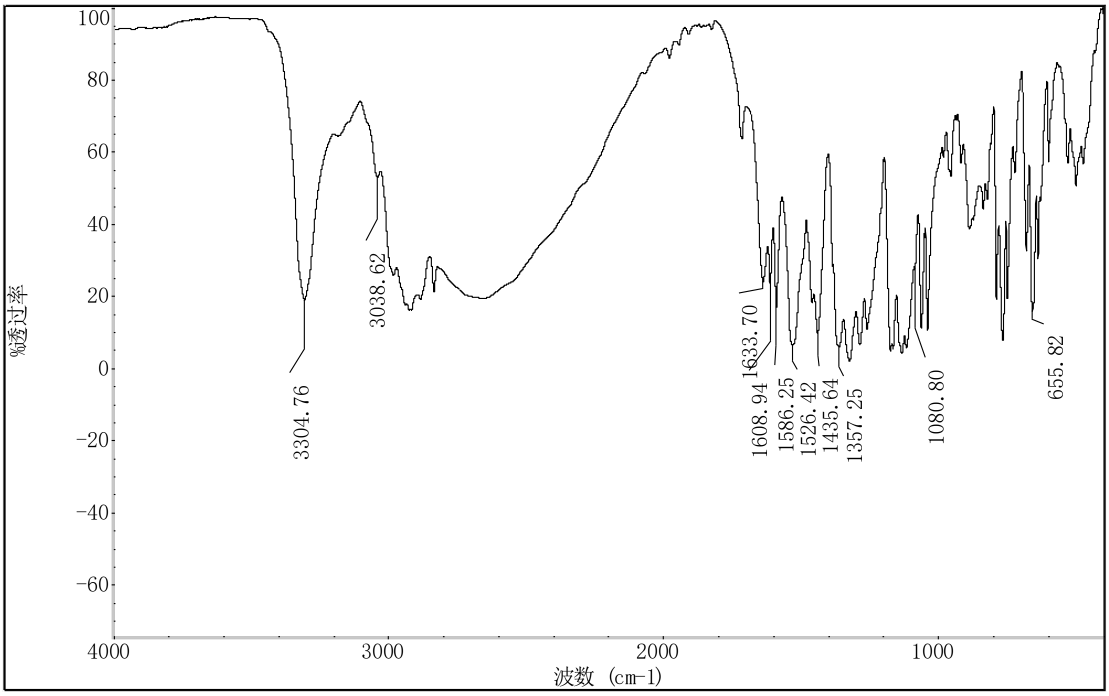

Figure S66. FT-IR spectrum of Compound **6n** (R=*o*-CF_3 Ph).

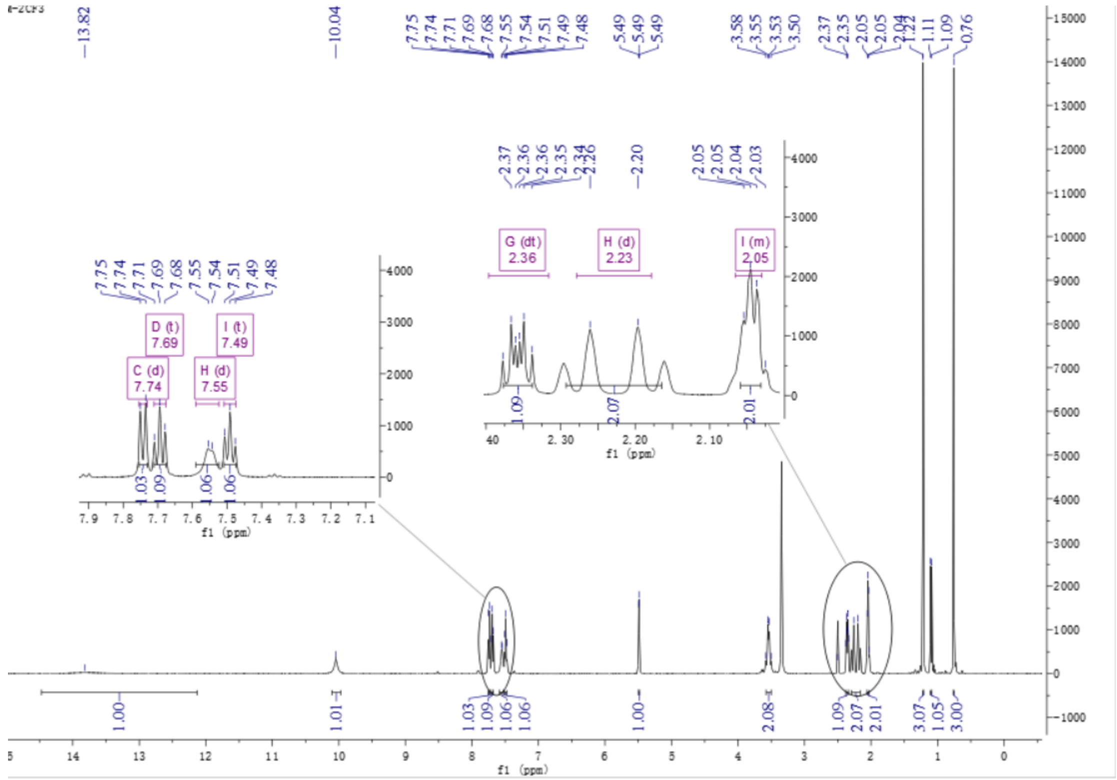

Figure S67. ^{1}H-NMR spectrum of Compound **6n** (R=*o*-CF_3 Ph) in DMSO.

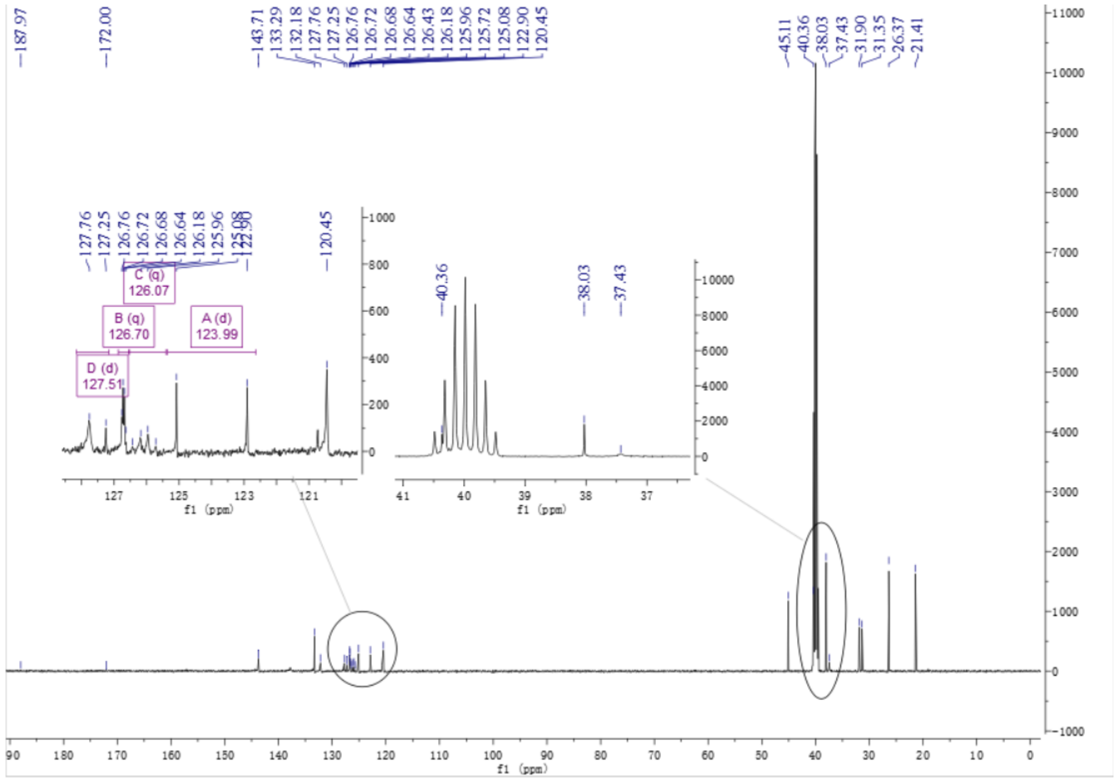

Figure S68. ^{13}C-NMR spectrum of Compound **6n** (R=*o*-CF_3 Ph) in DMSO.

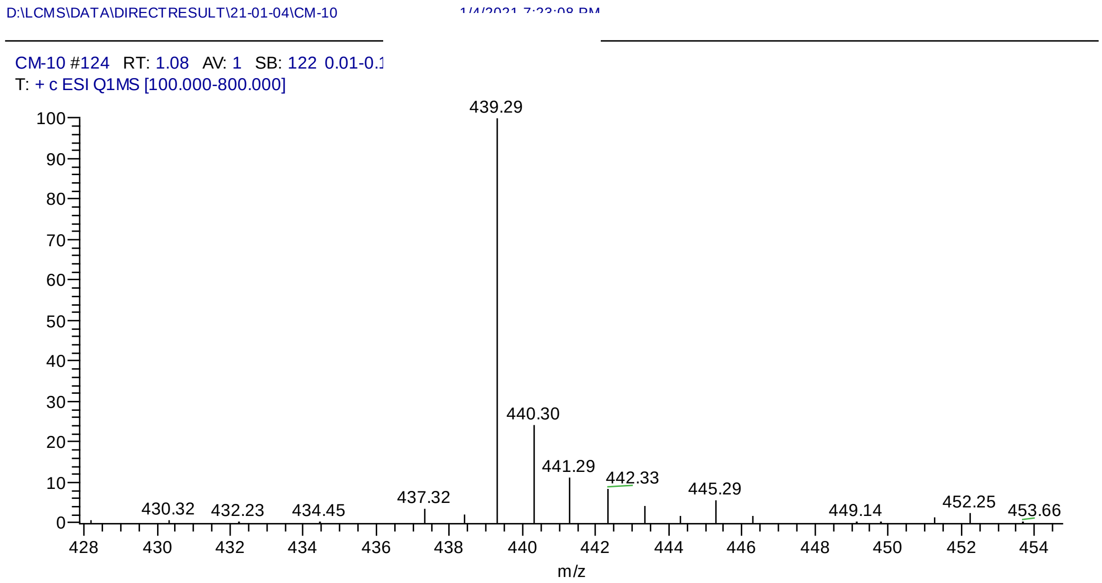

Figure S69. ESI-MS spectrum of Compound **6n** (R=*o*-CF_3 Ph).

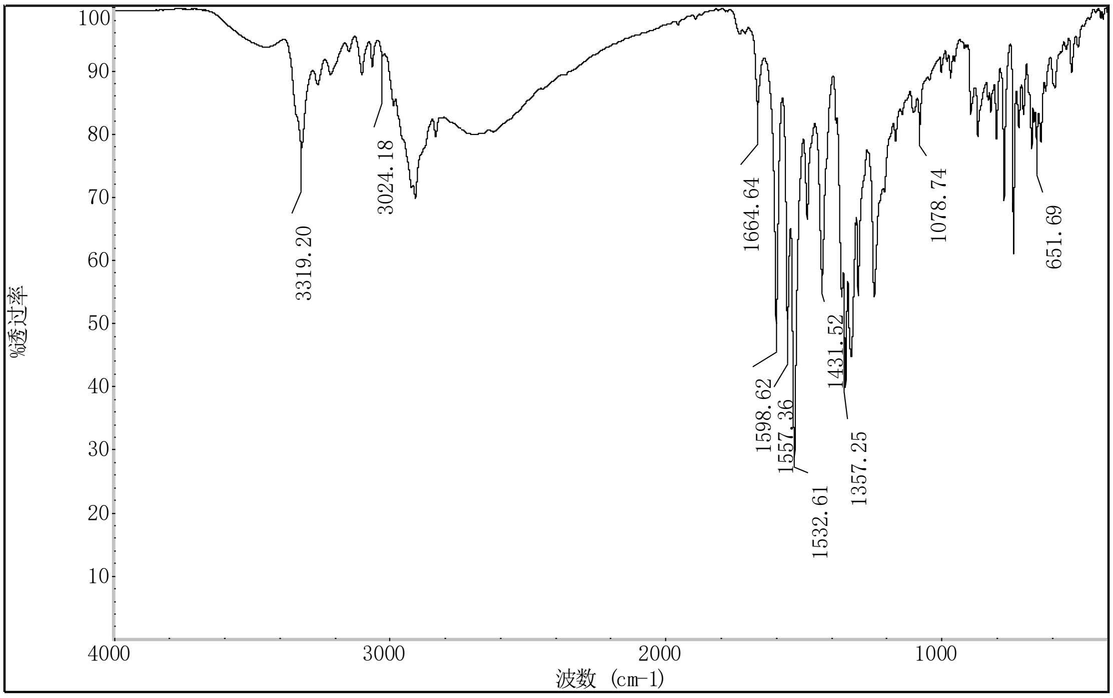

Figure S70. FT-IR spectrum of Compound **6o** (R=*m*-NO_2 Ph).

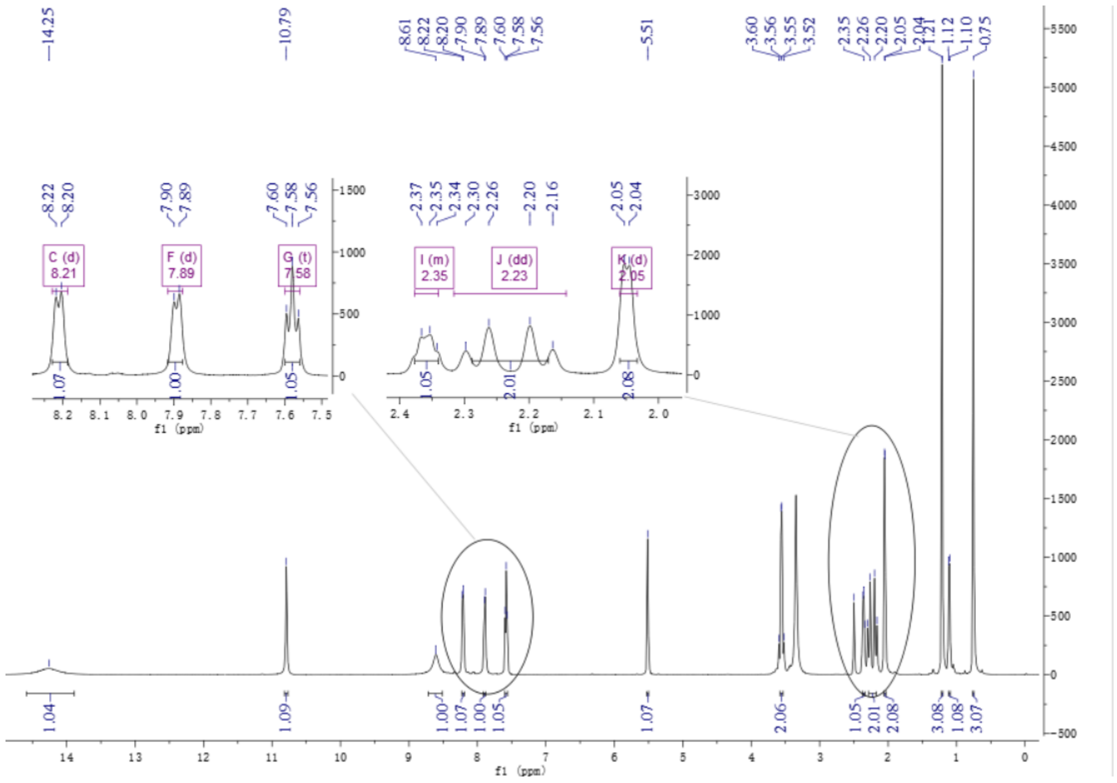

Figure S71. ^{1}H-NMR spectrum of Compound **6o** (R=*m*-NO_2 Ph) in DMSO.

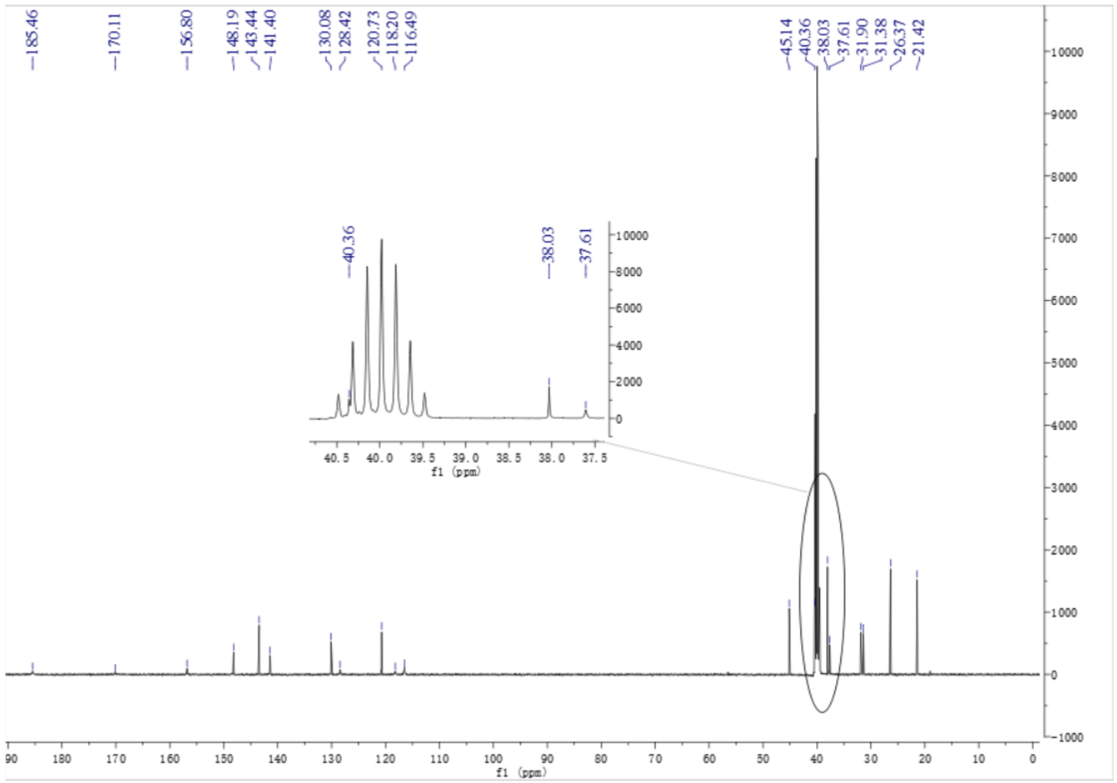

Figure S72. ^{13}C-NMR spectrum of Compound **6o** (R=*m*-NO_2 Ph) in DMSO.

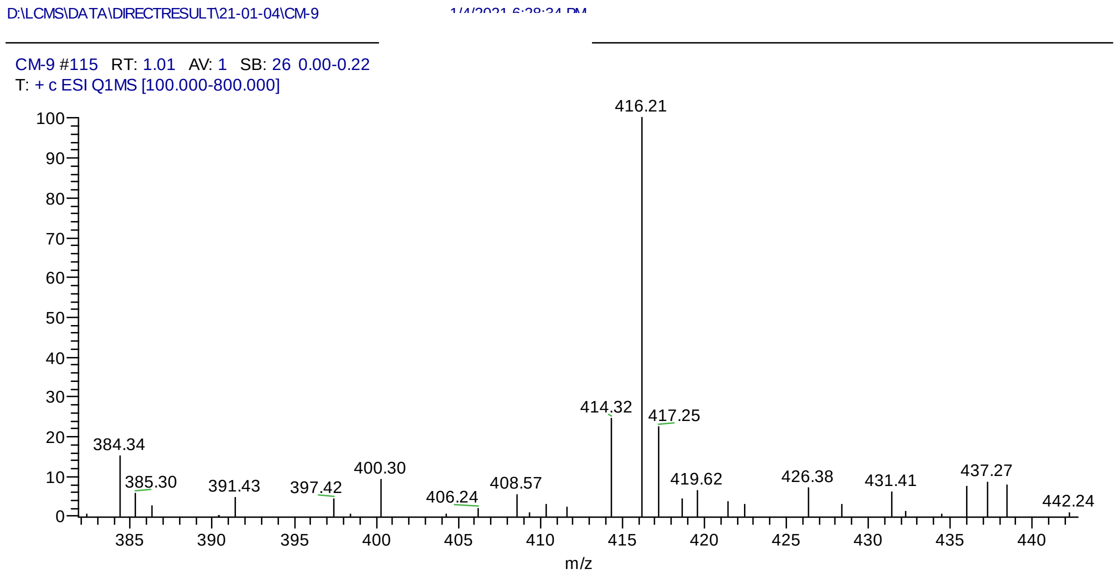

Figure S73. ESI-MS spectrum of Compound **6o** (R=*m*-NO_2 Ph).

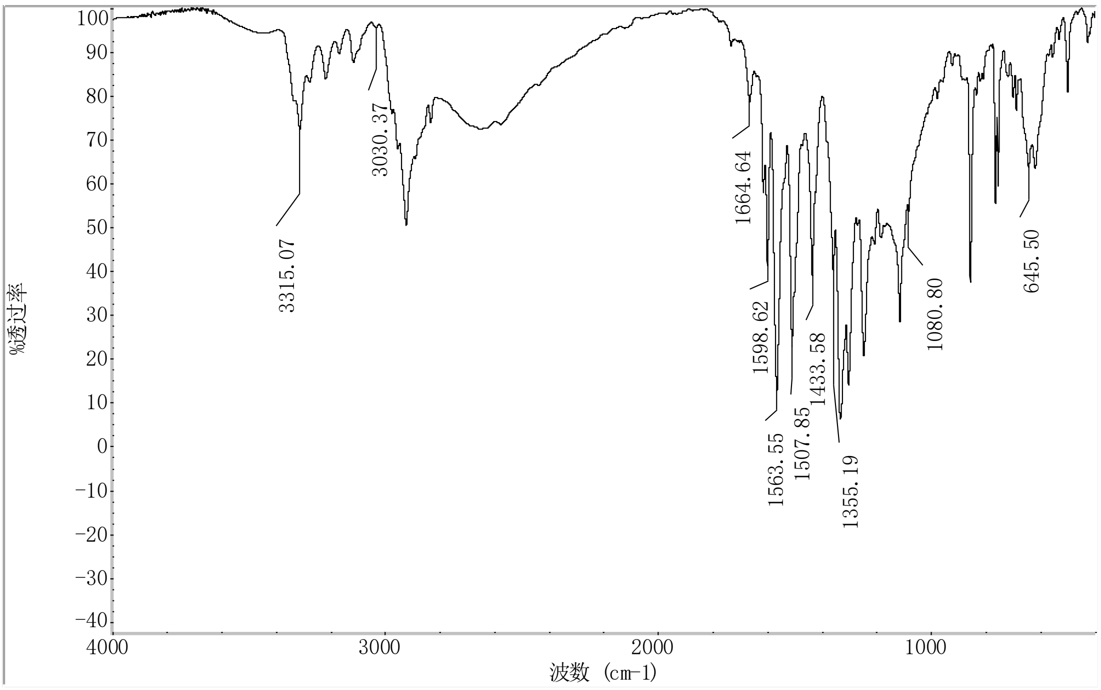

Figure S74. FT-IR spectrum of Compound **6p** (R= *p*-NO_2 Ph).

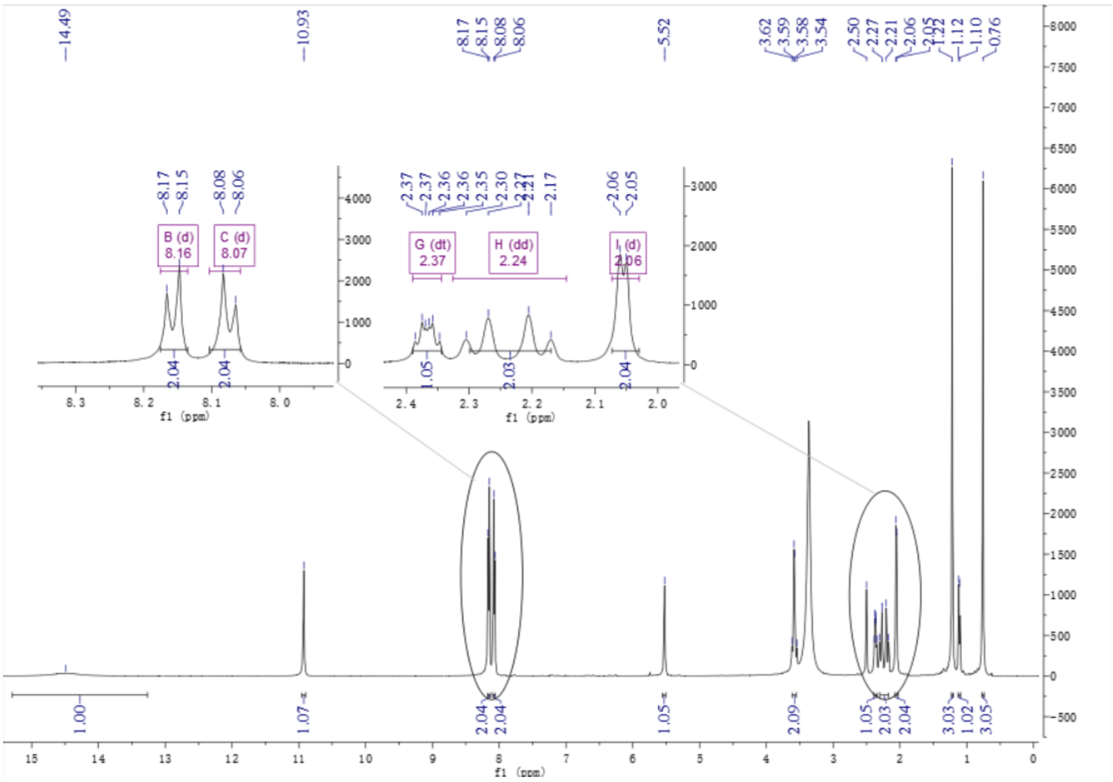

Figure S75. ^{1}H-NMR spectrum of Compound **6p** (R= *p*-NO_2 Ph) in DMSO.

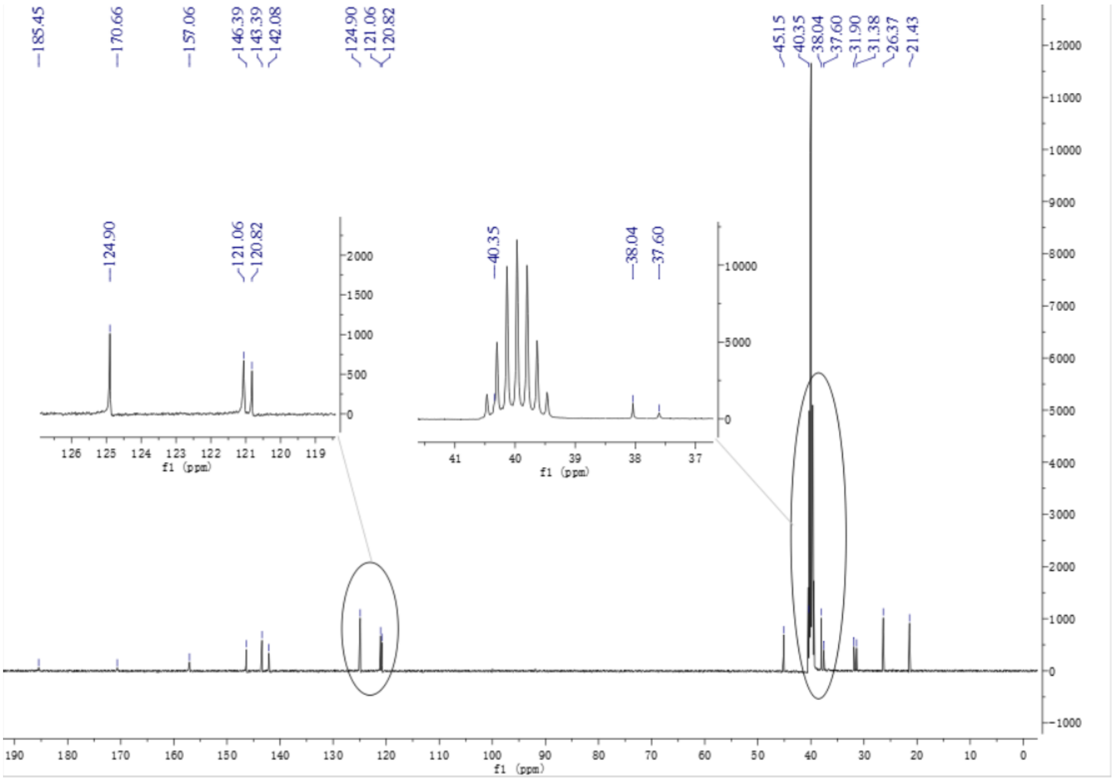

Figure S76. ^{13}C-NMR spectrum of Compound **6p** (R= *p*-NO_2 Ph) in DMSO.

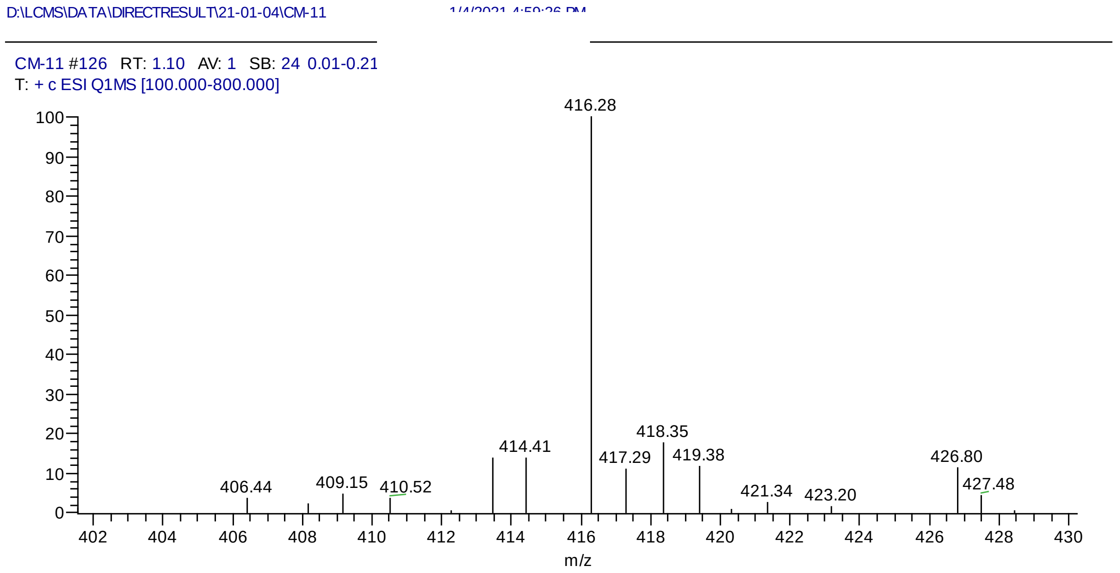

Figure S77. ESI-MS spectrum of Compound **6p** (R= *p*-NO_2 Ph).

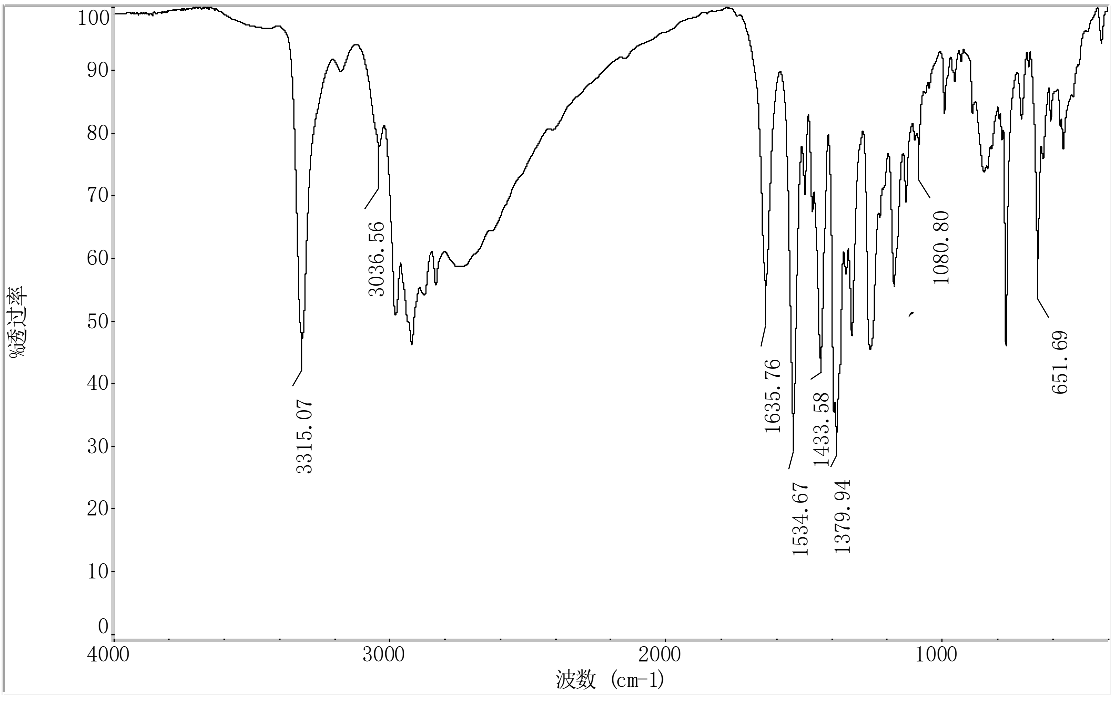

Figure S78. FT-IR spectrum of Compound **6q** (R=*i*-Pr).

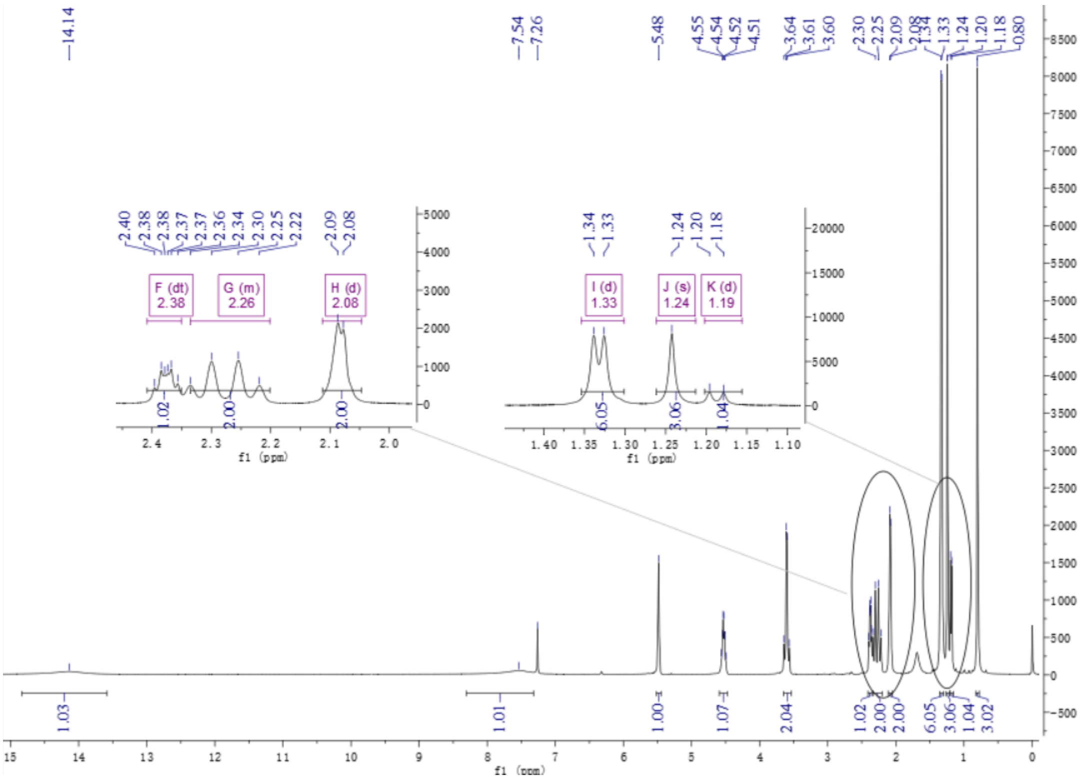

Figure S79. ^{1}H-NMR spectrum of Compound **6q** (R=*i*-Pr) in $CDCl_3$.

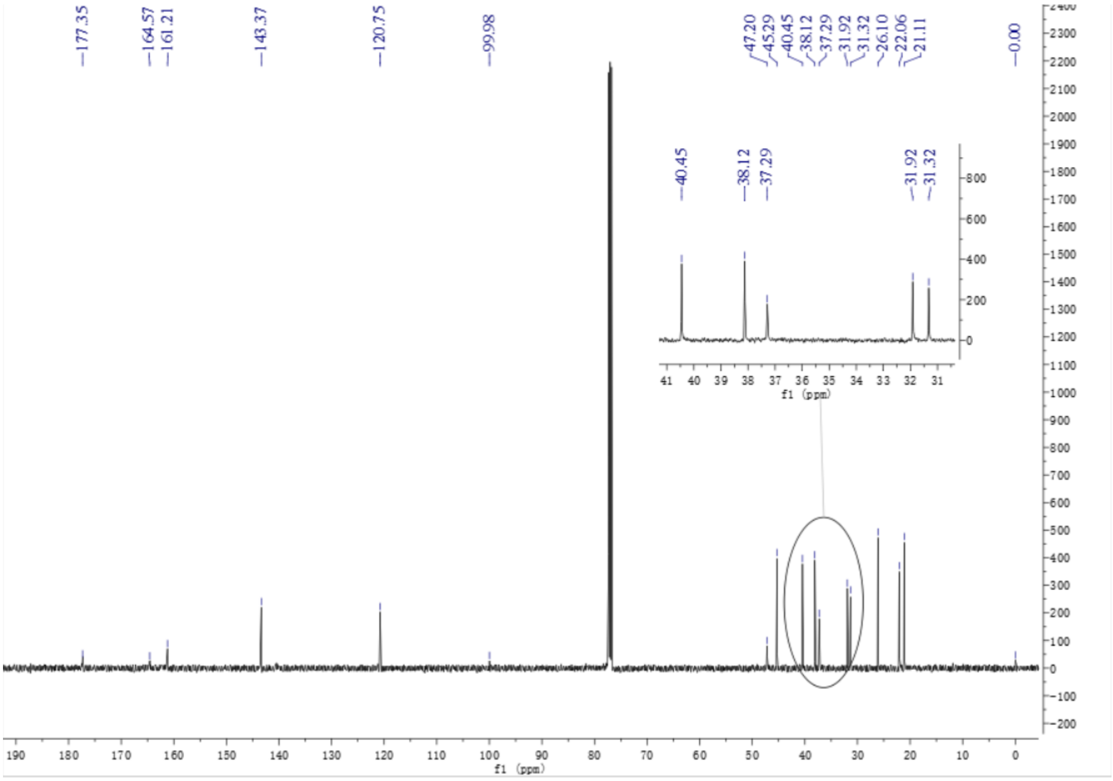

Figure S80. ^{13}C-NMR spectrum of Compound **6q** (R=*i*-Pr) in $CDCl_3$.

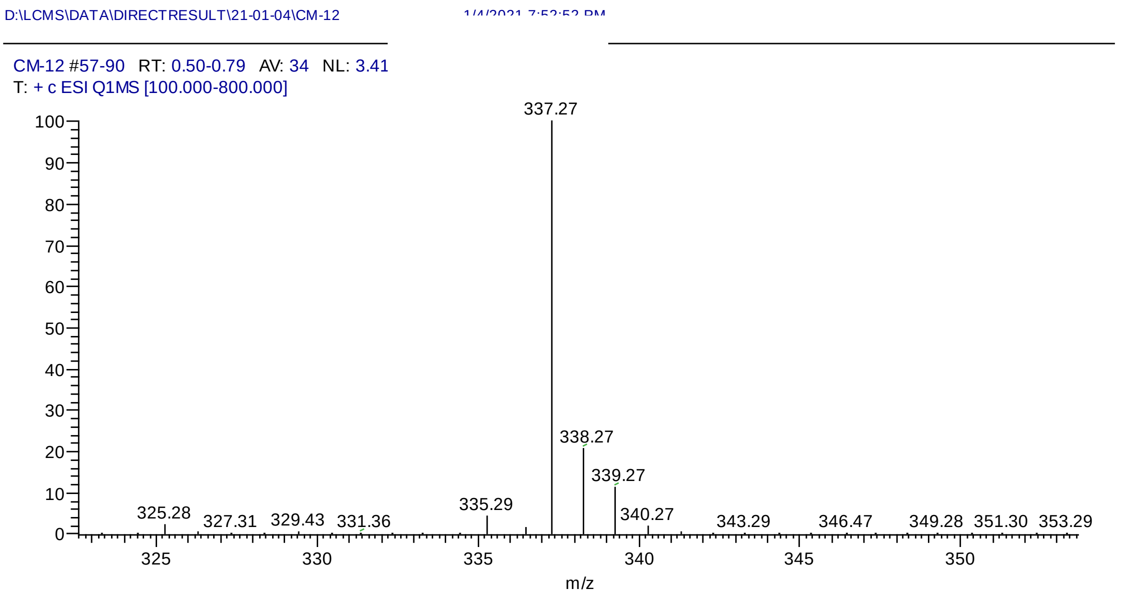

Figure S81. ESI-MS spectrum of Compound **6q** (R=*i*-Pr).

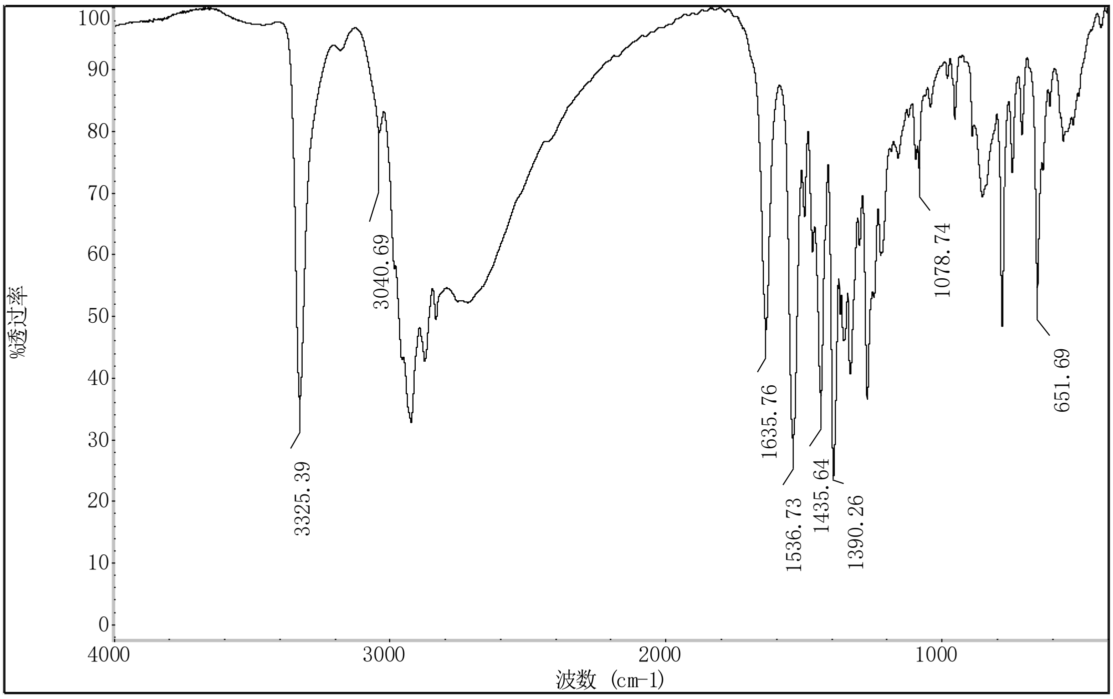

Figure S82. FT-IR spectrum of Compound **6r** (R=*n*-Bu).

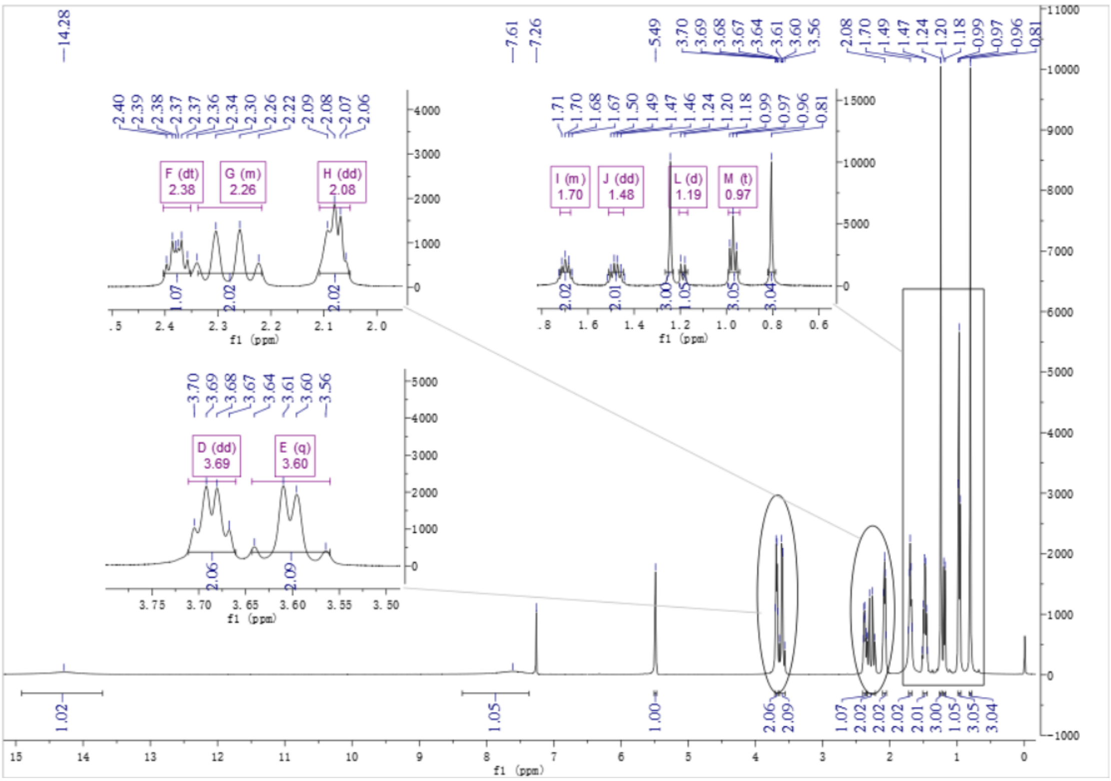

Figure S83. ^{1}H-NMR spectrum of Compound **6r** (R=*n*-Bu) in $CDCl_3$.

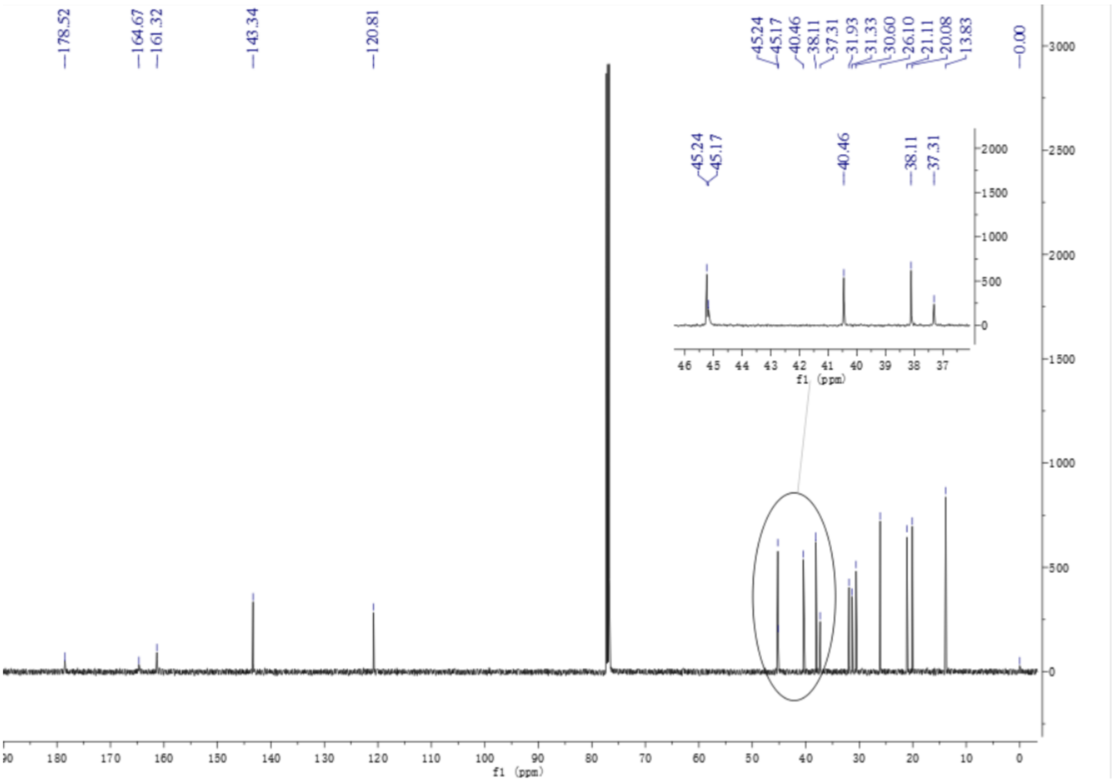

Figure S84. ^{13}C-NMR spectrum of Compound **6r** (R=*n*-Bu) in $CDCl_3$.

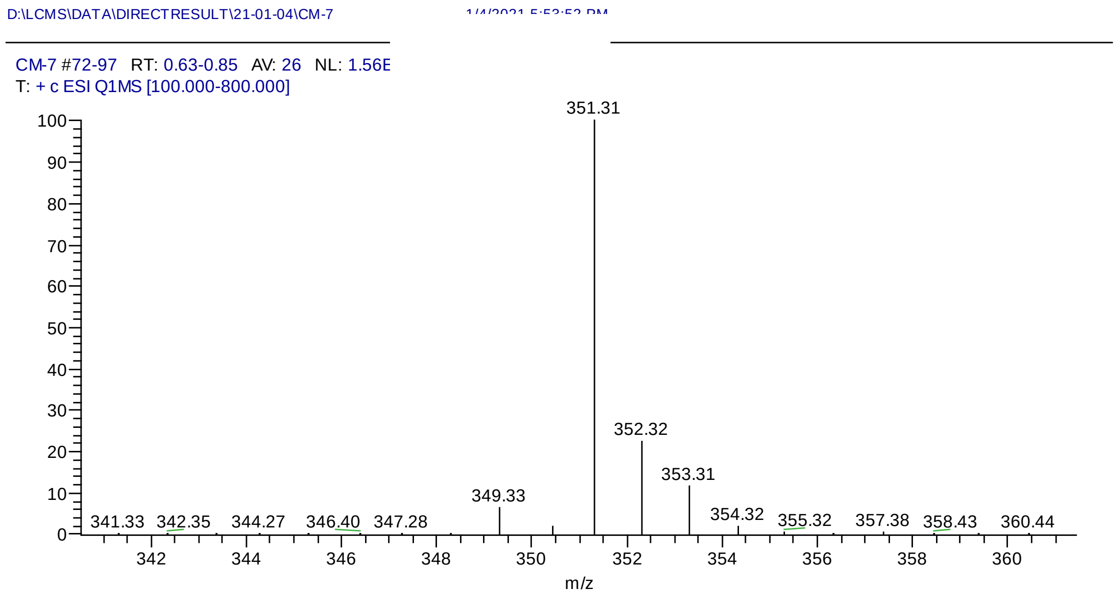

Figure S85. ESI-MS spectrum of Compound **6r** (R=*n*-Bu).
